# Dysregulation of long non-coding RNA gene expression pathways in monocytes of type 2 diabetes patients with cardiovascular disease

**DOI:** 10.1186/s12933-024-02292-1

**Published:** 2024-06-07

**Authors:** Najeeb Halabi, Binitha Thomas, Omar Chidiac, Amal Robay, Julien AbiNahed, Amin Jayyousi, Jassim Al Suwaidi, Martina Bradic, Charbel Abi Khalil

**Affiliations:** 1 Epigenetics Cardiovascular Lab, Department of Genetic Medicine, Weill Cornell Medicine – Qatar, PO box 24144, Doha, Qatar; 2 Bioinformatics Core, Weill Cornell Medicine – Qatar, Doha, Qatar; 3 Department of Genetic Medicine, Weill Cornell Medicine, New York, USA; 4 Technology Innovation Unit, Hamad Medical Corporation, Doha, Qatar; 5 Department of Endocrinology, Hamad Medical Corporation, Doha, Qatar; 6 Heart Hospital, Hamad Medical Corporation, Doha, Qatar; 7 Marie-Josée & Henry R.Kravis Center for Molecular Oncology, Memorial Sloan Kettering, New York, USA; 8 Joan and Sanford I.Weill Department of Medicine, Weill Cornell Medicine, New York, USA

**Keywords:** Long non-coding RNAs, Cardiovascular disease, Type 2 diabetes, Macrovascular disease, Microvascular disease

## Abstract

**Background:**

Monocytes play a central role in the pathophysiology of cardiovascular complications in type 2 diabetes (T2D) patients through different mechanisms. We investigated diabetes-induced changes in lncRNA genes from T2D patients with cardiovascular disease (CVD), long-duration diabetes, and poor glycemic control.

**Methods:**

We performed paired-end RNA sequencing of monocytes from 37 non-diabetes controls and 120 patients with T2D, of whom 86 had either macro or microvascular disease or both. Monocytes were sorted from peripheral blood using flow cytometry; their RNA was purified and sequenced. Alignments and gene counts were obtained with STAR to reference GRCh38 using Gencode (v41) annotations followed by batch correction with CombatSeq. Differential expression analysis was performed with EdgeR and pathway analysis with IPA software focusing on differentially expressed genes (DEGs) with a p-value < 0.05. Additionally, differential co-expression analysis was done with csdR to identify lncRNAs highly associated with diabetes-related expression networks with network centrality scores computed with Igraph and network visualization with Cytoscape.

**Results:**

Comparing T2D vs. non-T2D, we found two significantly upregulated lncRNAs (ENSG00000287255, FDR = 0.017 and ENSG00000289424, FDR = 0.048) and one significantly downregulated lncRNA (ENSG00000276603, FDR = 0.017). Pathway analysis on DEGs revealed networks affecting cellular movement, growth, and development. Co-expression analysis revealed ENSG00000225822 (UBXN7-AS1) as the highest-scoring diabetes network-associated lncRNA. Analysis within T2D patients and CVD revealed one lncRNA upregulated in monocytes from patients with microvascular disease without clinically documented macrovascular disease. (ENSG00000261654, FDR = 0.046). Pathway analysis revealed DEGs involved in networks affecting metabolic and cardiovascular pathologies. Co-expression analysis identified lncRNAs strongly associated with diabetes networks, including ENSG0000028654, ENSG00000261326 (LINC01355), ENSG00000260135 (MMP2-AS1), ENSG00000262097, and ENSG00000241560 (ZBTB20-AS1) when we combined the results from all patients with CVD. Similarly, we identified from co-expression analysis of diabetes patients with a duration ≥ 10 years vs. <10 years two lncRNAs: ENSG00000269019 (HOMER3-AS10) and ENSG00000212719 (LINC02693). The comparison of patients with good vs. poor glycemic control also identified two lncRNAs: ENSG00000245164 (LINC00861) and ENSG00000286313.

**Conclusion:**

We identified dysregulated diabetes-related genes and pathways in monocytes of diabetes patients with cardiovascular complications, including lncRNA genes of unknown function strongly associated with networks of known diabetes genes.

**Supplementary Information:**

The online version contains supplementary material available at 10.1186/s12933-024-02292-1.

## Background

Long non-coding RNAs (lncRNAs) have emerged as key molecules in the intricate regulatory networks governing cellular processes, and their involvement in cardiovascular disease (CVD) has recently garnered significant attention [1]. LncRNAs are a class of RNA molecules exceeding 200 nucleotides in length, lacking protein-coding potential but exhibiting diverse functions in the modulation of gene expression [2]. In the context of CVD, lncRNAs have been implicated in different pathologies, including atherosclerosis, myocardial infarction, and heart failure [3].

In the realm of diabetes, where cardiovascular complications pose a substantial threat, the interplay between lncRNAs and disease progression becomes even more intricate. Mounting evidence suggests that specific lncRNAs play pivotal roles in the complex molecular pathways linking diabetes and cardiovascular complications [4]. For instance, some lncRNAs have been associated with endothelial dysfunction, inflammation, and oxidative stress, which are hallmark features of diabetes and CVD [5]. Moreover, the dysregulation of lncRNAs may contribute to cardiovascular complications in diabetic individuals [6]. These non-coding RNAs can impact key processes such as lipid metabolism, vascular inflammation, and smooth muscle cell proliferation, thus influencing atherogenesis [7].

Monocytes have been implicated in the initiation and progression of cardiovascular complications of diabetes. In individuals with diabetes, monocytes exhibit enhanced adhesion to the endothelium, increased migration into the arterial wall, and a heightened inflammatory response, contributing to endothelial dysfunction and the progression of atherosclerotic lesions [8]. Monocytes are also integral players in the immune response. They can release inflammatory cytokines and reactive oxygen species, exacerbating endothelial dysfunction in microvascular complications of diabetes, such as nephropathy [9] and retinopathy [10].

While several lncRNAs have been identified in diabetes patients, they were often measured from peripheral blood samples containing various cell types, knowing that diabetes-induced cellular dysfunction might differ across those cell types [11]. To investigate the potential role of lncRNAs in the pathogenesis of diabetes and CVD, we conducted a comprehensive analysis of lncRNA expression in monocytes from type 2 diabetes (T2D) patients with different vascular disease phenotypes, diabetes duration, and glycemic control. We also examined the correlation between lncRNA gene expression and protein-coding gene expression to elucidate potentially novel regulatory mechanisms.

## Methods

### Research participants

We recruited 200 patients consecutively and classified them into three groups: (I) Non-diabetes, (II) T2D patients without clinically documented micro- or macro-vascular complications, and (III) T2D with micro- and/or macro-vascular complications. Macrovascular complications were defined as the presence of coronary artery disease (CAD), peripheral vascular disease (PAD), or cerebrovascular disease, as confirmed by the presence of an angiographically proven atherosclerotic disease or Doppler ultrasound [12, 13]. Microvascular complications were defined as the presence of diabetic nephropathy, retinopathy, or neuropathy. All T2D patients had an HbA1c _>_ 6.5%. Diabetic neuropathy was diagnosed based on the vibration perception threshold on the big toe being > 25 V [14]. Diabetic nephropathy was diagnosed according to the presence of both persistent albuminuria and a decline in the estimated glomerular filtration rate (eGFR) [15]. Diabetic retinopathy was diagnosed by fundoscopy [16]. Research participants were recruited from the Heart Hospital and the Department of Endocrinology at Hamad Medical Corporation (HMC) in Doha-Qatar. Non-T2D participants were recruited from the outpatient department at HMC. After potential participants passed an eligibility assessment, clinical data was recorded, and blood was withdrawn from the forearm veins and transported to the Flow Cytometry Facility at Weill Cornell Medicine – Qatar.

### Monocyte sorting and RNA extraction

Each participant underwent a peripheral venous puncture, from which 10 mL of blood was withdrawn. We have previously reported the sorting of circulating monocytes [17]. Briefly, peripheral blood mononuclear cells were isolated from whole blood and stained with mouse anti-human IgG2b CD14–APC and mouse anti-human IgG1 CD16–PE antibodies (BD Bioscience). A FACSAria2 ™ cell sorter was then used for monocyte sorting, with purity assessed after each procedure. RNA was extracted from the sorted monocytes using the Allprep DNA/RNA mini kit (Qiagen). The extracted genetic material was stored at -80 °C before being shipped to the New York Genome Center (NYGC) for sequencing.

### RNA sequencing

Paired-end RNA sequencing was performed at the New York Genome Center. RNA library preparation was done using two different methods, ClonTech SmartSeq, and Illumina TrueSeq, at two different times. ClonTech SmartSeq: cDNA was synthesized from 10ng of good quality total RNA (RIN > 7) using SMART-SEQ v4 Ultra Low Input RNA Kit (ClonTech) according to the manufacturer’s protocol with 8 amplification cycles. cDNA was then purified with a 1:1 by-volume ratio of AMPURE XP beads (Beckman). Full-length cDNA was sheared to an average size of 350 bp fragments using Adaptive Focused Acoustics (AFA) technology (Covaris, LE220). Illumina-compatible libraries were prepared with KAPA Hyper Prep Kit (Roche) and Illumina dual-indexed adapters according to the manufacturer’s specifications with 9 amplification cycles. Illumina TrueSeq: RNA sequencing libraries were prepared using the TruSeqStranded mRNA Library Preparation Kit in accordance with the manufacturer’s instructions. Briefly, 100ng of total RNA was used for the purification and fragmentation of mRNA. Purified mRNA underwent first and second-strand cDNA synthesis. cDNA was then adenylated, ligated to Illumina sequencing adapters, and amplified by PCR (using 10 cycles). For both methods, final libraries were evaluated using PicoGreen (Life Technologies) and Fragment Analyzer (Advanced Analytics). 2 × 50 bp sequencing was done with an Illumina HiSeq 2500 sequencer.

Alignments and gene counts were done with STAR [18] (version 2.7.10a) to reference GRCh38 using Gencode (v41) gene annotations. Read counts were obtained with STAR with a read counted if it overlaps with one and only one gene. Batch correction was done with CombatSeq (part of sva package version 3.50.0) [19] using four batches comprising each separate sequencing run. A multi-dimensional scaling plot of the gene expression profile per sample, pre- and post-batch effect correction is shown in Figure S1.

### Differential expression analysis

Batch-corrected gene counts with less than 20 read counts in more than 70% of samples and genes on the X and Y chromosomes were filtered out of the analysis. This resulted in an analysis set of 2084 lncRNA genes out of 17,617 lncRNA genes observed in the complete dataset. Long non-coding RNAs were analyzed separately from all other genes. TMM normalization and differential expression were done with EdgeR (version 4.0.16) [20] using general linear modeling for covariate correction. The Benjamini-Hochberg procedure was used to control false discovery.

### Pathway analysis

Pathway analysis was done with the IPA software (Qiagen, version 111,725,566) using differentially expressed genes (DEGs) from long non-coding RNA genes with a p-value < 0.05. Significant networks of lncRNAs were obtained using the ‘Network’ in IPA identified using only the lncRNA gene set. Network significance is indicated by IPA software as a score, which is the negative exponent of the right-tailed Fisher’s exact test and indicates the probability that the genes are found as part of the network compared to random chance. Tabular IPA results include several data columns: 1) the ‘Molecules in Network’ column includes both DEGs in our dataset (in bold with arrows showing logFC direction) and other genes, 2: the ‘Score’ column is the significance as above, 3: the ‘Focus Molecules’ are the number of genes in our comparison and the ‘Top Diseases and Pathways’ are those diseases and pathways identified by IPA as linked to the gene columns. Within a comparison, we also aggregated all significant networks together to visualize the combined affected pathways.

### Gene co-expression analysis

To identify lncRNAs co-expressed with previously reported diabetes-related genes, we first generated a list of diabetes-related genes from two different sources. Diabetes-related protein-coding genes were obtained from the Diseases [12] website (https://diseases.jensenlab.org/Search, accessed July 25, 2023) using the integrated database with a score of 3 or higher. Diabetes-related lncRNA genes were obtained from a literature search, as shown in Table S1. As literature names for lncRNAs are non-standardized, we attempted to match the gene names in the literature with the human Ensembl ID, and we were able to do so for 40 out of 72 genes mentioned in the literature (Table S2). The lack of standardized naming limits the literature analysis, an issue previously noted in recent lncRNA reviews [21, 22].

Co-expression analysis was performed with the csdR package (version 1.8.0) [23] using fitted counts and 10-1000 bootstrap iterations. Protein coding and lncRNA genes were analyzed together. Only non-sex chromosome genes with at least 20 reads in at least 70% of samples were analyzed. The analysis data set included 2084 lncRNA genes and 11,248 protein-coding genes. Furthermore, to focus on potentially novel lncRNAs that interact with the diabetes network, we limited our analysis to only 220 known protein-coding diabetes genes and all lncRNAs passing the above filter. The top 5000 correlations for each of the C (conserved), S (specific), and D (differentiated) types were combined into one network. The combined network was then further filtered to exclude edges with a maximum score of less than 1 and genes longer than one node away from a known diabetes gene to obtain a core diabetes-related network. Node degree and node betweenness centrality (weighted by maximum score value) were calculated with Igraph (version 2.0.2). Node degree is the number of edges between one node and another node. Node betweenness is proportional to the number of shortest paths passing through that node calculated for every pair of nodes in the network and is a measure of the centrality of nodes [24]. LncRNA genes were prioritized and considered highly associated if they were in the top 10 by degree or by betweenness scores. The diabetes-associated network was exported to Cytoscape (version 3.10.0) [25], where additional network visualization was done. To simplify network complexity for visualization, we only show the union of the top ten genes with the highest degree, the top 10 genes with the highest betweenness score, and all S/D edges with maximum value score in the 70% quantile or more.

### Analysis plan

**Fig. 1:**
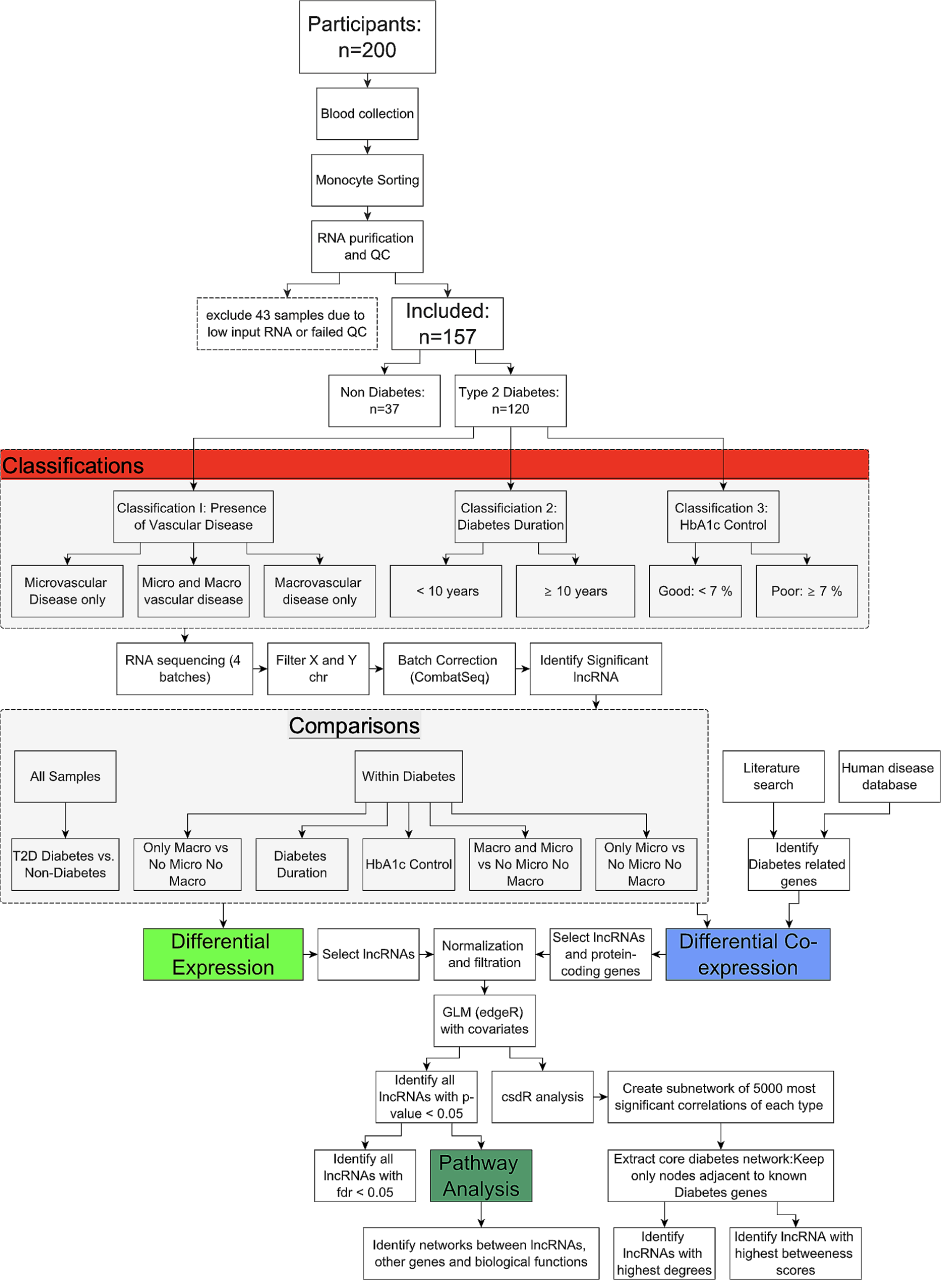
Flow chart showing the major analysis steps for identifying significant lncRNA associated with diabetes or diabetes complications

Figure 1 shows a flowchart of the analysis plan and key steps. We first compared diabetes participants to non-diabetes patients. Further, we performed analyses within diabetes patients according to the presence of cardiovascular complications: (a) patients with macro and microvascular disease vs. no macro or microvascular disease, (b) patients with microvascular disease vs. no macro or microvascular disease, and (c) patients with macrovascular disease vs. no macro or microvascular disease. Furthermore, we analyzed diabetes patients according to diabetes duration, which was stratified into < 10 years or ≥ 10 years. Finally, we analyzed diabetes patients according to glycemic control, defined as good control (HbA1c < 7%) and poor control (HbA1c ≥ 7%). The following analysis was done within every comparison: differential expression, pathway analysis, and co-expression correlation analysis.

## Results

### Clinical characteristics of study patients

Within the 200 research participants recruited in this study, the RNA sequencing of 157 participants (120 patients with and 37 without diabetes) passed QC and was further included in the analyses. Baseline characteristics of those participants are presented in Table 1. Diabetes patients were older (57 (8) vs. 50 (12), *p* = 0.001). There was no significant difference in the gender distribution or BMI. However, patients with diabetes had a significantly higher prevalence of hypertension and dyslipidemia, hence taking more cardioprotective drugs such as ACE inhibitors/ARBs and aspirin. As expected, creatinine was higher in patients with diabetes (82 (46) vs. 161 (180), *p* = 0.014). In diabetes patients, the mean (SD) HbA1c was 8.5 (2) %, the duration of diabetes was 15.8 (8) years, and 54% were on insulin. Eighty-six patients (72%) had either macro or microvascular disease or both. Twenty-two had no cardiovascular complications.

**Table 1:** Baseline characteristics of all research participants. Data is shown as mean (SD) or number (percentage). ACEi: ACE inhibitors, ARBs: Angiotensin receptor blockers

Patient Characteristics	Non-Diabetes *N* = 37	Diabetes *N* = 120	*P*-value
Male gender (n, %) **	19 (51.4)	71 (59.2)	0.516
Age (mean (SD)) **	50.89 (12.85)	57.20 (8.93)	0.001
BMI (mean (SD))	32.02 (7.52)	31.96 (6.30)	0.961
Hypertension (n, %)	19 (51.4)	94 (79.0)	0.002
Dyslipidemi**a** (n, %)	13 (35.1)	95 (79.8)	< 0.001
Statins (n, %)	11 (29.7)	76 (65.0)	< 0.001
Creatinine (mean (SD))	82 (46)	161 (182)	0.014
Smokers (n, %)	11 (30.6)	9(8.0)	0.002
HbA1c % (mean (SD))	5.73 (0.43)	8.56 (2.06)	< 0.001
ACEi/ARBs (n, %)	13 (35.1)	68 (58.1)	0.024
Aspirin (n, %)	5 (13.5)	38 (32.5)	0.042
Insulin (n, %)***	NA	64 (54.2)	
Diabetes duration***			
All patients (Mean (SD)		15.88 (8.36)	
< 10 years (n, %)		24 (20)	
≥ 10 years (n, %)		96 (80)	
**Diabetes control** (%)			
Good		30 (25)	
Poor		90 (75)	
**Vascular disease** (n)			
Both macrovascular and microvascular disease		25	
No macrovascular/microvascular disease		22	
Unknown vascular status		12	
**Micro or macrovascular disease**			
Only macrovascular disease		3	
Only microvascular disease		58	

* Covariate used only when comparing between nondiabetesand diabetes samples

** Covariate used between non-diabates and diabetes andwithin diabetes samples

*** Covariate used only within diabetes samples

### Comparison of diabetes to non-diabetes participants

**Fig. 2:**
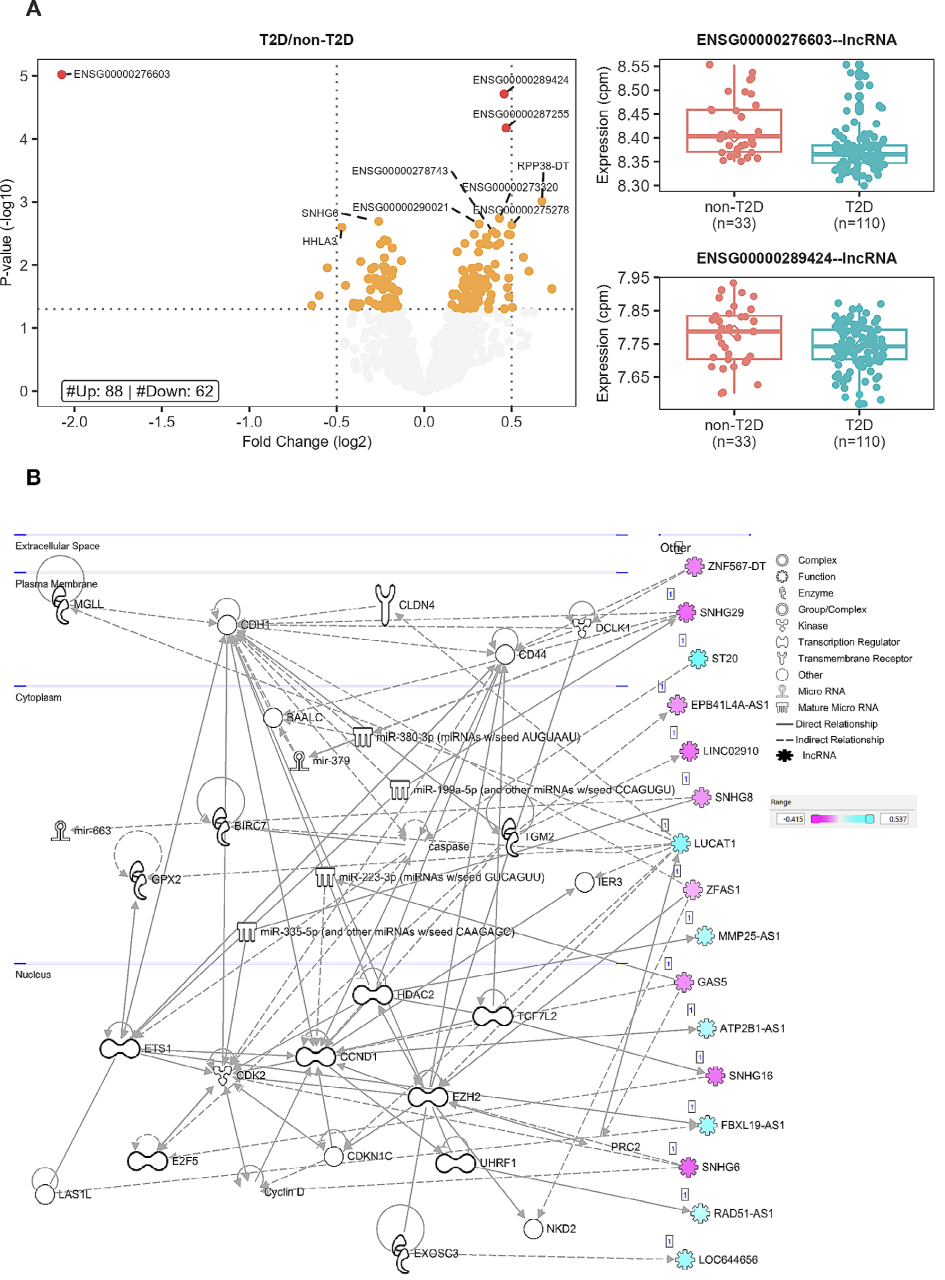
Diabetes vs. non-Diabetes lncRNA differential expression and pathway analysis: (**A**) Volcano plot and box plots showing the results of the differential expression analysis. The volcano plot shows significant genes at an fdr < 0.05 in red, significant genes at a p-value < 0.05 in orange, and non-significant genes in gray. The vertical dotted lines are set at abs (FC) = 0.5 and the horizontal dotted line at a– log10(0.05). The top significant genes by p-value are labeled. The box plots show the expression cpm for the top 2 significant genes. (**B**) IPA network diagram of the merged networks in B. Gene colors indicate the logFC from our analysis as indicated in the legend

**Fig. 3:**
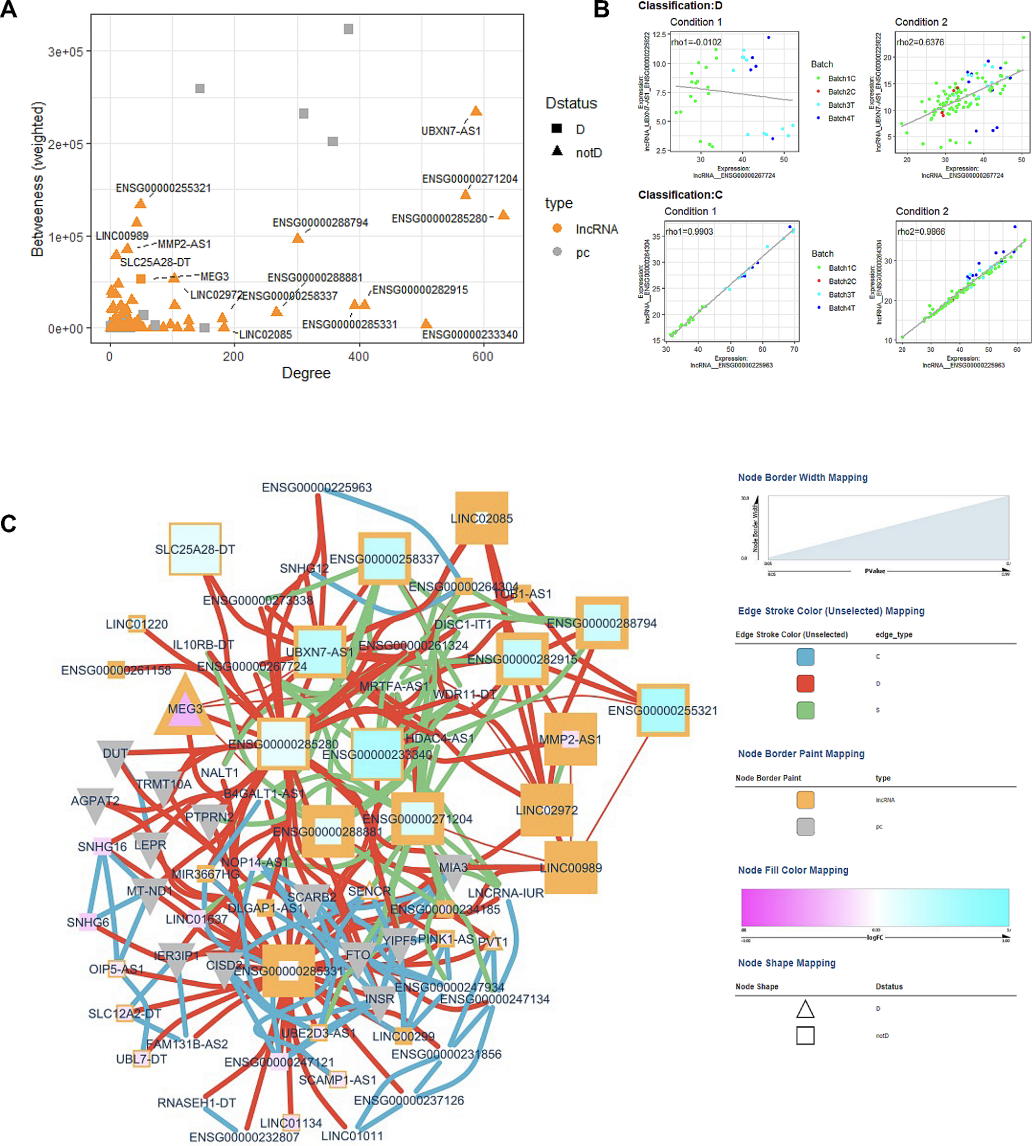
Diabetes vs. non-diabetes co-expression analysis: (**A**) Scatter plot of Degree vs. Betweenness score for each gene in the diabetes-related network. The top 10 lncRNA genes by a degree or Betweenness score are labeled. (**B**) Correlation plots of two different gene pairs. As the legend indicates, each point is a sample belonging to one of four batches. The classification and regression coefficients (rho1, rho2) from the csdR results are displayed on the plot. (**C**) Visualization of the diabetes-related network shows the correlations between different genes. As indicated in the legend, node edges are proportional to the p-value, edge color corresponds to the C, D, and S types, node border color refers to lncRNA or pc genes, node interior color refers to the logFC, and node shape to lncRNA or protein-coding genes

Differential expression analysis of lncRNA genes with covariate correction was performed on 120 diabetes vs. 33 non-diabetes patients. This resulted in identifying 84 upregulated and 64 downregulated lncRNA genes (Table S3). Three genes (ENSG00000276603, ENSG00000289424, ENSG000000287255) were highly significant with an FDR-adjusted p-value < 0.05, as indicated in Fig. 2A. Pathway analysis on the set of significant genes shows a role in several cell development, cell growth, and immune response networks (Fig. 2B, Table S4). We performed a co-expression analysis to further identify potentially novel diabetes-related lncRNA genes. We identified 14 potentially novel lncRNA genes within the same co-expression network as known diabetes genes (Fig. 3A**).** The lncRNA (ENSG00000225822, UBXN7-AS1) had the highest betweenness score (Fig. 3A), while ENSG00000285280 had the highest degree. Two representative correlation plots are shown in Fig. 3B, showing both D and C classifications. The complex correlation network of the diabetes-related genes is shown in Fig. 3C. D, S, and C interaction networks are present. One previously known lncRNA, MEG3, is within this core diabetes-related network.

### Comparison of diabetes participants according to the presence of cardiovascular complications

#### a. Patients with macro and microvascular disease vs. no vascular disease

**Fig. 4:**
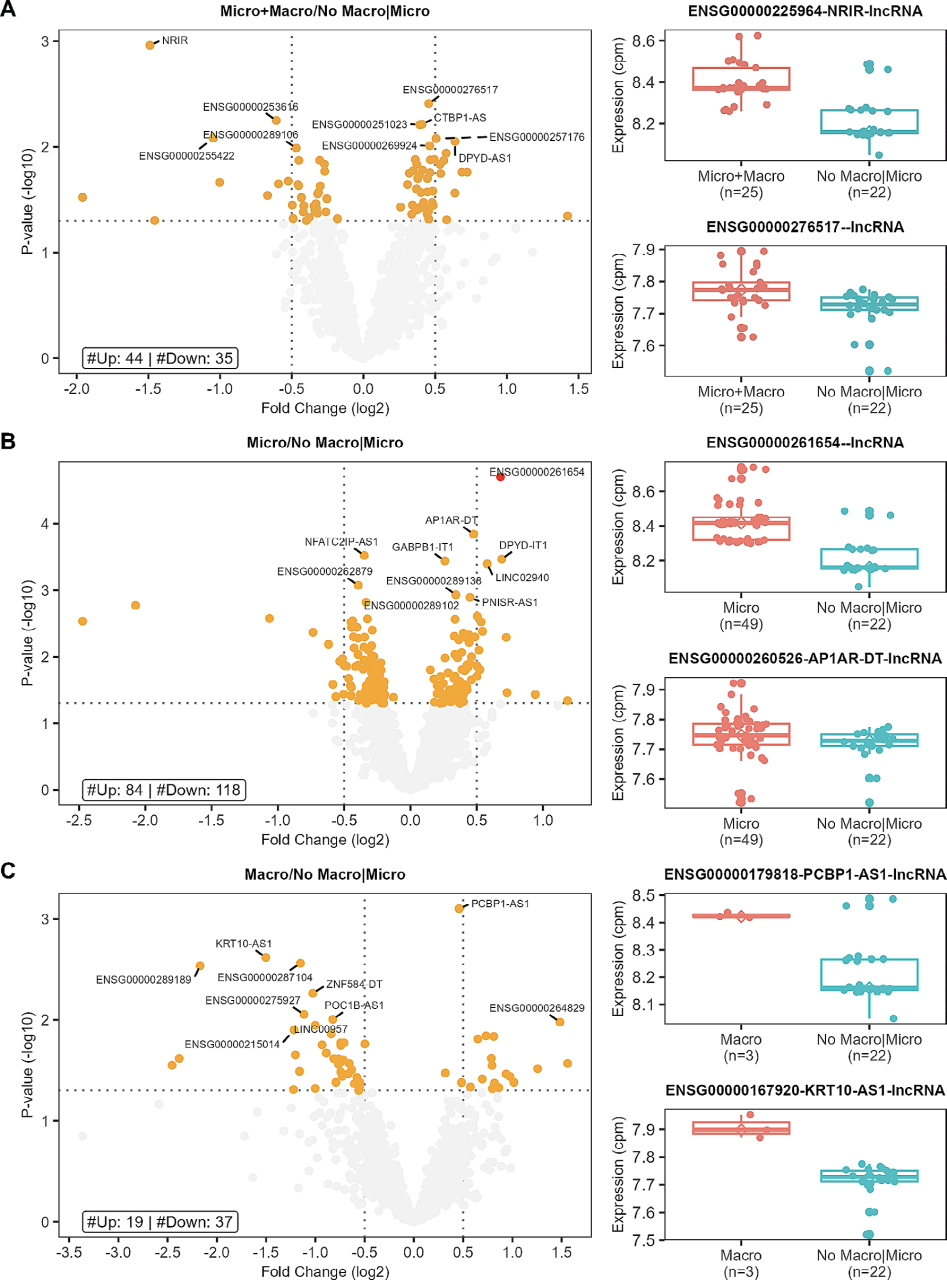
Vascular Disease lncRNA Differential Expression: Volcano plot and box plots showing the results of the differential expression analysis for patients with (**A**) microvascular and macrovascular disease (micro + macro), (**B**) patients with only microvascular disease (micro), and (**C**) patients with only macrovascular disease vs. patients with no micro or macrovascular disease (No macro/micro). The volcano plot shows significant genes at an FDR < 0.05 in red, significant genes at a p-value < 0.05 in orange, and non-significant genes in gray. The top significant genes by p-value are labeled. The box plots show the expression cpm for the top 2 significant genes. The vertical dotted lines are set at abs (FC) = 0.5 and the horizontal dotted line at a– log10(0.05)

**Fig. 5:**
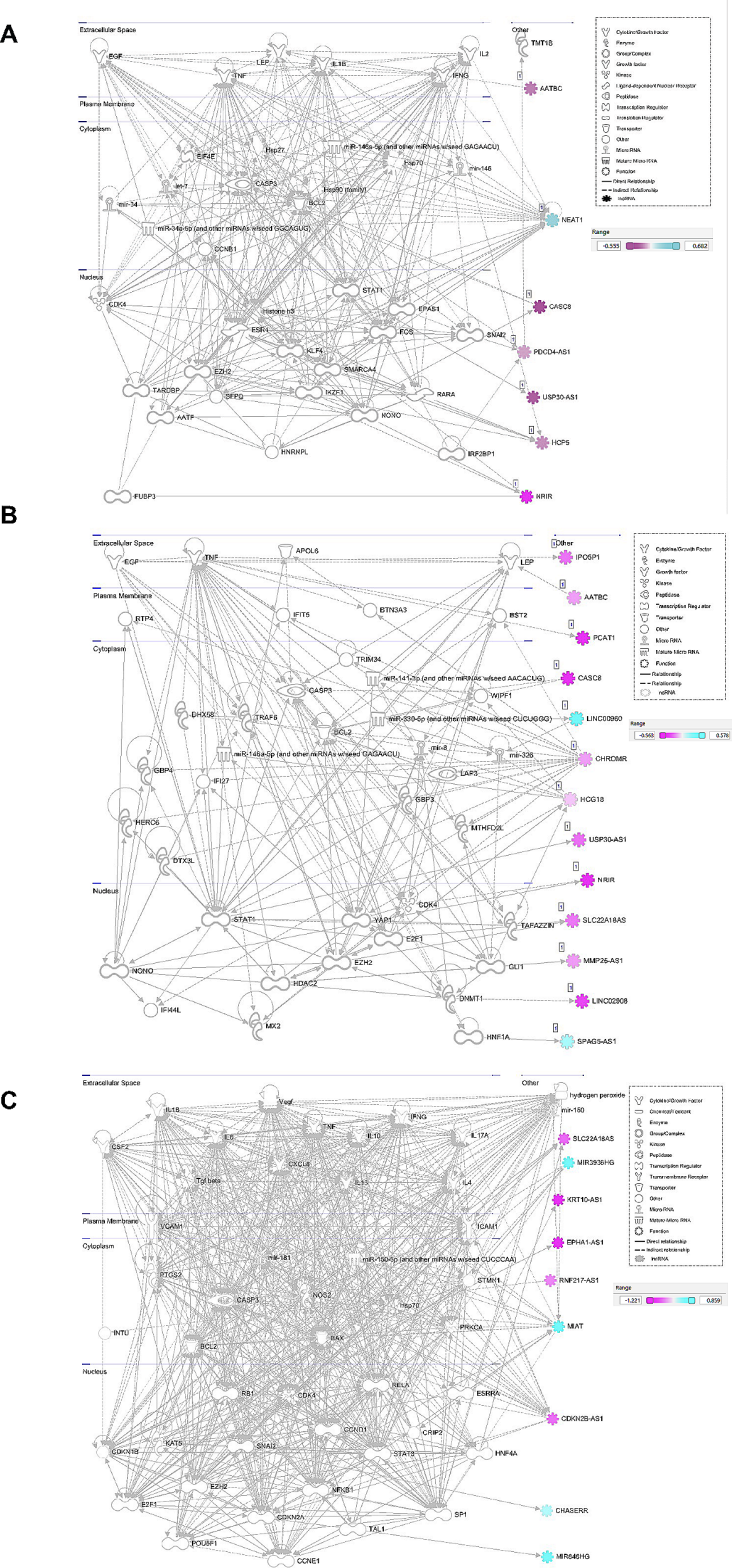
IPA network diagrams of significant differentially expressed genes for (**A**) patients with macrovascular and microvascular disease, (**B**) patients with microvascular disease, and (**C**) patients with only macrovascular disease, all compared to patients without vascular disease

**Fig. 6:**
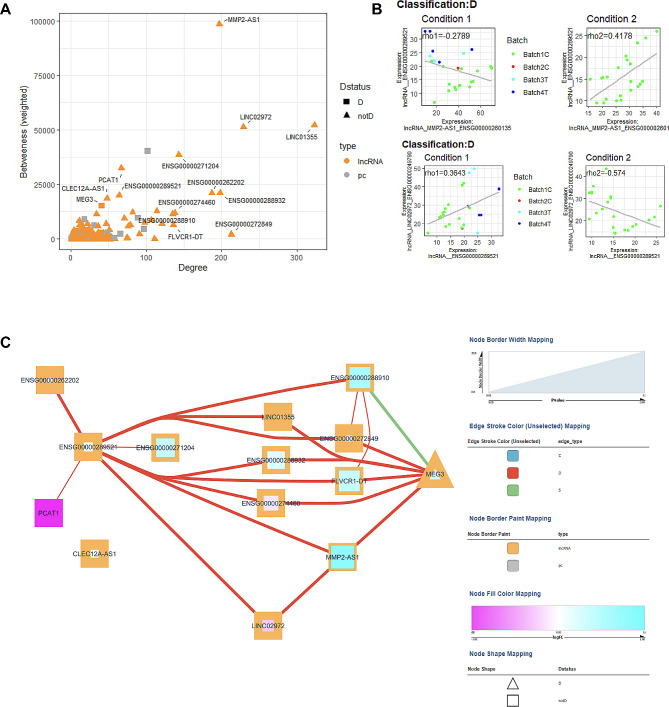
Macro and microvascular disease vs. no vascular disease co-expression analysis. (**A**) Scatter plot of Degree vs. Betweenness score for each gene in the diabetes-related network. The top 10 lncRNA genes by a degree or Betweenness score are labeled. (**B**) Correlation plots of two different gene pairs. As the legend indicates, each point is a sample belonging to one of four batches. The classification and regression coefficients (rho1, rho2) from the csdR results are displayed on the plot. (**C**) Visualization of the diabetes-related network shows the correlations between different genes. As indicated in the legend, node edges are proportional to the p-value, edge color corresponds to the C, D, and S types, node border color refers to lncRNA or pc genes, node interior color refers to the logFC, and node shape to lncRNA or protein-coding genes

Twenty-five patients with macro and microvascular disease were compared to 22 diabetes patients without clinically documented CVD. Differential expression identified 44 upregulated and 35 downregulated lncRNA genes (Fig. 4A, Table S5). NRIR, a known diabetes-related gene, has the lowest p-value at 0.0011 and a logFC of– 1.5, indicating high downregulation in those with both micro and macrovascular disease. Pathway analysis (Fig. 5A, Table S8) also identified networks of significant lncRNA genes (HCP5, NEAT1, USP3-AS1, AATBC, CASC8, PDCD4-AS1, NRIR) affecting metabolism, CVD, and ophthalmic disease among other networks. Co-expression analysis also identified 13 lncRNAs associated with known Diabetes genes (Fig. 6A**)**, combining the lncRNAs with the top 10 highest degrees or the top 10 highest betweenness scores. The highest degree lncRNA is ENSG00000261326 (LINC01355), and the highest betweenness score lncRNA is MMP2-AS1 (Fig. 6A**)**. Two representative correlation plots are shown in Fig. 6B, which shows both D classifications. Figure 6C is a simplified visualization of the core diabetes-related network showing mostly D correlations.

#### b. Patients with microvascular disease vs. no vascular disease

**Fig. 7:**
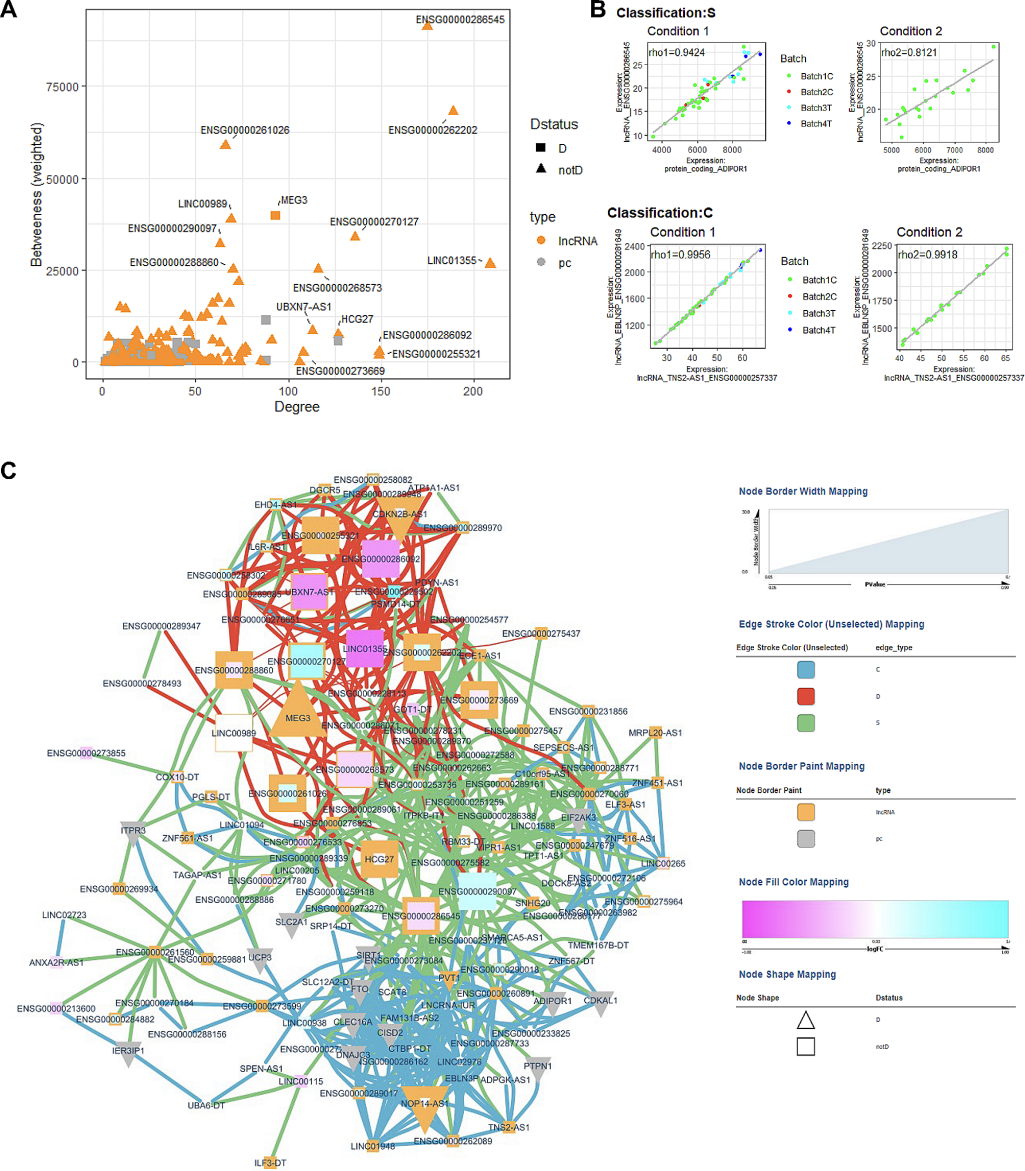
Microvascular disease vs. no vascular disease co-expression analysis. (**A)** Scatter plot of Degree vs. Betweenness score for each gene in the diabetes-related network. The top 10 lncRNA genes by a degree or Betweenness score are labeled. (**B**) Correlation plots of two different gene pairs. As the legend indicates, each point is a sample belonging to one of four batches. The classification and regression coefficients (rho1, rho2) from the csdR results are displayed on the plot. (**C**) Visualization of the diabetes-related network shows the correlations between different genes. As indicated in the legend, node edges are proportional to the p-value, edge color corresponds to the C, D, and S types, node border color refers to lncRNA or pc genes, node interior color refers to the logFC, and node shape to lncRNA or pc genes

Fifty-eight patients with microvascular disease and no clinically documented macrovascular disease were compared to 22 diabetes patients without CVD. Differential expression identified 82 upregulated and 116 downregulated lncRNA genes (Fig. 4B, Table S6), including one lncRNA gene with an FDR < 0.05 (ENSG00000261654, RP11_96K194). Pathway analysis (Fig. 5B, Table S8) also identified networks of significant lncRNA genes (AATBC, CASC8, CHROMR, IPO5P1, MMP25-AS1, NRIR, PCAT1, SLC22A18AS, USP30-AS1, LINC02908, SPAG5-AS1, LINC00960 and HCG18) affecting immune responses, auditory, neurological and CVD. Expression correlation analysis also identified 14 lncRNA genes associated with known Diabetes genes (Fig. 7A), combining the top 10 highest genes by degree and betweenness score. The highest degree lncRNA is LINC01355, and the highest betweenness score lncRNA is ENSG00000286545. Two classifications comprising this network are S and C, as shown in Fig. 7B. Visualization of the core diabetes-related network (Fig. 7C) shows a complex interaction of both D and S with three known Diabetes lncRNA genes (MEG3, CDNB2B-AS1, and NOP14-AS1) interacting within this network.

#### c. Patients with macrovascular disease vs. no vascular disease

**Fig. 8:**
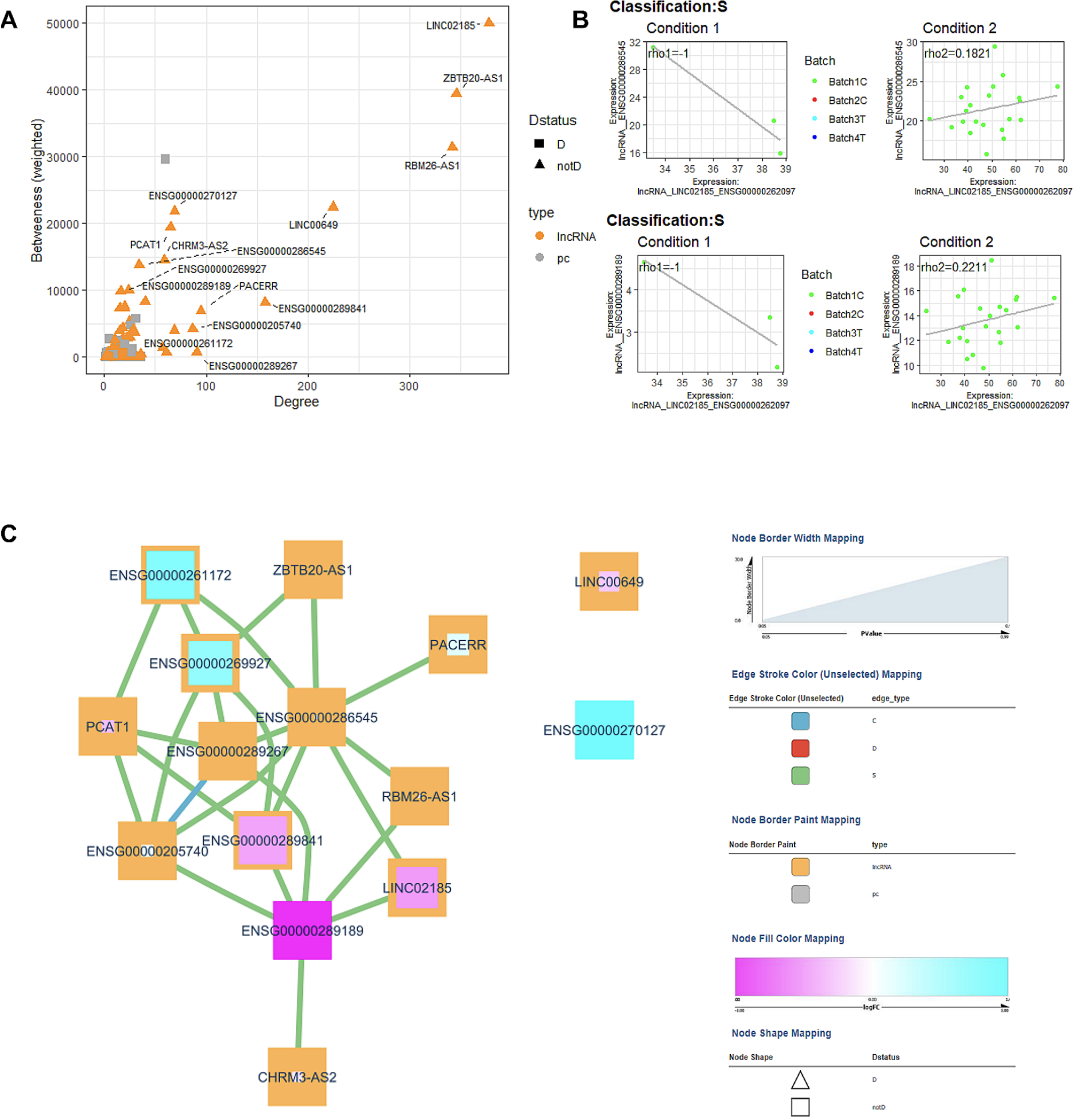
Macrovascular disease vs. no vascular disease co-expression analysis. (**A**) Scatter plot of Degree vs. Betweenness score for each gene in the diabetes-related network. The top 10 lncRNA genes by a degree or Betweenness score are labeled. (**B**) Correlation plots of two different gene pairs. As the legend indicates, each point is a sample belonging to one of four batches. The classification and regression coefficients (rho1, rho2) from the csdR results are displayed on the plot. (**C**) Visualization of the diabetes-related network shows the correlations between different genes. As indicated in the legend, node edges are proportional to the p-value, edge color corresponds to the C, D, and S types, node border color refers to lncRNA or pc genes, node interior color refers to the logFC, and node shape to lncRNA or protein-coding genes

Three patients with macrovascular disease and no clinically documented microvascular disease were compared to 22 diabetes patients without CVD. Differential expression identified 18 upregulated and 35 downregulated lncRNA genes (Fig. 4C, Table S7). Pathway analysis (Fig. 5C, Table S8) also identified networks of significant lncRNA genes (CDKN2B-AS1, MIAT, MIR646HG, RNF217-AS1, EPHA1-AS1, KRT10-AS1, CHASERR, SLC22A18AS, and MIR3936HG) affecting CVD and cell cycle-related networks among other functions. Expression correlation analysis also identified 16 genes associated with known Diabetes genes (Fig. 8A**).** The highest degree and betweenness lncRNA is ENSG00000262097 (LINC02185), with the second highest being ENSG00000241560 (ZBTB20-AS1). Two correlation plots (Fig. 8B) show S classifications, although there are only three samples for the macrovascular condition (condition 1). The core diabetes network, visualized in Fig. 8C, is composed mainly of S relationships.

### Comparison of diabetes patients according to diabetes duration

**Fig. 9:**
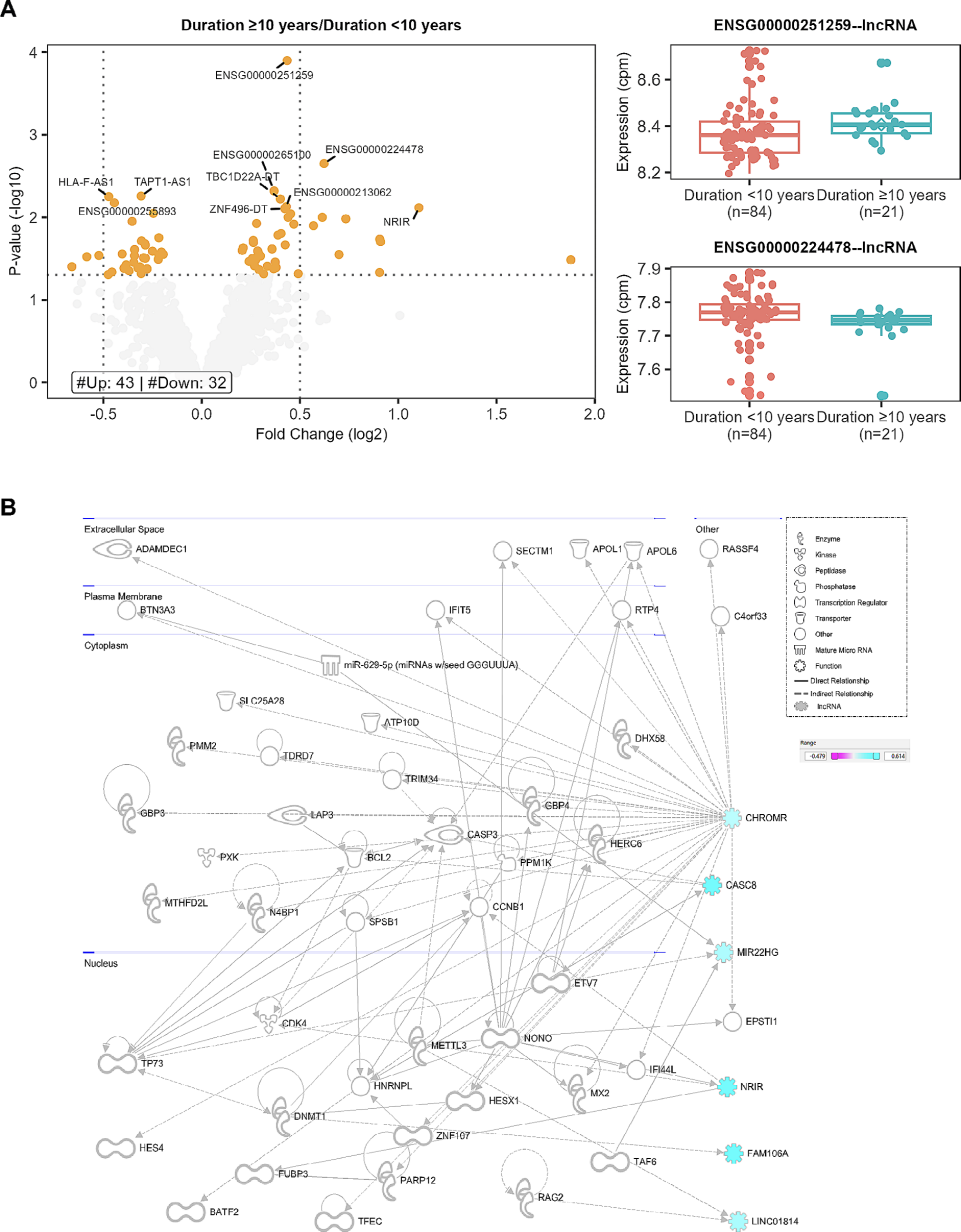
lncRNA Differential expression and pathway analysis of patients on diabetes duration. (**A**) Volcano plot and box plots showing the results of the differential expression analysis. The volcano plot shows significant genes at an FDR < 0.05 in red, significant genes at a p-value < 0.05 in orange, and non-significant genes in gray. The vertical dotted lines are set at abs(FC) = 0.5 and the horizontal dotted line at a -log10(0.05). The top 10 significant genes by p-value are labeled. The box plots show the expression states for the top 2 significant genes. (**B**) IPA network diagram of the merged networks in B. Gene colors indicate the logFC from our analysis as indicated in the legend

**Fig. 10:**
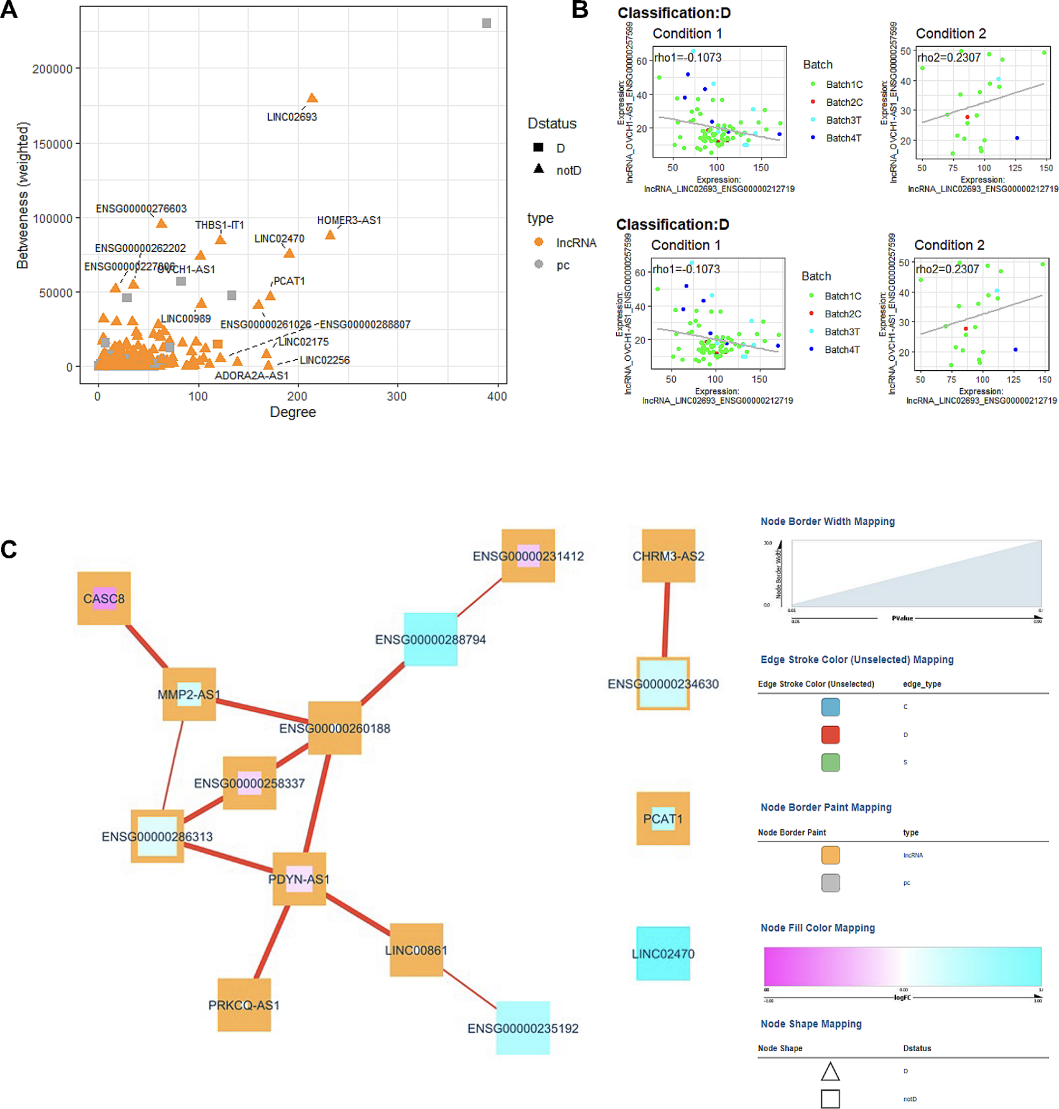
Diabetes duration co-expression analysis. (**A**) Scatter plot of Degree vs. Betweenness score for each gene in the diabetes-related network. The top 10 lncRNA genes by a degree or Betweenness score are labeled. (**B**) Correlation plots of two different gene pairs. As the legend indicates, each point is a sample belonging to one of four batches. The classification and regression coefficients (rho1, rho2) from the csdR results are displayed on the plot. (**C**) Visualization of the diabetes-related network shows the correlations between different genes. As indicated in the legend, node edges are proportional to the p-value, edge color corresponds to the C, D, and S types, node border color refers to lncRNA or pc genes, node interior color refers to the logFC, and node shape to lncRNA or protein-coding genes

**Fig. 11:**
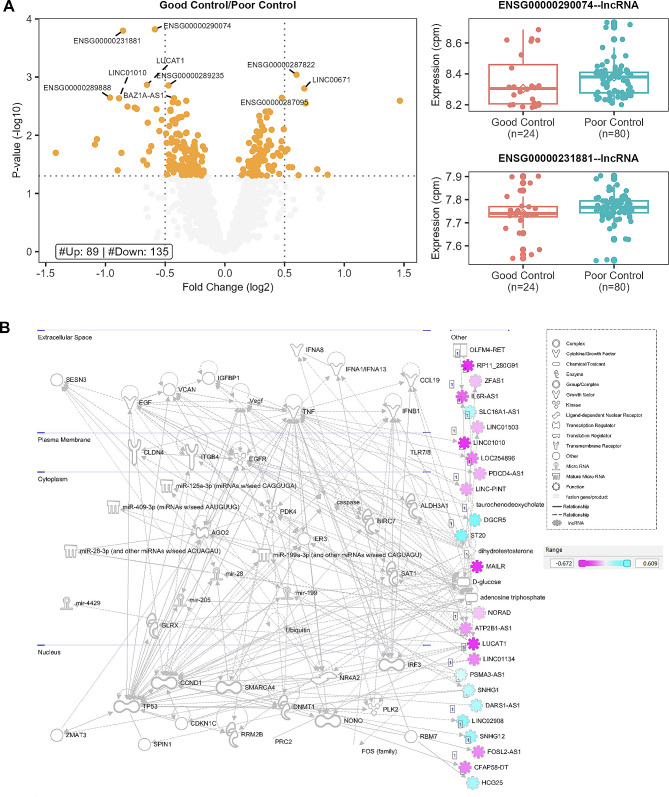
lncRNA Differential expression and pathway analysis of patients with good vs. poor control. (**A**) Volcano plot and box plots showing the results of the differential expression analysis. The volcano plot shows significant genes at an FDR < 0.05 in red, significant genes at a p-value < 0.05 in orange, and non-significant genes in gray. The top 10 significant genes by p-value are labeled. The box plots show the expression states for the top 2 significant genes. The vertical dotted lines are set at abs(FC) = 0.5 and the horizontal dotted line at a– log10(0.05). (**B**) IPA network diagram of the merged networks in B. Gene colors indicate the logFC from our analysis as indicated in the legend

Thirty patients with a diabetes duration < 10 years were compared to 90 patients with a diabetes duration ≥ of 10 years. Differential expression identified 41 upregulated and 32 downregulated lncRNA genes (Fig. 9A, Table S9). Pathway analysis (Fig. 9B, Table S10) identified networks of significant lncRNA genes (FAM106A, LINC01814, CASC8, MIR22HG, NRIR, and CHROMR) affecting neurological disease, cell cycle, ophthalmic disease, cardiovascular system, and metabolic disease. Expression correlation analysis also identified 15 diabetes-related lncRNA genes associated with known Diabetes genes (Fig. 10A). The highest degree lncRNA is ENSG00000269019 (HOMER3-AS10), while the lncRNA with the highest betweenness score is ENSG00000212719 (LINC02693, Fig. 11A). Two representative D correlations are shown in Fig. 10B. The core network is visualized in Fig. 10C, which shows only D correlations.

### Comparison of diabetes patients according to diabetes control

**Fig. 12:**
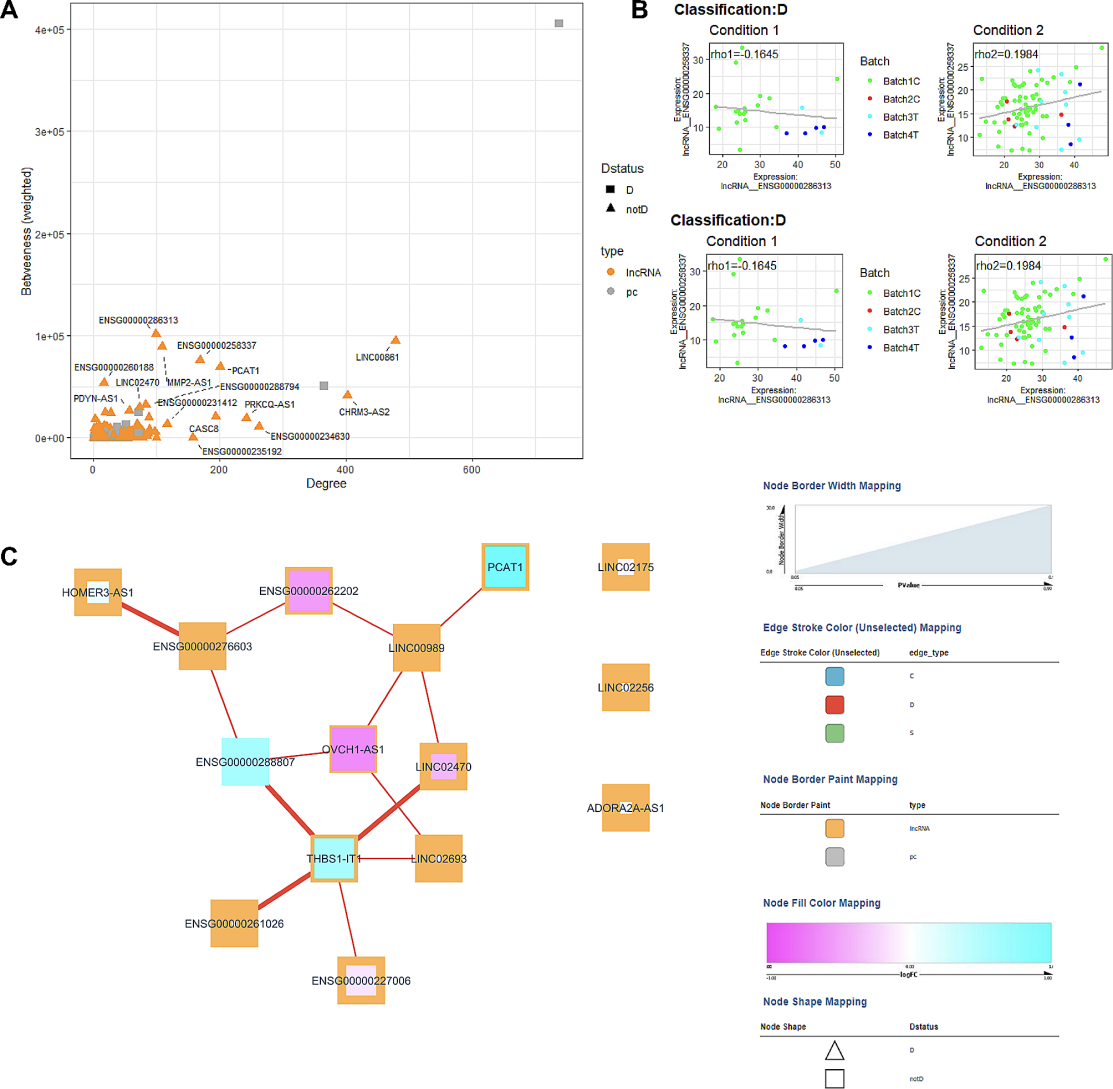
Diabetes control co-expression analysis. (**A**) Scatter plot of Degree vs. Betweenness score for each gene in the diabetes-related network. The top 10 lncRNA genes by a degree or Betweenness score are labeled. (**B**) Correlation plots of two different gene pairs. As the legend indicates, each point is a sample belonging to one of four batches. The classification and regression coefficients (rho1, rho2) from the csdR results are displayed on the plot. (**C**) Visualization of the diabetes-related network shows the correlations between different genes. As indicated in the legend, node edges are proportional to the p-value, edge color corresponds to the C, D, and S types, node border color refers to lncRNA or pc genes, node interior color refers to the logFC, and node shape to lncRNA or protein-coding genes

Twenty-four patients with an HbA1c < 7% were compared to 96 with an HbA1c ≥ 7%. Differential expression identified 102 upregulated and 104 downregulated lncRNA genes (Fig. 11A, Table S11). No significant genes with an FDR < 0.05 were identified. Pathway analysis (Fig. 11B, Table S12) identified networks of 15 downregulated significant lncRNA genes and upregulated lncRNA genes affecting cell death, cell development metabolism, and CVD. Expression correlation analysis also identified 15 lncRNA genes associated with known Diabetes genes (Fig. 12A). The highest degree lncRNA is ENSG00000245164 (LINC00861, Fig. 12A), while the highest lncRNA gene in-betweenness score is ENSG00000286313. Two representative correlation plots show D classification patterns in Fig. 12B. The network is visualized in Fig. 12C and is composed of D correlations.

### Aggregate analysis across all comparisons

**Fig. 13:**
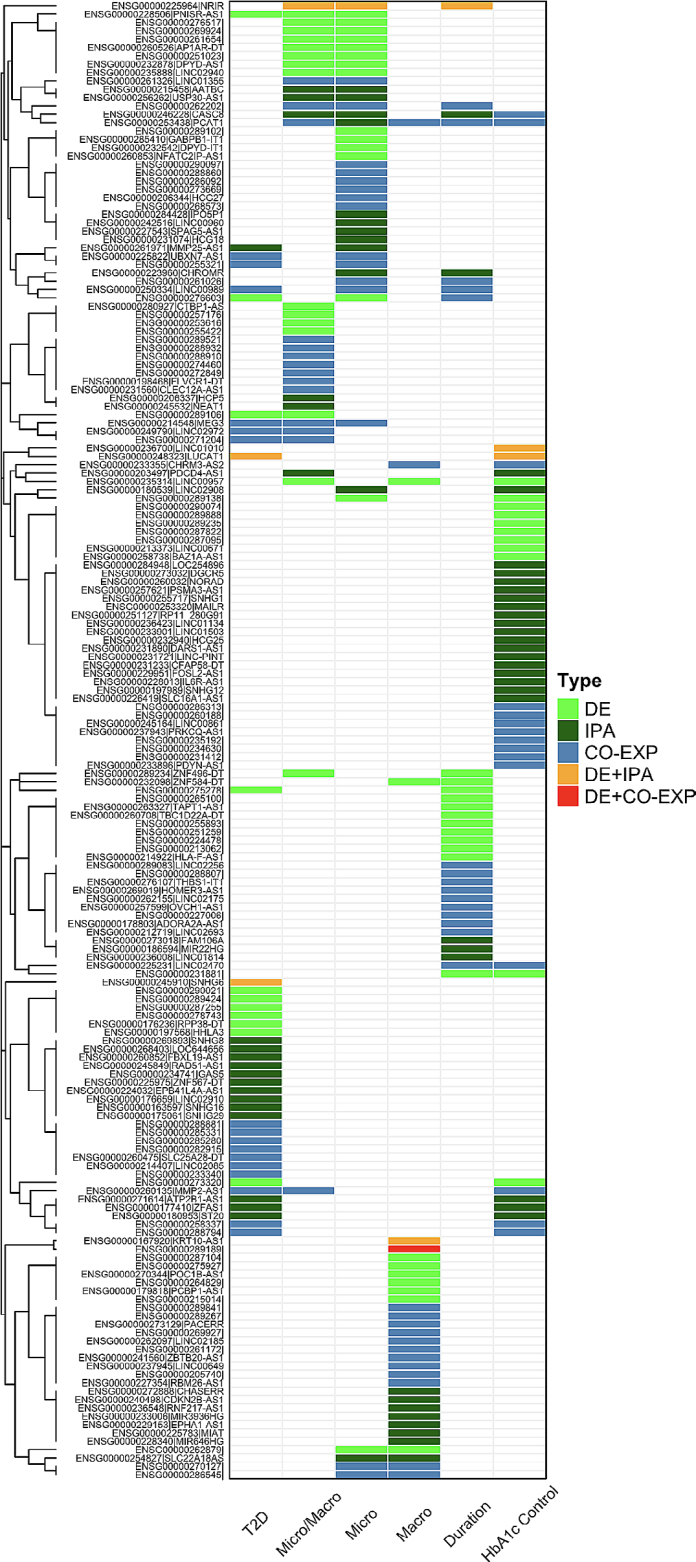
Clustergram aggregating the top 10 differentially expressed genes, all the pathway-associated lncRNAs, and the top differentially expressed genes for all comparisons. The color corresponds to the source of significance as indicated in the legend. DE: differential expression. IPA: pathway analysis. Co-exp: differential co-expression. DE + IPA: Significant in both differential expression and pathway analysis. DE + CO-EXP: Significant in both differential expression and co-expression analysis. The column labels correspond to the following comparisons: T2D: T2D vs. non-T2D, Micro: Microvascular only vs. no vascular disease, Macro: Macrovascular disease vs. no vascular disease, Micro/Macro: Micro and Macrovascular disease vs. no vascular disease. Duration: < 10 years, >= 10 years, HbA1c Control: Good vs. Poor control. The row labels are the Ensembl Gene Name and common gene name separated by “|”

To identify unique and shared lncRNA genes across the different comparisons, we carried out an aggregate analysis (Fig. 13) combining the top 10 significant genes by p-value (Figure S**2**), all the pathway-associated lncRNAs (Figure S3), and the top significant differentially co-expressed genes (Figure S4). Each comparison has many unique lncRNA genes not found in other comparisons. However, a few lncRNA genes are shared among different comparisons. PCAT1 is a significant gene in all five comparisons within diabetes patients. Similarly, CASC8 is a significant gene in all comparisons within diabetes patients except Macro. LncRNA genes appearing in three comparisons include NRIR, PNISR-AS1, ENSG00000262202, ENSG00000276603, LINC00989, LINC00957, MEG3, and MMP2-AS1.

## Discussion

Our analysis is among the largest RNA-sequencing-based studies focusing on lncRNA from human monocytes in diabetic patients with CVD, with our data coming from 120 type 2 diabetes patients and 37 non-diabetes controls. Previous monocyte studies from diabetes patients include a study published in 2021, which identified lncRNA DRAIR using RNA sequencing of monocytes isolated from 10 young participants [26]; a study in 2017 that reported the presence of new lncRNAs in human monocytes from 12 individuals [27] and another study that reported the presence of some dysregulated lncRNAs using transcriptome microarrays on whole blood samples collected from 12 samples [28]. Our large sample size and design, including a range of patients with different clinical phenotypes, enabled us to perform comparisons within diabetes patients with various vascular complications, diabetes duration, and HbA1c control. We utilized differential expression, pathway analysis, and co-expression analysis to identify novel roles for monocyte lncRNAs in diabetes and diabetes patients with vascular disease.

Differential expression analysis enables us to find highly significant genes in one group versus another one. Given the large number and heterogeneity of patients, we have adopted a statistically robust approach to identifying significant differentially expressed genes by performing covariate correction for the most impactful covariates as indicated in Table 1 and multiple testing correction. Using Ingenuity Pathway Analysis software, pathway analysis allows us to identify lncRNAs associated with known genes. Co-expression analysis enables the identification of correlation networks that differ between one group and another and the finding of associations between genes of unknown function and genes of known function [29–32]. We used two metrics of network significance – the degree and the maximum score weighted betweenness centrality. The degree is the number of connections a gene makes with another gene. The betweenness score represents the degree of importance for information flow within a network as measured by enumerating the nodes that connect the shortest path between all nodes in the network [33]. We weighted the betweenness by the csdR package maximum score of the correlation, which considers the variance and strength of the correlation. Thus, well-connected nodes with a high variance-adjusted correlation score have higher betweenness scores than well-connected genes with low correlation scores or more variable correlation scores. We used these co-expression analysis parameters to identify potentially new roles for lncRNAs of unknown functionality through their high association with known diabetes genes.

Within the comparison of T2D vs. non-T2D, we identified three significant lncRNAs with no previously reported diabetes-related function. The first is ENSG00000276603, which is lower in T2D than in non-T2D. The genomic localization of this gene is closest to BLCAP, but there are no publications on the specific role of this gene. Genes ENSG00000289424 and ENSG00000287255 are both upregulated in T2D patients with no function reports but with genomic localizations near protein-coding genes and within identified promoter regions. Co-expression analysis identified additional lncRNAs not previously associated with diabetes. We identified UBXN7-AS1 (ENSG00000225822) as the lncRNA gene with the highest betweenness score. UBXN7-AS1 is associated with UBXN7, a co-factor involved in ubiquitin ligases [34]. UBXN7 has been shown to be involved in regulating hypoxia-inducible factor-alpha and oxidative stress response [35–37]. Its high degree of correlation with the associated lncRNA is, therefore, consistent with the role of UBXN7. Interestingly, two recent reports of a circular RNA regulating UBXN7 demonstrate its role in macrophage-related diabetes kidney disease [38] and cell proliferation in A549 lung cancer cells [39]. No literature was found on the function of the highest degree lncRNA ENSG00000285280.

Within the vascular disease differential expression comparisons, we identified one lncRNA ENSG00000261654 (RP11_96K194), which is significantly upregulated in those with microvascular disease compared to those without vascular disease. The gene is also upregulated in patients with macro and microvascular disease. No promoter or enhancer regions overlap with this gene, but LRIF1 is a nearby gene. We have not found any functional studies for this lncRNA gene. Co-expression analysis of the vascular phenotypes also shows associations of lncRNAs with known diabetes genes and a highly connected lncRNA gene. The top degree lncRNA in comparing macro/micro and micro-vascular to patients without vascular disease is ENSG00000261326 (LINC01355, HYPAL). It shows differentiated correlations within the core diabetes-related network HYPAL, which is involved in the hypoxia response of bladder cancer cells [40]. The highest betweenness gene in patients with micro and macrovascular disease was ENSG00000260135 (MMP2-AS1). While no reports on MMP2-AS1 were found, MMP2 is a metalloprotease that has been implicated in many processes, including diabetic retinopathy [41], cardiac health [42, 43], and nephropathy [44]. ENSG00000286545, the highest betweenness lncRNA in the microvascular comparison, has no current reported function but is antisense to ZC3H8. ZC3H8 is highly expressed in different cell types and has been found to play a role in immune cell maturation [45]. In the macrovascular comparison, we also identified ENSG00000262097 and ENSG00000241560 (ZBTB20-AS1) as the top two highest lncRNA for betweenness and degree. While no reports on ENSG00000262097 were found, ZBTB20-AS1 has been show to regulate a transcription factor ZBTB20, which is involved in cancer [46], neural development [47–49], and macrophage-related atherosclerosis [50].

In comparing patients based on diabetes duration, we also identified two highly connected lncRNAs: ENSG00000269019 (HOMER3-AS10) and ENSG00000212719 (LINC02693). No specific reports about HOMER3-AS10 have been found, but HOMER3 plays a role in hypoxia [51] and dendritic cell glutamate signaling [52]. LINC02693 has been associated with binding to TRIM25, an RNA-binding E3 ubiquitin ligase protein involved in immune responses [53]. The comparison of patients with good vs. poor control showed the high-degree lncRNA gene ENSG00000245164 (LINC00861). LINC00861 has been associated with cervical cancer through the PTEN pathway [54] as well as associated with obesity in a GWAS [55]. ENSG00000286313 is an anti-sense transcript to PDE7B, a phosphodiesterase, which has been shown to play a role in keloid formation [56]. Interestingly, we found CASC8, a member of the core diabetes network, in the diabetes control comparison. CASC8 has been reported to be associated with cancer risk [57, 58], combined colorectal cancer and diabetes risk [59], cardiovascular risk [60], and role in glycolysis metabolism in bladder cancer cells [61]. Interestingly, we also find this gene to be differentially expressed with relatively high effect sizes in patients with micro and macrovascular disease (logFC =  – 1.96, p-value = 0.03) with microvascular disease only (logFC = – 2.47, p-value = 0.003) and with duration ≥ 10 years compared with duration < 10 years (log FC = 1.887, p-value = 0.03).

Several lncRNA genes appeared significantly across multiple conditions, as found by the aggregate analysis. These are PCAT1, CASC8, NRIR, PNISR-AS1, ENSG00000262202, ENSG00000276603, LINC00989, LINC00957, MEG3 and MMP2-AS1. PCAT1 was significant in all diabetes comparisons. PCAT1 has been extensively studied and its expression has been shown to promote proliferation in different cancers [62, 63]. However, the role of PCAT1 in monocyte function in diabetes is not currently known to our knowledge. Similarly, MEG3 has well-established functions in regulating differentiation and stemness [64] and has been found to be associated with vascular complications of diabetes [65]. NRIR is a functional lncRNA that has been shown to regulate interferon response [66] but has no established role in diabetes. LINC00989 was found in only one report to be associated with high myopia-related blindness in a Chinese population [67]. Similarly, LINC00957 was associated with chemoresistance in colorectal cancer [68]. No functional studies of PNISR-AS1 and ENSG00000262202 were found, and thus, they may be novel diabetes-associated genes. We have discussed earlier the genes CASC8, ENSG00000276603, and MMP2-AS1.

Overall, our analysis has uncovered several previously described lncRNA genes related to diabetes pathophysiology. Our pathway analysis results show several significant lncRNA genes previously identified in diabetes-related pathways. These genes include MEG3, NEAT1, NRIR, CHROMR, MIAT and LINC01814. Furthermore, pathway analysis showed that these genes interact directly or indirectly within highly significant functional networks. In addition to IPA results, our correlated expression analysis revealed dense networks of known diabetes-associated lncRNA and protein-coding genes in all comparisons. These highly associated known diabetes lncRNA genes include NEAT1, CDKN2B-AS1, MIAT, CHROMR, MEG3, CYTOR, PVT1, MALAT, and LINC01916 when combining all analyses. Thus, our differential expression and co-expression analysis identified many known diabetes-associated genes while potentially identifying new functional roles for several non-coding genes with unknown functions.

While our study includes a larger sample size and a diverse selection of patients with CVD compared to previous studies on monocyte lncRNA function, there are several limitations. Atherosclerosis is a systematic disease; hence, we cannot exclude non-documented atherosclerotic cardiovascular disease (ASCVD) in type 2 diabetes without macro or microvascular disease. For the same reason, we cannot exclude the presence of undocumented microvascular disease in patients with macrovascular disease only and the presence of macrovascular disease in patients with microvascular disease. In terms of methodology, as we used poly-A capture methods for RNA sequencing, we could only capture sample lncRNA with poly-A tails. This still includes many regulatory lncRNAs [69]. While the initial number of lncRNAs in our dataset with any read detected was 17,617, removing low read count genes resulted in a final lncRNA data set of 2084 genes. This is compared to protein-coding genes, where low read filtration resulted in 11,248 genes out of an initial 19,122 genes. Second, some comparisons for patients within the diabetes group are underpowered. This is especially the case when comparing patients with only macrovascular disease, as most patients had concomitant microangiopathy. Co-expression correlation analysis also benefits from large sample sizes as results are more robust against random correlations with large sample sizes. Third, we have also not analyzed sex differences. Analyzing the effects of sex chromosome genes would require doubling the current sample size to maintain the same power. However, this important aspect should be explored in future studies with higher sample sizes. Finally, we have identified several lncRNAs with no known function. While these unknown function genes often overlap with known enhancer and promoter sites or are adjacent to protein-coding genes within a genomic context, we cannot assign any functional role at this stage. Future work will include a more in-depth investigation of possible functional roles in those genes with no known function.

Exploring lncRNAs in cardiovascular disease and diabetes holds promise for unveiling novel therapeutic targets and diagnostic biomarkers. Understanding the intricate web of interactions involving lncRNAs could provide insights into the molecular underpinnings of these complex diseases, paving the way for developing more effective and personalized treatment strategies. As research advances, the role of long non-coding RNAs in cardiovascular disease, particularly in the context of diabetes, is likely to become increasingly central to our understanding of disease pathogenesis and progression.

## Conclusion

In this exploratory study using a diverse patient cohort of 157 individuals, we have found several novel lncRNAs associated with type 2 diabetes and its complications. We have also found several known genes associated with cancer and viral response that may have a new role in monocytes of diabetes patients, potentially involved in the pathology of vascular complications. Future work will focus on deeper understanding and validation of these dysregulated genes and pathways.

### Electronic supplementary material

Below is the link to the electronic supplementary material.

Supplementary Material 1

## Data Availability

No datasets were generated or analysed during the current study.

## Abbreviations

CVD: Cardiovascular disease
lncRNAs: Long non-coding RNAs
PC: protein-coding
RNA seq: RNA sequencing
T2D: Type 2 diabetes
