# Supplementary material for: Dysregulation of long non-coding RNA gene expression pathways in monocytes of type 2 diabetes patients with cardiovascular disease
Source: Cardiovasc Diabetol. 2024 Jun 7;23:196. doi: 10.1186/s12933-024-02292-1 (PMC11161966; doi:10.1186/s12933-024-02292-1)
Supplement: Supplementary file 1 — Supplementary Material 1 [file 12933_2024_2292_MOESM1_ESM.docx]

# Supplementary File:

**Figure S1**. Batch correction multidimensional scaling plots showing the effect of batch correction on sample gene expression changes on the first and second dimensions. Each point is a sample, with the color representing the batch and the shape representing whether the sample is from a T2D patient vs no diabetes.

**Table S1:** References used for identifying diabetes-related long non-coding RNAs.

| **#** | **Title** | **Year** | **Type** | **Reference** |
| --- | --- | --- | --- | --- |
| 1 | Proteomic profiling of high glucose primed monocytes identifies cyclophilin A as a potential secretory marker of inflammation in type 2 diabetes | 2012 | Research | [1] |
| 2 | RNA-sequencing analysis of high glucose-treated monocytes reveals novel transcriptome signatures and associated epigenetic profiles | 2013 | Research | [2] |
| 3 | Regulation of Inflammatory Phenotype in Macrophages by a Diabetes-Induced Long Noncoding RNA | 2014 | Research | [3] |
| 4 | Aberrant Expression of Long Non-Coding RNAs in Newly Diagnosed Type 2 Diabetes Indicates Potential Roles in Chronic Inflammation and Insulin Resistance | 2017 | Research | [4] |
| 5 | Deep RNA Sequencing Uncovers a Repertoire of Human Macrophage Long Intergenic Noncoding RNAs Modulated by Macrophage Activation and Associated With Cardiometabolic Diseases | 2017 | Research | [5] |
| 6 | Effect of High Glucose on Cytokine production by Human Peripheral Blood Immune Cells and Type I Interferon Signaling in Monocytes: Implications for the Role of Hyperglycemia in the Diabetes Inflammatory Process and Host Defense against Infection | 2018 | Research | [6] |
| 7 | Hematopoietic Deficiency of the Long Noncoding RNA MALAT1 Promotes Atherosclerosis and Plaque Inflammation | 2018 | Research | [7] |
| 8 | LncRNA uc.48+ is involved in the diabetic immune and inflammatory responses mediated by P2X7 receptor in RAW264.7 macrophages | 2018 | Research | [8] |
| 9 | Long Noncoding RNAs in Diabetes and Diabetic Complications | 2018 | Review | [9] |
| 10 | Transcriptional regulation of macrophage cholesterol efflux and atherogenesis by a long noncoding RNA | 2018 | Research | [10] |
| 11 | Linking diabetic vascular complications with LncRNAs | 2019 | Review | [11] |
| 12 | Integrative Omics Analyses Reveal Epigenetic Memory in Diabetic Renal Cells Regulating Genes Associated With Kidney Dysfunction | 2020 | Research | [12] |
| 13 | Role of epigenetic mechanisms regulated by enhancers and long noncoding RNAs in cardiovascular disease | 2020 | Review | [13] |
| 14 | Emerging Role of Long Non-Coding RNAs in Diabetic Vascular Complications | 2021 | Review | [14] |
| 15 | Epigenetic Mechanisms in Diabetic Vascular Complications and Metabolic Memory: The 2020 Edwin Bierman Award Lecture | 2021 | Review | [15] |
| 16 | lncRNA DRAIR is downregulated in diabetic monocytes and modulates the inflammatory phenotype via epigenetic mechanisms | 2021 | Research | [16] |
| 17 | Non-Coding RNA and Diabetic Kidney Disease | 2021 | Review | [17] |
| 18 | The Impact of lncRNAs in Diabetes Mellitus: A Systematic Review and In Silico Analyses | 2021 | Review | [18] |
| 19 | Epigenetic modifications in metabolic memory: What are the memories, and can we erase them? | 2022 | Review | [19] |
| 20 | Monocyte miRNAs Are Associated With Type 2 Diabetes | 2022 | Research | [20] |
| 21 | Potential value of lncRNAs as a biomarker for proliferative diabetic retinopathy | 2022 | Research | [21] |

**Table S2:** Correspondence between Ensembl IDS and Common Names for Diabetes-related lncRNAs. A blank in the Ensembl ID column indicates that no human corresponding Ensembl ID was found for the gene name mentioned in the literature. The note column mentions specific issues encountered in identifying the Ensembl ID of a specific gene.

| **ENSEMBL ID** | **Gene name IN Literature** | **Note** |
| --- | --- | --- |
| ENSG00000240498 | ANRIL |  |
| ENSG00000130600 | H19 |  |
| ENSG00000228630 | HOTAIR |  |
|  | rEPa | mouse specific/NAME CHANGED? |
| ENSG00000258492 | Kcnq1ot1 |  |
| ENSG00000245532 | NEAT1 |  |
| ENSG00000251562 | MALAT1 |  |
| ENSG00000259577 | LOC100129973 |  |
| ENSG00000222041 | Linc00152 |  |
| ENSG00000255737 | PUNISHER |  |
| ENSG00000254703 | SENCR |  |
| ENSG00000269902 | Lnc-Ang362 |  |
| ENSG00000254303 | SMILR |  |
| ENSG00000262454 | hyperlnc | MIR193b-365a host gene |
| ENSG00000225783 | lnc-MIAT |  |
| ENSG00000214548 | MEG3 |  |
| ENSG00000249669 | E330013P06 | CARMEN, MIR143HG synonyms? |
|  | Lethe | ENSMUSG00000083757, mouse, no orthologues |
| ENSG00000249859 | Pvt1 |  |
| ENSG00000225746 | Lnc-MGC |  |
| ENSG00000253352 | Tug1 |  |
| ENSG00000232679 | RP11-472 N13.3 | closest match, RP11-400N13.3 |
|  | Giver | identified in Rat, not in Ensembl |
| ENSG00000230630 | Dnm3Os |  |
|  | Mist | identified in MICE, XLOC_019580 |
| ENSG00000266010 | GATA6-AS |  |
|  | MeXis | ENSMUSG00000086712, mouse, no human orthologues in Ensembl |
| ENSG00000223960 | CHROME |  |
| ENSG00000232855 | AF131217.1 |  |
| ENSG00000258791 | LEENE |  |
|  | NTT |  |
| ENSG00000247624 | DRAIR |  |
|  | LncRNA-CCL2 |  |
| ENSG00000224189 | STEEL | HAGLR, HOXD-AS1, closest match |
| ENSG00000235927 | NEXN-AS1 |  |
| ENSG00000288869 | MANTIS |  |
| ENSG00000245694 | CRNDE |  |
|  | LIPCAR | JA760602 (uc022bqs.1), not in Ensembl, mitochondrial |
| ENSG00000288944 | CHAER |  |
| ENSG00000225746 | LRNA9884 | ENSG00000225746,gene cards match?AL132709.8, BSR, IRM, LINC00024, LNC-MGC, NCRNA00024, RIAN, SNHG23, SNHG24 |
| ENSG00000276365 | Erbb4-IR | ENSG00000276365,genecards match? HSA-MIR-145, MIR-145, MIRN145 |
|  | Linc-YY1 |  |
|  | Linc-Ram |  |
|  | Dum |  |
|  | LncSTR |  |
|  | SRA |  |
|  | HI-LNC25 |  |
|  | Blinc1 |  |
| ENSG00000249673 | ENST00000505731 |  |
|  | NR-126161 |  |
|  | Cj241444 |  |
| ENSG00000257242 | LINC01619 |  |
|  | Gm6135 | ENSMUSG00000106069, no primate orthologues |
|  | 150Rik |  |
|  | 1700020I14Rik | mouse, RIKEN cDNA 1700020I14 gene [Source:MGI Symbol;Acc:MGI:1913852] |
|  | PVT1 |  |
|  | Gm4419 | ENSMUSG00000097055, no primate orthologues |
| ENSG00000246430 | Linc00968 |  |
| ENSG00000277209 | Rpph1 |  |
| ENSG00000237036 | ZEB1-AS |  |
|  | RP23– 298H6. 1–001 |  |
|  | CASC2 |  |
|  | GM5524 | ENSMUSG00000101514, no primate orthologues |
|  | GM15645 | ENSMUSG00000086414 |
| ENSG00000234741 | GAS5 |  |
|  | uc.48+ |  |
| ENSG00000257510 | ENST00000550337.1 |  |
|  | Pluto |  |
|  | LncRNAp3134 |  |
|  | n335556 |  |
|  | n336109 |  |
|  | n342533 |  |

| **Table S3**: All significant genes (p-value < 0.05) for the “T2D vs. non-T2D” comparison with the ENSEMBL ID and the IPA-identified gene SYMBOL | | | | | | |
| --- | --- | --- | --- | --- | --- | --- |
| Expr Log Ratio | Expr p-value | Expr False Discovery Rate (q-value) | ID | Flags | Symbol | Entrez Gene Name |
| -2.072 | 9.1E-06 | 0.0172 | ENSG00000276603 |  | RP11_425M57 |  |
| 0.457 | 1.7E-05 | 0.0172 | ENSG00000289424 |  | ENSG00000289424 |  |
| 0.467 | 7E-05 | 0.0484 | ENSG00000287255 |  | LOC101929667 |  |
| 0.671 | 0.00098 | 0.399 | ENSG00000176236 |  | RPP38-DT | RPP38 divergent transcript |
| -0.264 | 0.00161 | 0.399 | ENSG00000245910 |  | SNHG6 | small nucleolar RNA host gene 6 |
| 0.427 | 0.00174 | 0.399 | ENSG00000273320 |  | RP11_22N192 |  |
| 0.312 | 0.0022 | 0.399 | ENSG00000290021 |  | ENSG00000290021 |  |
| -0.477 | 0.00221 | 0.399 | ENSG00000197568 |  | ANKRD13C-DT | ANKRD13C divergent transcript |
| 0.499 | 0.00238 | 0.399 | ENSG00000275278 |  | RP11_946L162 |  |
| 0.386 | 0.00264 | 0.399 | ENSG00000278743 |  | RP11_707G181 |  |
| 0.41 | 0.0032 | 0.399 | ENSG00000257058 |  | RP11_864I44 |  |
| 0.478 | 0.0034 | 0.399 | ENSG00000242474 |  | LINC03015 | long intergenic non-protein coding RNA 3015 |
| 0.284 | 0.00351 | 0.399 | ENSG00000278600 |  | RP11_81A16 |  |
| -0.231 | 0.00355 | 0.399 | ENSG00000224186 |  | PITX1-AS1 | PITX1 antisense RNA 1 |
| 0.487 | 0.00362 | 0.399 | ENSG00000228113 |  | AC0039913 |  |
| -0.214 | 0.00365 | 0.399 | ENSG00000267787 |  | RP11_35G95 |  |
| 0.352 | 0.00372 | 0.399 | ENSG00000289331 |  | ENSG00000289331 |  |
| -0.254 | 0.00387 | 0.399 | ENSG00000175061 |  | SNHG29 | small nucleolar RNA host gene 29 |
| 0.433 | 0.00393 | 0.399 | ENSG00000289865 |  | ENSG00000289865 |  |
| 0.23 | 0.00407 | 0.399 | ENSG00000248489 |  | CHD1-DT |  |
| 0.339 | 0.00439 | 0.399 | ENSG00000235527 |  | HIPK1-AS1 | HIPK1 antisense RNA 1 |
| -0.185 | 0.00448 | 0.399 | ENSG00000234741 |  | GAS5 | growth arrest specific 5 |
| 0.351 | 0.00448 | 0.399 | ENSG00000228323 |  | MYADM-AS1 |  |
| 0.317 | 0.00463 | 0.399 | ENSG00000260742 |  | ITPRID2-DT | ITPRID2 divergent transcript |
| 0.367 | 0.00499 | 0.401 | ENSG00000260528 |  | FAM157C | family with sequence similarity 157 member C |
| 0.41 | 0.00505 | 0.401 | ENSG00000267262 |  | CTC_232P53 |  |
| 0.31 | 0.00604 | 0.458 | ENSG00000289410 |  | ENSG00000289410 |  |
| 0.186 | 0.00625 | 0.458 | ENSG00000241163 |  | LINC00877 |  |
| -0.232 | 0.00641 | 0.458 | ENSG00000288771 |  | ENSG00000288771 |  |
| 0.568 | 0.00695 | 0.48 | ENSG00000233896 |  | PDYN-AS1 | PDYN antisense RNA 1 |
| -0.133 | 0.00745 | 0.497 | ENSG00000249673 |  | NOP14-AS1 | NOP14 antisense RNA 1 |
| -0.367 | 0.00782 | 0.505 | ENSG00000229671 |  | LINC01150 | long intergenic non-protein coding RNA 1150 |
| -0.227 | 0.00896 | 0.558 | ENSG00000231312 |  | MAP4K3-DT | MAP4K3 divergent transcript |
| -0.238 | 0.00934 | 0.558 | ENSG00000176659 |  | LINC02910 | long intergenic non-protein coding RNA 2910 |
| -0.289 | 0.00969 | 0.558 | ENSG00000258376 |  | PAPLN-AS1 | PAPLN antisense RNA 1 |
| 0.401 | 0.00982 | 0.558 | ENSG00000290041 |  | ENSG00000290041 |  |
| -0.307 | 0.0102 | 0.558 | ENSG00000289106 |  | ENSG00000289106 |  |
| -0.181 | 0.0102 | 0.558 | ENSG00000224032 |  | EPB41L4A-AS1 | EPB41L4A antisense RNA 1 |
| -0.558 | 0.0105 | 0.558 | ENSG00000250155 |  | SLC1A3-AS1 |  |
| -0.224 | 0.0111 | 0.565 | ENSG00000261455 |  | LINC01003 | long intergenic non-protein coding RNA 1003 |
| 0.27 | 0.0114 | 0.565 | ENSG00000268403 |  | LOC644656 | uncharacterized LOC644656 |
| 0.308 | 0.0115 | 0.565 | ENSG00000282508 |  | LINC01002 | long intergenic non-protein coding RNA 1002 |
| 0.363 | 0.0118 | 0.568 | ENSG00000282572 |  | FAM157D |  |
| 0.598 | 0.0121 | 0.568 | ENSG00000288879 |  | ENSG00000288879 |  |
| 0.383 | 0.0127 | 0.575 | ENSG00000289005 |  | ENSG00000289005 |  |
| -0.186 | 0.0133 | 0.575 | ENSG00000237476 |  | LINC01637 | long intergenic non-protein coding RNA 1637 |
| 0.223 | 0.0134 | 0.575 | ENSG00000259642 |  | ST20-AS1 | ST20 antisense RNA 1 |
| 0.217 | 0.0137 | 0.575 | ENSG00000261971 |  | MMP25-AS1 | MMP25 antisense RNA 1 |
| -0.285 | 0.0145 | 0.575 | ENSG00000277851 |  | LINC02391 |  |
| -0.239 | 0.0147 | 0.575 | ENSG00000163597 |  | SNHG16 | small nucleolar RNA host gene 16 |
| 0.279 | 0.0148 | 0.575 | ENSG00000273218 |  | LLNLR_246C61 |  |
| 0.407 | 0.0153 | 0.575 | ENSG00000286116 |  | AL157394.2 |  |
| -0.321 | 0.0153 | 0.575 | ENSG00000273314 |  | RP5_1136G132 |  |
| 0.284 | 0.0157 | 0.575 | ENSG00000289626 |  | ENSG00000289626 |  |
| 0.485 | 0.0157 | 0.575 | ENSG00000180953 |  | ST20 | suppressor of tumorigenicity 20 |
| -0.215 | 0.0158 | 0.575 | ENSG00000280734 |  | LINC01232 | long intergenic non-protein coding RNA 1232 |
| 0.348 | 0.0166 | 0.575 | ENSG00000288748 |  | ENSG00000288748 |  |
| 0.312 | 0.017 | 0.575 | ENSG00000242628 |  | AC0092281 |  |
| -0.262 | 0.017 | 0.575 | ENSG00000223478 |  | ZDHHC12-DT | ZDHHC12 divergent transcript |
| -0.247 | 0.0175 | 0.575 | ENSG00000288924 |  | ENSG00000288924 |  |
| 0.223 | 0.0177 | 0.575 | ENSG00000289155 |  | ENSG00000289155 |  |
| -0.215 | 0.0186 | 0.575 | ENSG00000182257 |  | PRR34 | PRR34 long non-coding RNA |
| 0.238 | 0.0188 | 0.575 | ENSG00000286067 |  | AC004263.2 |  |
| 0.318 | 0.019 | 0.575 | ENSG00000224977 |  | RC3H1-DT |  |
| -0.189 | 0.0193 | 0.575 | ENSG00000196204 |  | RNF216P1 | ring finger protein 216 pseudogene 1 |
| 0.266 | 0.0194 | 0.575 | ENSG00000260852 |  | FBXL19-AS1 | FBXL19 antisense RNA 1 |
| -0.273 | 0.0198 | 0.575 | ENSG00000271964 |  | RP11_415F232 |  |
| -0.454 | 0.0201 | 0.575 | ENSG00000260188 |  | CTA_331P31 |  |
| 0.295 | 0.0202 | 0.575 | ENSG00000289142 |  | ENSG00000289142 |  |
| 0.255 | 0.0204 | 0.575 | ENSG00000288919 |  | ENSG00000288919 |  |
| -0.258 | 0.0204 | 0.575 | ENSG00000267216 |  | ZNF8-ERVK3-1 | ZNF8-ERVK3-1 readthrough (NMD candidate) |
| -0.218 | 0.0206 | 0.575 | ENSG00000262468 |  | LINC01569 |  |
| -0.181 | 0.0206 | 0.575 | ENSG00000269893 |  | SNHG8 | small nucleolar RNA host gene 8 |
| 0.351 | 0.0209 | 0.575 | ENSG00000289928 |  | ENSG00000289928 |  |
| -0.177 | 0.0211 | 0.575 | ENSG00000245904 |  | BTG1-DT |  |
| 0.245 | 0.0211 | 0.575 | ENSG00000225484 |  | NUTM2B-AS1 | NUTM2B antisense RNA 1 |
| 0.316 | 0.022 | 0.58 | ENSG00000255443 |  | CD44-AS1 | CD44 antisense RNA 1 |
| 0.215 | 0.022 | 0.58 | ENSG00000276900 |  | RP11_467L137 |  |
| -0.24 | 0.0224 | 0.58 | ENSG00000285728 |  | AC098484.4 |  |
| 0.252 | 0.0228 | 0.58 | ENSG00000205885 |  | C1RL-AS1 | C1RL antisense RNA 1 |
| 0.356 | 0.0229 | 0.58 | ENSG00000248323 |  | LUCAT1 | lung cancer associated transcript 1 |
| 0.225 | 0.023 | 0.58 | ENSG00000290057 |  | ENSG00000290057 |  |
| 0.288 | 0.0239 | 0.583 | ENSG00000238160 |  | LINC02863 |  |
| 0.467 | 0.024 | 0.583 | ENSG00000288818 |  | ENSG00000288818 |  |
| -0.309 | 0.0243 | 0.583 | ENSG00000234650 |  | PCCA-AS1 | PCCA antisense RNA 1 |
| 0.727 | 0.0243 | 0.583 | ENSG00000259398 |  | RP11_430B11 |  |
| -0.152 | 0.0245 | 0.583 | ENSG00000259953 |  | LINC02977 |  |
| 0.315 | 0.0258 | 0.607 | ENSG00000278869 |  | CITF22_49E93 |  |
| 0.324 | 0.0265 | 0.617 | ENSG00000267940 |  | RP11_290F246 |  |
| 0.293 | 0.0269 | 0.618 | ENSG00000289883 |  | ENSG00000289883 |  |
| 0.24 | 0.0272 | 0.618 | ENSG00000261592 |  | RP11_178L83 |  |
| 0.238 | 0.0282 | 0.634 | ENSG00000260233 |  | ZNRD2-DT | ZNRD2 divergent transcript |
| -0.205 | 0.0294 | 0.646 | ENSG00000272711 |  | HK2-DT | HK2 divergent transcript |
| -0.603 | 0.0297 | 0.646 | ENSG00000256948 |  | IQSEC3-AS2 |  |
| 0.379 | 0.0302 | 0.646 | ENSG00000286581 |  | AL450992.3 |  |
| 0.179 | 0.0308 | 0.646 | ENSG00000245849 |  | RAD51-AS1 | RAD51 antisense RNA 1 |
| -0.174 | 0.031 | 0.646 | ENSG00000247121 |  | CTD_2260A172 |  |
| -0.179 | 0.031 | 0.646 | ENSG00000289977 |  | ENSG00000289977 |  |
| 0.16 | 0.0313 | 0.646 | ENSG00000215068 |  | ANXA2R-AS1 |  |
| 0.483 | 0.0314 | 0.646 | ENSG00000230923 |  | LINC00309 | long intergenic non-protein coding RNA 309 |
| -0.183 | 0.0318 | 0.646 | ENSG00000282851 |  | BISPR | BST2 interferon stimulated positive regulator |
| 0.261 | 0.0319 | 0.646 | ENSG00000267737 |  | AC0619922 |  |
| -0.224 | 0.0334 | 0.664 | ENSG00000230424 |  | EMC1-AS1 |  |
| -0.216 | 0.0334 | 0.664 | ENSG00000228506 |  | PNISR-AS1 |  |
| 0.21 | 0.0342 | 0.664 | ENSG00000232104 |  | RFX3-DT |  |
| 0.164 | 0.0345 | 0.664 | ENSG00000230113 |  | AC0911771 |  |
| -0.168 | 0.0347 | 0.664 | ENSG00000285554 |  | LINC02988 | long intergenic non-protein coding RNA 2988 |
| -0.318 | 0.0351 | 0.664 | ENSG00000230013 |  | CT70 |  |
| 0.208 | 0.0353 | 0.664 | ENSG00000289564 |  | ENSG00000289564 |  |
| 0.243 | 0.0359 | 0.664 | ENSG00000289206 |  | ENSG00000289206 |  |
| 0.302 | 0.0362 | 0.664 | ENSG00000185065 |  | UFD1-AS1 |  |
| -0.227 | 0.0363 | 0.664 | ENSG00000289294 |  | ENSG00000289294 |  |
| 0.253 | 0.037 | 0.664 | ENSG00000277767 |  | RP11_365P135 |  |
| 0.254 | 0.0375 | 0.664 | ENSG00000236194 |  | AC0031041 |  |
| -0.289 | 0.0379 | 0.664 | ENSG00000223552 |  | CCR5AS |  |
| -0.213 | 0.0381 | 0.664 | ENSG00000225975 |  | ZNF567-DT | ZNF567 divergent transcript |
| -0.371 | 0.0384 | 0.664 | ENSG00000232229 |  | LINC00865 |  |
| 0.242 | 0.0389 | 0.664 | ENSG00000273855 |  | RP11_133K112 |  |
| -0.406 | 0.0389 | 0.664 | ENSG00000226334 |  | RP11_217B72 |  |
| 0.372 | 0.0389 | 0.664 | ENSG00000225138 |  | SLC9A3-AS1 | SLC9A3 antisense RNA 1 |
| -0.188 | 0.039 | 0.664 | ENSG00000245864 |  | MEF2C-AS2 |  |
| 0.221 | 0.0394 | 0.664 | ENSG00000273141 |  | RP11_820I164 |  |
| -0.259 | 0.0399 | 0.664 | ENSG00000267136 |  | RP11_53B23 |  |
| 0.366 | 0.0406 | 0.664 | ENSG00000287367 |  | LOC105378969 |  |
| -0.378 | 0.0408 | 0.664 | ENSG00000260943 |  | LINC02555 | long intergenic non-protein coding RNA 2555 |
| 0.304 | 0.0408 | 0.664 | ENSG00000283959 |  | LOC101927245 |  |
| -0.154 | 0.0413 | 0.664 | ENSG00000288156 |  | AC104530.1 |  |
| -0.225 | 0.0419 | 0.664 | ENSG00000228172 |  | RP1_317E233 |  |
| -0.413 | 0.0422 | 0.664 | ENSG00000269066 |  | CTD_2528A145 |  |
| -0.645 | 0.0423 | 0.664 | ENSG00000231560 |  | CLEC12A-AS1 | CLEC12A antisense RNA 1 |
| 0.234 | 0.0423 | 0.664 | ENSG00000273004 |  | GS1_279B72 |  |
| 0.218 | 0.0424 | 0.664 | ENSG00000271614 |  | ATP2B1-AS1 | ATP2B1 antisense RNA 1 |
| -0.269 | 0.0428 | 0.664 | ENSG00000235908 |  | RHOA-IT1 |  |
| -0.195 | 0.0435 | 0.664 | ENSG00000286388 |  | AC026748.3 |  |
| -0.191 | 0.0435 | 0.664 | ENSG00000273271 |  | AP0002548 |  |
| 0.265 | 0.0439 | 0.664 | ENSG00000275854 |  | RP11_278C75 |  |
| 0.156 | 0.0442 | 0.664 | ENSG00000267394 | D | ATXN7L3-AS1 |  |
| -0.384 | 0.0443 | 0.664 | ENSG00000238042 |  | LINC02257 | long intergenic non-protein coding RNA 2257 |
| 0.286 | 0.0452 | 0.669 | ENSG00000290044 |  | ENSG00000290044 |  |
| -0.209 | 0.0452 | 0.669 | ENSG00000284882 |  | LOC105378819 |  |
| 0.23 | 0.0457 | 0.67 | ENSG00000245112 |  | SMARCA5-AS1 | SMARCA5 antisense RNA 1 |
| 0.219 | 0.0466 | 0.68 | ENSG00000285852 |  | LOC124902388 | uncharacterized LOC124902388 |
| -0.13 | 0.0471 | 0.681 | ENSG00000177410 |  | ZFAS1 | ZNFX1 antisense RNA 1 |
| -0.23 | 0.0478 | 0.684 | ENSG00000223891 |  | OSER1-DT | OSER1 divergent transcript |
| -0.191 | 0.0479 | 0.684 | ENSG00000228801 |  | PCMTD1-DT |  |
| 0.496 | 0.0491 | 0.688 | ENSG00000178803 |  | ADORA2A-AS1 | ADORA2A antisense RNA 1 |
| 0.261 | 0.0492 | 0.688 | ENSG00000289576 |  | ENSG00000289576 |  |
| 0.22 | 0.0496 | 0.688 | ENSG00000235488 |  | JARID2-AS1 | JARID2 antisense RNA 1 |

**Table S4:** All significant networks identified by IPA for T2D vs non-T2D participants.

**
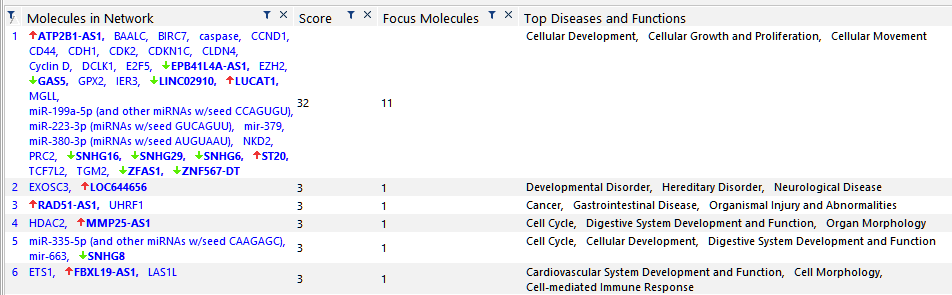
**

**Table S5:** All significant genes (p-value < 0.05) for the “Macro|Micro vs. no vascular disease” comparison with the ENSEMBL ID and the IPA-identified gene SYMBOL.

| Expr Log Ratio | Expr p-value | Expr False Discovery Rate (q-value) | ID | Flags | Symbol | Entrez Gene Name |
| --- | --- | --- | --- | --- | --- | --- |
| -1.488 | 0.  00108 | 0.993 | ENSG00000225964 |  | NRIR | negative regulator of interferon response |
| 0.451 | 0.00454 | 0.993 | ENSG00000276517 |  | AL1332432 |  |
| -0.603 | 0.00614 | 0.993 | ENSG00000253616 |  | RP11_875O113 |  |
| 0.391 | 0.00663 | 0.993 | ENSG00000251023 |  | RP11_549J181 |  |
| 0.407 | 0.0067 | 0.993 | ENSG00000280927 |  | CTBP1-AS | CTBP1 antisense RNA |
| -1.044 | 0.00841 | 0.993 | ENSG00000255422 |  | LOC105369519 |  |
| 0.507 | 0.00867 | 0.993 | ENSG00000257176 |  | LOC100506606 | uncharacterized LOC100506606 |
| 0.634 | 0.00973 | 0.993 | ENSG00000232878 |  | DPYD-AS1 | DPYD antisense RNA 1 |
| -0.47 | 0.00973 | 0.993 | ENSG00000289106 |  | ENSG00000289106 |  |
| 0.458 | 0.0107 | 0.993 | ENSG00000269924 |  | RP11_697N184 |  |
| 0.574 | 0.0111 | 0.993 | ENSG00000273129 |  | PACERR |  |
| 0.493 | 0.0131 | 0.993 | ENSG00000197301 |  | HMGA2-AS1 | HMGA2 antisense RNA 1 |
| -0.305 | 0.0134 | 0.993 | ENSG00000284669 |  | AC092053.3 |  |
| 0.466 | 0.0136 | 0.993 | ENSG00000257497 |  | GLIPR1-AS1 | GLIPR1 antisense RNA 1 |
| -0.45 | 0.0137 | 0.993 | ENSG00000289234 |  | ZNF496-DT |  |
| 0.373 | 0.0139 | 0.993 | ENSG00000260526 |  | AP1AR-DT | AP1AR divergent transcript |
| 0.56 | 0.0142 | 0.993 | ENSG00000288025 |  | LOC112268474 |  |
| 0.358 | 0.015 | 0.993 | ENSG00000224078 |  | SNHG14 |  |
| -0.27 | 0.0152 | 0.993 | ENSG00000246731 |  | MGC16275 | uncharacterized protein MGC16275 |
| 0.527 | 0.0166 | 0.993 | ENSG00000235777 |  | DPYD-AS2 | DPYD antisense RNA 2 |
| -0.271 | 0.0172 | 0.993 | ENSG00000205913 |  | SRRM2-AS1 | SRRM2 antisense RNA 1 |
| 0.345 | 0.0173 | 0.993 | ENSG00000284968 |  | AC093827.4 |  |
| 0.682 | 0.0173 | 0.993 | ENSG00000262097 |  | LINC02185 | long intergenic non-protein coding RNA 2185 |
| 0.393 | 0.0178 | 0.993 | ENSG00000228506 |  | PNISR-AS1 |  |
| 0.406 | 0.0183 | 0.993 | ENSG00000245532 |  | NEAT1 | nuclear paraspeckle assembly transcript 1 |
| 0.459 | 0.0186 | 0.993 | ENSG00000228554 |  | AC0048375 |  |
| 0.716 | 0.0189 | 0.993 | ENSG00000286320 |  | LOC124900781 |  |
| 0.317 | 0.019 | 0.993 | ENSG00000273449 |  | RP11_218F103 |  |
| 0.533 | 0.0194 | 0.993 | ENSG00000238246 |  | RP11_575A192 |  |
| -0.526 | 0.0199 | 0.993 | ENSG00000277511 |  | CTD_2095E45 |  |
| 0.377 | 0.021 | 0.993 | ENSG00000258044 |  | LOC124902974 | uncharacterized LOC124902974 |
| -0.594 | 0.0218 | 0.993 | ENSG00000232229 |  | LINC00865 |  |
| 0.44 | 0.022 | 0.993 | ENSG00000251586 |  | TET2-AS1 |  |
| 0.307 | 0.0222 | 0.993 | ENSG00000237773 |  | LOC101927609 |  |
| -0.995 | 0.0229 | 0.993 | ENSG00000234336 |  | JAZF1-AS1 | JAZF1 antisense RNA 1 |
| -0.453 | 0.0231 | 0.993 | ENSG00000231680 |  | LINC02723 |  |
| -0.448 | 0.0242 | 0.993 | ENSG00000273314 |  | RP5_1136G132 |  |
| -0.302 | 0.0245 | 0.993 | ENSG00000215458 |  | AATBC | apoptosis associated transcript in bladder cancer |
| 0.483 | 0.0262 | 0.993 | ENSG00000270207 |  | RP11_157E161 |  |
| 0.369 | 0.0268 | 0.993 | ENSG00000290111 |  | ENSG00000290111 |  |
| -0.319 | 0.0273 | 0.993 | ENSG00000273329 |  | RP11_448A191 |  |
| -0.671 | 0.0287 | 0.993 | ENSG00000286904 |  | AC093675.1 |  |
| 0.439 | 0.0293 | 0.993 | ENSG00000228655 |  | AC0965581 |  |
| -0.435 | 0.0293 | 0.993 | ENSG00000285492 |  | LOC112267968 |  |
| 0.63 | 0.0297 | 0.993 | ENSG00000287613 |  | AC069549.2 |  |
| 0.398 | 0.0297 | 0.993 | ENSG00000281404 |  | LINC01176 | long intergenic non-protein coding RNA 1176 |
| -1.962 | 0.03 | 0.993 | ENSG00000246228 |  | CASC8 | cancer susceptibility 8 |
| -0.309 | 0.0306 | 0.993 | ENSG00000232973 |  | CYP1B1-AS1 | CYP1B1 antisense RNA 1 |
| -0.253 | 0.0315 | 0.993 | ENSG00000206337 |  | HCP5 | HLA complex P5 |
| 0.419 | 0.0337 | 0.993 | ENSG00000235888 |  | LINC02940 |  |
| -0.333 | 0.0348 | 0.993 | ENSG00000289295 |  | ENSG00000289295 |  |
| -0.331 | 0.0357 | 0.993 | ENSG00000272839 |  | RP11_452C131 |  |
| 0.475 | 0.0358 | 0.993 | ENSG00000285865 |  | AC010285.3 |  |
| -0.497 | 0.0359 | 0.993 | ENSG00000234484 |  | RP1_55C237 |  |
| -0.415 | 0.0362 | 0.993 | ENSG00000235314 |  | LINC00957 | long intergenic non-protein coding RNA 957 |
| -0.348 | 0.0369 | 0.993 | ENSG00000263069 |  | RNF213-AS1 | RNF213 antisense RNA 1 |
| 0.478 | 0.0387 | 0.993 | ENSG00000233559 |  | LINC00513 | long intergenic non-protein coding RNA 513 |
| -0.325 | 0.0392 | 0.993 | ENSG00000288398 |  | AL109627.1 |  |
| 0.338 | 0.0395 | 0.993 | ENSG00000289396 |  | ENSG00000289396 |  |
| -0.339 | 0.0395 | 0.993 | ENSG00000227076 |  | RP11_4C204 |  |
| 0.259 | 0.0397 | 0.993 | ENSG00000271862 |  | RP11_343L52 |  |
| 0.388 | 0.0402 | 0.993 | ENSG00000257277 |  | RP11_434H141 |  |
| 0.385 | 0.0405 | 0.993 | ENSG00000261654 |  | RP11_96K194 |  |
| 0.428 | 0.041 | 0.993 | ENSG00000242048 |  | PRKAG2-AS2 |  |
| -0.261 | 0.0413 | 0.993 | ENSG00000282851 |  | BISPR | BST2 interferon stimulated positive regulator |
| -0.42 | 0.0417 | 0.993 | ENSG00000256262 |  | USP30-AS1 | USP30 antisense RNA 1 |
| 0.45 | 0.0429 | 0.993 | ENSG00000244203 |  | FOXP1-AS1 | FOXP1 antisense RNA 1 |
| -0.319 | 0.0434 | 0.993 | ENSG00000259004 |  | LINC02285 | long intergenic non-protein coding RNA 2285 |
| 0.366 | 0.0442 | 0.993 | ENSG00000276334 |  | AL1332431 |  |
| 1.428 | 0.0447 | 0.993 | ENSG00000234147 |  | LOC102723724 |  |
| -0.383 | 0.045 | 0.993 | ENSG00000260285 |  | TRMT61A-DT | TRMT61A divergent transcript |
| -0.185 | 0.0451 | 0.993 | ENSG00000278133 |  | RP11_196G115 |  |
| 0.44 | 0.0468 | 0.993 | ENSG00000286997 |  | AC005856.1 |  |
| -0.491 | 0.0474 | 0.993 | ENSG00000267745 |  | RP11_686D228 |  |
| 0.332 | 0.0475 | 0.993 | ENSG00000274667 |  | RP11_31H53 |  |
| 0.452 | 0.0493 | 0.993 | ENSG00000286116 |  | AL157394.2 |  |
| -0.396 | 0.0498 | 0.993 | ENSG00000290043 |  | ENSG00000290043 |  |
| -0.209 | 0.0498 | 0.993 | ENSG00000203497 |  | PDCD4-AS1 | PDCD4 antisense RNA 1 |
| 0.578 | 0.0499 | 0.993 | ENSG00000249572 |  | TARS1-DT |  |

**Table S6**: All significant genes (p-value < 0.05) for the “microvascular vs. no vascular disease” comparison with the ENSEMBL ID and the IPA-identified gene SYMBOL.

| Expr Log Ratio | Expr p-value | Expr False Discovery Rate (q-value) | ID | Flags | Symbol | Entrez Gene Name |
| --- | --- | --- | --- | --- | --- | --- |
| 0.679 | 2.23E-05 | 0.0461 | ENSG00000261654 |  | RP11_96K194 |  |
| 0.478 | 0.00013 | 0.135 | ENSG00000260526 |  | AP1AR-DT | AP1AR divergent transcript |
| 0.259 | 0.000264 | 0.143 | ENSG00000285410 |  | GABPB1-IT1 | GABPB1 intronic transcript |
| 0.688 | 0.00034 | 0.143 | ENSG00000232542 |  | DPYD-IT1 | DPYD intronic transcript 1 |
| -0.349 | 0.000363 | 0.143 | ENSG00000260853 |  | NFATC2IP-AS1 | NFATC2IP antisense RNA 1 |
| 0.578 | 0.000416 | 0.143 | ENSG00000235888 |  | LINC02940 |  |
| -0.392 | 0.00083 | 0.245 | ENSG00000262879 |  | LOC101927060 |  |
| 0.342 | 0.00109 | 0.282 | ENSG00000289138 |  | ENSG00000289138 |  |
| 0.45 | 0.00123 | 0.282 | ENSG00000228506 |  | PNISR-AS1 |  |
| -2.075 | 0.00171 | 0.33 | ENSG00000253438 |  | PCAT1 | prostate cancer associated transcript 1 |
| -0.334 | 0.00176 | 0.33 | ENSG00000289102 |  | ENSG00000289102 |  |
| -1.067 | 0.00252 | 0.344 | ENSG00000225964 |  | NRIR | negative regulator of interferon response |
| 0.502 | 0.00261 | 0.344 | ENSG00000286145 |  | AC068282.1 |  |
| 0.335 | 0.00266 | 0.344 | ENSG00000237773 |  | LOC101927609 |  |
| -0.324 | 0.00276 | 0.344 | ENSG00000284428 |  | IPO5P1 | importin 5 pseudogene 1 |
| 0.533 | 0.00284 | 0.344 | ENSG00000289298 |  | ENSG00000289298 |  |
| -2.47 | 0.00292 | 0.344 | ENSG00000246228 |  | CASC8 | cancer susceptibility 8 |
| -0.434 | 0.00312 | 0.344 | ENSG00000238113 |  | LINC01410 | long intergenic non-protein coding RNA 1410 |
| -0.448 | 0.00316 | 0.344 | ENSG00000180539 |  | LINC02908 | long intergenic non-protein coding RNA 2908 |
| -0.446 | 0.00338 | 0.35 | ENSG00000284719 |  | AL033527.5 |  |
| 0.544 | 0.0037 | 0.354 | ENSG00000273129 |  | PACERR |  |
| -0.403 | 0.00379 | 0.354 | ENSG00000251364 |  | SYT9-AS1 |  |
| 0.4 | 0.00395 | 0.354 | ENSG00000275202 |  | RP11_156K233 |  |
| -0.287 | 0.0044 | 0.354 | ENSG00000286584 |  | LOC105374063 |  |
| -0.731 | 0.00472 | 0.354 | ENSG00000261326 |  | LINC01355 | long intergenic non-protein coding RNA 1355 |
| 0.441 | 0.00473 | 0.354 | ENSG00000231329 |  | RP1_225E122 |  |
| 0.725 | 0.00475 | 0.354 | ENSG00000242516 |  | LINC00960 | long intergenic non-protein coding RNA 960 |
| 0.332 | 0.00502 | 0.354 | ENSG00000251023 |  | RP11_549J181 |  |
| -0.43 | 0.0052 | 0.354 | ENSG00000260285 |  | TRMT61A-DT | TRMT61A divergent transcript |
| 0.484 | 0.00533 | 0.354 | ENSG00000282164 |  | PEG13 |  |
| -0.347 | 0.00546 | 0.354 | ENSG00000246451 |  | KLC1-AS1 | KLC1 antisense RNA 1 |
| 0.419 | 0.00548 | 0.354 | ENSG00000232564 |  | MRTFA-AS1 | MRTFA antisense RNA 1 |
| -0.391 | 0.00577 | 0.362 | ENSG00000271631 |  | SLC46A2-AS1 | SLC46A2 antisense RNA 1 |
| 0.363 | 0.00616 | 0.371 | ENSG00000276517 |  | AL1332432 |  |
| 0.441 | 0.00634 | 0.371 | ENSG00000251586 |  | TET2-AS1 |  |
| -0.62 | 0.00645 | 0.371 | ENSG00000231528 |  | FAM225A | family with sequence similarity 225 member A |
| -0.292 | 0.00723 | 0.404 | ENSG00000267278 |  | MAP3K14-AS1 | MAP3K14 antisense RNA 1 |
| -0.426 | 0.00745 | 0.406 | ENSG00000274460 |  | CTD_2649C142 |  |
| -0.35 | 0.00781 | 0.413 | ENSG00000260249 |  | LOC101927272 | uncharacterized LOC101927272 |
| -0.318 | 0.00799 | 0.413 | ENSG00000285693 |  | AP002381.2 |  |
| 0.514 | 0.0082 | 0.414 | ENSG00000233559 |  | LINC00513 | long intergenic non-protein coding RNA 513 |
| 0.338 | 0.00871 | 0.429 | ENSG00000226067 |  | LINC00623 | long intergenic non-protein coding RNA 623 |
| 0.385 | 0.00904 | 0.435 | ENSG00000269924 |  | RP11_697N184 |  |
| -0.395 | 0.00937 | 0.441 | ENSG00000231711 |  | LINC00899 | long intergenic non-protein coding RNA 899 |
| 0.262 | 0.00967 | 0.443 | ENSG00000261267 |  | RP11_44I103 |  |
| -0.276 | 0.0101 | 0.443 | ENSG00000286194 |  | AC138207.9 |  |
| -0.287 | 0.0101 | 0.443 | ENSG00000236144 |  | TMEM147-AS1 | TMEM147 antisense RNA 1 |
| 0.46 | 0.0103 | 0.443 | ENSG00000251139 |  | RP11_701P162 |  |
| -0.513 | 0.0106 | 0.444 | ENSG00000230724 |  | LINC01001 (includes others) | long intergenic non-protein coding RNA 1347 |
| 0.353 | 0.0111 | 0.444 | ENSG00000175611 |  | ERCC6L2-AS1 |  |
| -0.284 | 0.0113 | 0.444 | ENSG00000225880 |  | LINC00115 | long intergenic non-protein coding RNA 115 |
| -0.35 | 0.0115 | 0.444 | ENSG00000224888 |  | LOC100289580 | uncharacterized LOC100289580 |
| -0.293 | 0.0118 | 0.444 | ENSG00000285103 |  | AL451123.1 |  |
| 0.448 | 0.0122 | 0.444 | ENSG00000255026 |  | RP11_326C32 |  |
| -0.532 | 0.0125 | 0.444 | ENSG00000259377 |  | CTD_2308G161 |  |
| -0.329 | 0.0126 | 0.444 | ENSG00000204584 |  | FLJ45513 | uncharacterized LOC729220 |
| -0.328 | 0.0133 | 0.444 | ENSG00000257285 |  | PRMT5-DT |  |
| -0.222 | 0.0134 | 0.444 | ENSG00000264456 |  | RP11_848P12 |  |
| -0.397 | 0.0137 | 0.444 | ENSG00000225891 |  | DHDDS-AS1 | DHDDS antisense RNA 1 |
| -0.501 | 0.0138 | 0.444 | ENSG00000254528 |  | FXYD6-AS1 | FXYD6 antisense RNA 1 |
| 0.423 | 0.0139 | 0.444 | ENSG00000242048 |  | PRKAG2-AS2 |  |
| -0.332 | 0.0142 | 0.444 | ENSG00000265206 |  | MIR142HG | MIR142 host genes |
| -0.483 | 0.0143 | 0.444 | ENSG00000266896 |  | RP1_266L209 |  |
| 0.498 | 0.0144 | 0.444 | ENSG00000289557 |  | ENSG00000289557 |  |
| 0.433 | 0.0144 | 0.444 | ENSG00000270207 |  | RP11_157E161 |  |
| -0.277 | 0.0147 | 0.444 | ENSG00000269243 |  | CTD_2231E148 |  |
| -0.283 | 0.0147 | 0.444 | ENSG00000260051 |  | LA16C_390E64 |  |
| -0.414 | 0.0149 | 0.444 | ENSG00000242553 |  | AP00143214 |  |
| -0.261 | 0.0154 | 0.444 | ENSG00000280213 |  | UCKL1-AS1 | UCKL1 antisense RNA 1 |
| 0.15 | 0.0155 | 0.444 | ENSG00000272106 |  | RP11_345P49 |  |
| -0.353 | 0.016 | 0.444 | ENSG00000248714 |  | ZNF652-AS1 | ZNF652 antisense RNA 1 |
| -0.221 | 0.0161 | 0.444 | ENSG00000223960 |  | CHROMR | cholesterol induced regulator of metabolism RNA |
| 0.517 | 0.0162 | 0.444 | ENSG00000274265 |  | CH17_189H201 |  |
| 0.388 | 0.0163 | 0.444 | ENSG00000260661 |  | RP11_152L203 |  |
| 0.52 | 0.0163 | 0.444 | ENSG00000270681 |  | RP11_372K142 |  |
| 0.415 | 0.0163 | 0.444 | ENSG00000242154 |  | RP4_778K63 |  |
| -0.24 | 0.0166 | 0.444 | ENSG00000268205 |  | CTC_444N2411 |  |
| 0.408 | 0.0167 | 0.444 | ENSG00000228655 |  | AC0965581 |  |
| -0.231 | 0.0179 | 0.468 | ENSG00000290085 |  | ENSG00000290085 |  |
| 0.228 | 0.0182 | 0.469 | ENSG00000231770 |  | TMEM44-AS1 |  |
| 0.284 | 0.0183 | 0.469 | ENSG00000249898 |  | MCPH1-AS1 |  |
| -0.293 | 0.0191 | 0.481 | ENSG00000268471 |  | MIR4453HG | MIR4453 host gene |
| 0.178 | 0.0196 | 0.488 | ENSG00000273015 |  | LINC00938 | long intergenic non-protein coding RNA 938 |
| 0.328 | 0.0203 | 0.491 | ENSG00000235978 |  | LOC124906209 | uncharacterized LOC124906209 |
| 0.497 | 0.0204 | 0.491 | ENSG00000288994 |  | ENSG00000288994 |  |
| 0.336 | 0.0204 | 0.491 | ENSG00000259146 |  | SIPA1L1-AS1 |  |
| 0.461 | 0.0212 | 0.501 | ENSG00000288737 |  | ENSG00000288737 |  |
| -0.252 | 0.0214 | 0.501 | ENSG00000228315 |  | GUSBP11 | GUSB pseudogene 11 |
| -0.466 | 0.0215 | 0.501 | ENSG00000255050 |  | RP11_661A129 |  |
| -0.234 | 0.0227 | 0.501 | ENSG00000268030 |  | AC0052532 |  |
| -0.239 | 0.0228 | 0.501 | ENSG00000285533 |  | RELA-DT |  |
| -0.198 | 0.0231 | 0.501 | ENSG00000215067 |  | ALOX12-AS1 | ALOX12 antisense RNA 1 |
| 0.371 | 0.0238 | 0.501 | ENSG00000235903 |  | CPB2-AS1 |  |
| 0.247 | 0.0238 | 0.501 | ENSG00000288990 |  | ENSG00000288990 |  |
| -0.309 | 0.024 | 0.501 | ENSG00000273137 |  | SELENOO-AS1 |  |
| -0.316 | 0.0252 | 0.501 | ENSG00000258317 |  | RP11_603J245 |  |
| 0.402 | 0.0255 | 0.501 | ENSG00000288742 |  | ENSG00000288742 |  |
| -0.305 | 0.0255 | 0.501 | ENSG00000227508 |  | LINC01624 | long intergenic non-protein coding RNA 1624 |
| -0.313 | 0.0256 | 0.501 | ENSG00000279529 |  | CTD_3222D198 |  |
| 0.201 | 0.026 | 0.501 | ENSG00000289050 |  | ENSG00000289050 |  |
| -0.228 | 0.0262 | 0.501 | ENSG00000261971 |  | MMP25-AS1 | MMP25 antisense RNA 1 |
| -0.263 | 0.0263 | 0.501 | ENSG00000270120 |  | RP11_327F226 |  |
| -0.283 | 0.0264 | 0.501 | ENSG00000267724 |  | RP11_49K248 |  |
| -0.584 | 0.0265 | 0.501 | ENSG00000241860 |  | LOC124900384 |  |
| -0.453 | 0.0267 | 0.501 | ENSG00000287338 |  | LOC107985323 |  |
| 0.364 | 0.0268 | 0.501 | ENSG00000238142 |  | LOC105376805 |  |
| -0.316 | 0.0271 | 0.501 | ENSG00000224934 |  | GOT1-DT |  |
| -0.233 | 0.0272 | 0.501 | ENSG00000234771 |  | SLC25A25-AS1 | SLC25A25 antisense RNA 1 |
| -0.333 | 0.0274 | 0.501 | ENSG00000275854 |  | RP11_278C75 |  |
| -0.263 | 0.0277 | 0.501 | ENSG00000260005 |  | LOC105371925 |  |
| 0.353 | 0.0278 | 0.501 | ENSG00000205622 |  | ETS2-AS1 | ETS2 antisense RNA 1 |
| -0.301 | 0.0278 | 0.501 | ENSG00000272825 |  | LL21NC02_1C162 |  |
| -0.293 | 0.0282 | 0.501 | ENSG00000268798 |  | CTB_25B135 |  |
| 0.423 | 0.029 | 0.501 | ENSG00000247925 |  | RP3_510L91 |  |
| 0.322 | 0.0294 | 0.501 | ENSG00000290019 |  | ENSG00000290019 |  |
| 0.295 | 0.0295 | 0.501 | ENSG00000289340 |  | ENSG00000289340 |  |
| -0.443 | 0.0295 | 0.501 | ENSG00000267745 |  | RP11_686D228 |  |
| -0.26 | 0.0298 | 0.501 | ENSG00000285979 |  | AC009090.6 |  |
| 0.223 | 0.0298 | 0.501 | ENSG00000267321 |  | SNHG30 |  |
| -0.254 | 0.0299 | 0.501 | ENSG00000286196 |  | AL162586.2 |  |
| 0.337 | 0.03 | 0.501 | ENSG00000290097 |  | ENSG00000290097 |  |
| -0.273 | 0.0303 | 0.501 | ENSG00000272140 |  | RP11_574K1129 |  |
| -0.243 | 0.0303 | 0.501 | ENSG00000255306 |  | RP5_901A41 |  |
| -0.245 | 0.0306 | 0.501 | ENSG00000228606 |  | DCAF8-DT | DCAF8 divergent transcript |
| -0.2 | 0.0309 | 0.501 | ENSG00000230113 |  | AC0911771 |  |
| 0.392 | 0.0312 | 0.501 | ENSG00000286236 |  | AC018755.5 |  |
| -0.319 | 0.0315 | 0.501 | ENSG00000285725 |  | AC004967.2 |  |
| 0.392 | 0.0318 | 0.501 | ENSG00000289259 |  | ENSG00000289259 |  |
| -0.306 | 0.0319 | 0.501 | ENSG00000232912 |  | RERE-AS1 | RERE antisense RNA 1 |
| 0.428 | 0.032 | 0.501 | ENSG00000260517 |  | RP11_426C225 |  |
| -0.313 | 0.032 | 0.501 | ENSG00000254827 |  | SLC22A18AS | SLC22A18 antisense RNA |
| 0.399 | 0.0323 | 0.501 | ENSG00000283959 |  | LOC101927245 |  |
| -0.342 | 0.0327 | 0.501 | ENSG00000230325 |  | RP11_385F54 |  |
| -0.248 | 0.0328 | 0.501 | ENSG00000228192 |  | ZNF691-DT | ZNF691 divergent transcript |
| 0.201 | 0.0334 | 0.501 | ENSG00000272686 |  | WASL-DT | WASL divergent transcript |
| -0.226 | 0.0336 | 0.501 | ENSG00000250166 |  | C2CD5-AS1 |  |
| -0.312 | 0.0337 | 0.501 | ENSG00000273066 |  | RP11_216L1319 |  |
| -0.279 | 0.0338 | 0.501 | ENSG00000288663 |  | AC073611.1 |  |
| -0.281 | 0.0338 | 0.501 | ENSG00000261592 |  | RP11_178L83 |  |
| 0.255 | 0.0339 | 0.501 | ENSG00000227543 |  | SPAG5-AS1 | SPAG5 antisense RNA 1 |
| -0.257 | 0.0347 | 0.509 | ENSG00000272630 |  | RP11_344N105 |  |
| 0.401 | 0.0355 | 0.509 | ENSG00000289123 |  | ENSG00000289123 |  |
| -0.27 | 0.0357 | 0.509 | ENSG00000183250 |  | LINC01547 | long intergenic non-protein coding RNA 1547 |
| -0.34 | 0.0358 | 0.509 | ENSG00000282572 |  | FAM157D |  |
| -0.234 | 0.0359 | 0.509 | ENSG00000265743 |  | LINC02978 | long intergenic non-protein coding RNA 2978 |
| 0.725 | 0.036 | 0.509 | ENSG00000269927 |  | RP6_91H83 |  |
| -0.242 | 0.0362 | 0.509 | ENSG00000267344 |  | CTB_39G83 |  |
| -0.214 | 0.0368 | 0.511 | ENSG00000237491 |  | LINC01409 |  |
| -0.503 | 0.0372 | 0.511 | ENSG00000286904 |  | AC093675.1 |  |
| -0.324 | 0.0379 | 0.511 | ENSG00000244879 |  | GABPB1-AS1 | GABPB1 antisense RNA 1 |
| 0.289 | 0.0382 | 0.511 | ENSG00000287665 |  | AC092428.1 |  |
| -0.448 | 0.0384 | 0.511 | ENSG00000225138 |  | SLC9A3-AS1 | SLC9A3 antisense RNA 1 |
| 0.932 | 0.0388 | 0.511 | ENSG00000229502 |  | RP11_52J32 |  |
| -0.257 | 0.0389 | 0.511 | ENSG00000290124 |  | ENSG00000290124 |  |
| -0.245 | 0.0389 | 0.511 | ENSG00000249771 |  | LOC105374426 |  |
| -0.22 | 0.0394 | 0.511 | ENSG00000215458 |  | AATBC | apoptosis associated transcript in bladder cancer |
| -0.373 | 0.0397 | 0.511 | ENSG00000287562 |  | AL109615.4 |  |
| 0.25 | 0.0398 | 0.511 | ENSG00000273148 |  | LCDR | lysosome cell death regulator |
| -0.191 | 0.0399 | 0.511 | ENSG00000215068 |  | ANXA2R-AS1 |  |
| 0.375 | 0.04 | 0.511 | ENSG00000255443 |  | CD44-AS1 | CD44 antisense RNA 1 |
| -0.563 | 0.04 | 0.511 | ENSG00000286092 |  | LOC105370042 |  |
| -0.286 | 0.04 | 0.511 | ENSG00000247373 |  | TMED2-DT | TMED2 divergent transcript |
| 0.3 | 0.0406 | 0.512 | ENSG00000266983 |  | RANBP3-DT |  |
| 0.286 | 0.0408 | 0.512 | ENSG00000289341 |  | ENSG00000289341 |  |
| -0.2 | 0.0414 | 0.512 | ENSG00000226332 |  | RP11_157P14 |  |
| 0.318 | 0.0416 | 0.512 | ENSG00000277496 |  | SLCO4A1-AS2 |  |
| -0.281 | 0.0418 | 0.512 | ENSG00000288838 |  | ENSG00000288838 |  |
| -0.319 | 0.0422 | 0.512 | ENSG00000227039 |  | ITGB2-AS1 |  |
| -0.254 | 0.0434 | 0.512 | ENSG00000267121 |  | FMNL1-DT | FMNL1 divergent transcript |
| -0.127 | 0.0436 | 0.512 | ENSG00000231074 |  | HCG18 | HLA complex group 18 |
| -0.318 | 0.0441 | 0.512 | ENSG00000273243 |  | CTA_217C22 |  |
| -0.316 | 0.0441 | 0.512 | ENSG00000268230 |  | CTD_2619J138 |  |
| -0.207 | 0.0442 | 0.512 | ENSG00000228242 |  | XPC-AS1 |  |
| -0.228 | 0.0443 | 0.512 | ENSG00000281195 |  | ZNF638-IT1 |  |
| 0.246 | 0.0445 | 0.512 | ENSG00000272918 |  | CTB_152G176 |  |
| 0.294 | 0.0453 | 0.512 | ENSG00000270175 |  | RP11_793H1311 |  |
| -0.331 | 0.0453 | 0.512 | ENSG00000257058 |  | RP11_864I44 |  |
| -0.228 | 0.0454 | 0.512 | ENSG00000269352 |  | PTOV1-AS2 | PTOV1 antisense RNA 2 |
| -0.355 | 0.0455 | 0.512 | ENSG00000286276 |  | AL390066.2 |  |
| 0.334 | 0.0455 | 0.512 | ENSG00000274818 |  | RP1_292L203 |  |
| 0.249 | 0.0456 | 0.512 | ENSG00000286153 |  | AP000331.1 |  |
| 0.372 | 0.0456 | 0.512 | ENSG00000244203 |  | FOXP1-AS1 | FOXP1 antisense RNA 1 |
| 1.183 | 0.0456 | 0.512 | ENSG00000276603 |  | RP11_425M57 |  |
| 0.255 | 0.0461 | 0.512 | ENSG00000255135 |  | EMSY-DT | EMSY divergent transcript |
| -0.216 | 0.0467 | 0.512 | ENSG00000229873 |  | OGFR-AS1 | OGFR antisense RNA 1 |
| 0.35 | 0.0472 | 0.512 | ENSG00000267476 |  | RP11_126O14 |  |
| -0.287 | 0.0472 | 0.512 | ENSG00000273855 |  | RP11_133K112 |  |
| 0.226 | 0.0476 | 0.512 | ENSG00000289443 |  | ENSG00000289443 |  |
| 0.209 | 0.0476 | 0.512 | ENSG00000239569 |  | KMT2E-AS1 |  |
| -0.389 | 0.0476 | 0.512 | ENSG00000262580 |  | RP11_334C175 |  |
| -0.324 | 0.0479 | 0.512 | ENSG00000228784 |  | LINC00954 | long intergenic non-protein coding RNA 954 |
| -0.243 | 0.0483 | 0.512 | ENSG00000286067 |  | AC004263.2 |  |
| 0.401 | 0.0484 | 0.512 | ENSG00000232878 |  | DPYD-AS1 | DPYD antisense RNA 1 |
| -0.33 | 0.0485 | 0.512 | ENSG00000256262 |  | USP30-AS1 | USP30 antisense RNA 1 |
| 0.381 | 0.0494 | 0.512 | ENSG00000265316 |  | RP11_286N31 |  |
| 0.305 | 0.0495 | 0.512 | ENSG00000232043 |  | RP4_530I159 |  |
| -0.336 | 0.0496 | 0.512 | ENSG00000261471 |  | RP11_61F121 |  |
| 0.42 | 0.0497 | 0.512 | ENSG00000231881 |  | RP5_1120P113 |  |

**Table S7:** All significant genes (p-value < 0.05) for the “macrovascular vs no vascular disease” comparison with the ENSEMBL ID and the IPA-identified gene SYMBOL.

| Expr Log Ratio | Expr p-value | Expr False Discovery Rate (q-value) | ID | Flags | Symbol | Entrez Gene Name |
| --- | --- | --- | --- | --- | --- | --- |
| 0.466 | 0.000722 | 1 | ENSG00000179818 |  | PCBP1-AS1 | PCBP1 antisense RNA 1 |
| -1.497 | 0.00235 | 1 | ENSG00000167920 |  | KRT10-AS1 | KRT10 antisense RNA 1 |
| -1.146 | 0.00276 | 1 | ENSG00000287104 |  | AC097382.3 |  |
| -2.165 | 0.00288 | 1 | ENSG00000289189 |  | LOC105375170 |  |
| -1.021 | 0.00572 | 1 | ENSG00000232098 |  | ZNF584-DT | ZNF584 divergent transcript |
| -1.114 | 0.00907 | 1 | ENSG00000275927 |  | RP11_567P191 |  |
| -0.821 | 0.00985 | 1 | ENSG00000270344 |  | POC1B-AS1 |  |
| 1.483 | 0.0105 | 1 | ENSG00000264829 |  | RP11_474I118 |  |
| -0.995 | 0.0115 | 1 | ENSG00000235314 |  | LINC00957 | long intergenic non-protein coding RNA 957 |
| -1.21 | 0.0125 | 1 | ENSG00000215014 |  | RP5_832C25 |  |
| 0.815 | 0.0141 | 1 | ENSG00000238279 |  | LOC124904423 | uncharacterized LOC124904423 |
| -0.83 | 0.0141 | 1 | ENSG00000237149 |  | ZNF503-AS2 | ZNF503 antisense RNA 2 |
| 0.737 | 0.0142 | 1 | ENSG00000228340 |  | MIR646HG | MIR646 host gene |
| 0.652 | 0.0154 | 1 | ENSG00000234945 |  | GTF3C2-AS1 | GTF3C2 antisense RNA 1 |
| -0.706 | 0.0175 | 1 | ENSG00000267254 |  | ZNF790-AS1 |  |
| -0.924 | 0.0179 | 1 | ENSG00000231680 |  | LINC02723 |  |
| -0.734 | 0.0182 | 1 | ENSG00000271780 |  | RP11_1017G215 |  |
| -0.739 | 0.0182 | 1 | ENSG00000232354 |  | VIPR1-AS1 | VIPR1 antisense RNA 1 |
| -0.491 | 0.0184 | 1 | ENSG00000246731 |  | MGC16275 | uncharacterized protein MGC16275 |
| -1.201 | 0.0221 | 1 | ENSG00000232229 |  | LINC00865 |  |
| -0.884 | 0.0228 | 1 | ENSG00000236901 |  | MIR600HG | MIR600 host gene |
| 0.792 | 0.0234 | 1 | ENSG00000233006 |  | MIR3936HG | MIR3936 host gene |
| -2.376 | 0.0243 | 1 | ENSG00000289608 |  | ENSG00000289608 |  |
| -0.761 | 0.0253 | 1 | ENSG00000268947 |  | AD0006842 |  |
| -0.717 | 0.0254 | 1 | ENSG00000230587 |  | LINC02580 |  |
| -0.807 | 0.0255 | 1 | ENSG00000162888 |  | IKBKE-AS1 | IKBKE antisense RNA 1 |
| 0.802 | 0.0263 | 1 | ENSG00000223704 |  | LINC01422 | long intergenic non-protein coding RNA 1422 |
| -0.728 | 0.0267 | 1 | ENSG00000230454 |  | U731662 |  |
| -0.652 | 0.0267 | 1 | ENSG00000228889 |  | UBAC2-AS1 |  |
| 1.562 | 0.0272 | 1 | ENSG00000270127 |  | RP11-526I2.5 |  |
| -2.451 | 0.0282 | 1 | ENSG00000229153 |  | EPHA1-AS1 | EPHA1 antisense RNA 1 |
| 1.263 | 0.0291 | 1 | ENSG00000289470 |  | ENSG00000289470 |  |
| -0.758 | 0.0294 | 1 | ENSG00000254827 |  | SLC22A18AS | SLC22A18 antisense RNA |
| -0.845 | 0.0296 | 1 | ENSG00000267481 |  | CTC_559E95 |  |
| -0.627 | 0.0322 | 1 | ENSG00000272654 |  | JTB-DT | JTB divergent transcript |
| 0.322 | 0.0329 | 1 | ENSG00000272888 |  | CHASERR | CHD2 adjacent suppressive regulatory RNA |
| -1.157 | 0.033 | 1 | ENSG00000225138 |  | SLC9A3-AS1 | SLC9A3 antisense RNA 1 |
| -0.736 | 0.0337 | 1 | ENSG00000277851 |  | LINC02391 |  |
| 0.938 | 0.034 | 1 | ENSG00000264270 |  | RP11_474I117 |  |
| -0.662 | 0.0358 | 1 | ENSG00000288973 |  | ENSG00000288973 |  |
| -0.722 | 0.0363 | 1 | ENSG00000240498 |  | CDKN2B-AS1 | CDKN2B antisense RNA 1 |
| -0.573 | 0.0371 | 1 | ENSG00000234028 |  | EIF2AK3-DT |  |
| 0.975 | 0.0373 | 1 | ENSG00000225783 |  | MIAT | myocardial infarction associated transcript |
| 0.698 | 0.0377 | 1 | ENSG00000282572 |  | FAM157D |  |
| 0.487 | 0.0418 | 1 | ENSG00000289554 |  | ENSG00000289554 |  |
| -0.785 | 0.0421 | 1 | ENSG00000231170 |  | PDK4-AS1 |  |
| 0.582 | 0.0425 | 1 | ENSG00000285852 |  | LOC124902388 | uncharacterized LOC124902388 |
| -0.544 | 0.0427 | 1 | ENSG00000262879 |  | LOC101927060 |  |
| 0.818 | 0.0434 | 1 | ENSG00000273302 |  | RP11_493E122 |  |
| -0.599 | 0.0443 | 1 | ENSG00000236548 |  | RNF217-AS1 |  |
| 0.865 | 0.046 | 1 | ENSG00000270681 |  | RP11_372K142 |  |
| 0.801 | 0.0473 | 1 | ENSG00000260719 |  | AC00913317 |  |
| -0.547 | 0.0486 | 1 | ENSG00000238058 |  | CAMSAP1-DT |  |

**Table S8:** All significant networks identified by IPA for the micro and macrovascular phenotypes.


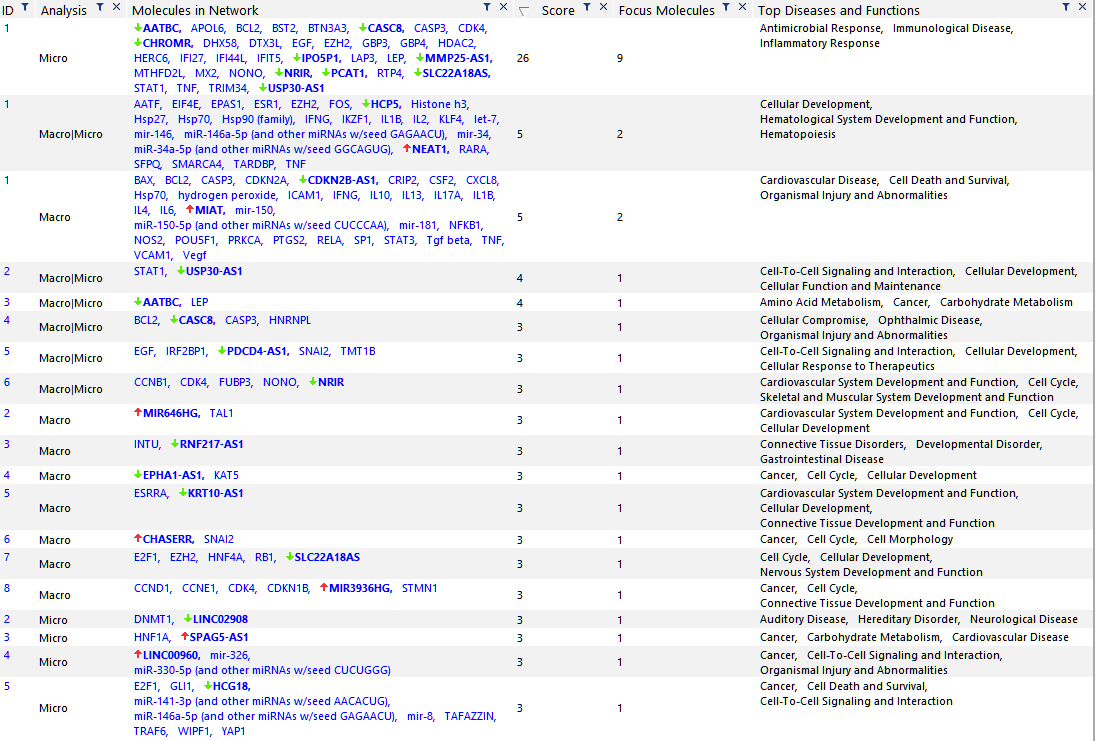


**Table S9:** All significant genes (p-value < 0.05) for diabetes duration ≥ 10 years vs diabetes duration < 10 years.

| Expr Log Ratio | Expr p-value | Expr False Discovery Rate (q-value) | ID | Flags | Symbol | Entrez Gene Name |
| --- | --- | --- | --- | --- | --- | --- |
| 0.43 | 0.000155 | 0.32 | ENSG00000251259 |  | AC0040692 |  |
| 0.618 | 0.00236 | 0.999 | ENSG00000224478 |  | RP11_13P51 |  |
| 0.366 | 0.00475 | 0.999 | ENSG00000265100 |  | RP11_147L132 |  |
| -0.476 | 0.0051 | 0.999 | ENSG00000214922 |  | HLA-F-AS1 | HLA-F antisense RNA 1 |
| -0.308 | 0.00512 | 0.999 | ENSG00000263327 |  | TAPT1-AS1 | TAPT1 antisense RNA 1 (head to head) |
| 0.396 | 0.006 | 0.999 | ENSG00000260708 |  | TBC1D22A-DT | TBC1D22A divergent transcript |
| -0.447 | 0.00661 | 0.999 | ENSG00000255893 |  | RP11_685N101 |  |
| 1.1 | 0.00774 | 0.999 | ENSG00000225964 |  | NRIR | negative regulator of interferon response |
| 0.43 | 0.0078 | 0.999 | ENSG00000213062 |  | RP1_206D156 |  |
| 0.42 | 0.00821 | 0.999 | ENSG00000289234 |  | ZNF496-DT |  |
| -0.248 | 0.00854 | 0.999 | ENSG00000261799 |  | RP11_283I36 |  |
| 0.449 | 0.00932 | 0.999 | ENSG00000273565 |  | CTD_3075F151 |  |
| 0.614 | 0.00986 | 0.999 | ENSG00000288807 |  | ENSG00000288807 |  |
| 0.438 | 0.00987 | 0.999 | ENSG00000267212 |  | CTD_2659N199 |  |
| 0.731 | 0.0105 | 0.999 | ENSG00000215196 |  | BASP1-AS1 | BASP1 antisense RNA 1 |
| -0.355 | 0.0109 | 0.999 | ENSG00000175611 |  | ERCC6L2-AS1 |  |
| 0.278 | 0.011 | 0.999 | ENSG00000287817 |  | AL096701.4 |  |
| 0.57 | 0.0119 | 0.999 | ENSG00000231881 |  | RP5_1120P113 |  |
| 0.464 | 0.0123 | 0.999 | ENSG00000234261 |  | RP11_146I21 |  |
| 0.4 | 0.0166 | 0.999 | ENSG00000271869 |  | DCTN6-DT | DCTN6 divergent transcript |
| 0.384 | 0.0169 | 0.999 | ENSG00000272666 |  | KLHDC7B-DT | KLHDC7B divergent transcript |
| -0.309 | 0.0177 | 0.999 | ENSG00000226281 |  | RP1_80N22 |  |
| 0.906 | 0.0181 | 0.999 | ENSG00000272512 |  | RP11_54O717 |  |
| -0.219 | 0.0185 | 0.999 | ENSG00000272758 |  | WDR5B-DT |  |
| -0.292 | 0.0204 | 0.999 | ENSG00000289698 |  | ENSG00000289698 |  |
| 0.902 | 0.0205 | 0.999 | ENSG00000237989 |  | LINC01679 |  |
| 0.26 | 0.0208 | 0.999 | ENSG00000270083 |  | RP1_257I2014 |  |
| -0.289 | 0.0213 | 0.999 | ENSG00000289156 |  | LOC107985892 |  |
| 0.422 | 0.0219 | 0.999 | ENSG00000286236 |  | AC018755.5 |  |
| 0.261 | 0.022 | 0.999 | ENSG00000273188 |  | RP3_402G1125 |  |
| -0.255 | 0.0236 | 0.999 | ENSG00000260236 |  | PTPN23-DT | PTPN23 divergent transcript |
| -0.344 | 0.0237 | 0.999 | ENSG00000272812 |  | RP5_855D213 |  |
| 0.209 | 0.0251 | 0.999 | ENSG00000223960 |  | CHROMR | cholesterol induced regulator of metabolism RNA |
| 0.282 | 0.0254 | 0.999 | ENSG00000260077 |  | RP11_254F72 |  |
| 0.353 | 0.0258 | 0.999 | ENSG00000236255 |  | AC0094042 |  |
| 0.205 | 0.0259 | 0.999 | ENSG00000226824 |  | LOC100996437 |  |
| -0.41 | 0.0259 | 0.999 | ENSG00000229739 |  | PDC-AS1 | PDC antisense RNA 1 |
| -0.205 | 0.0266 | 0.999 | ENSG00000197182 |  | MIRLET7BHG | MIRLET7B host gene |
| -0.525 | 0.0271 | 0.999 | ENSG00000276216 |  | CH17-373J23.1 |  |
| -0.345 | 0.0271 | 0.999 | ENSG00000287697 |  | Z99127.4 |  |
| -0.196 | 0.0275 | 0.999 | ENSG00000254703 |  | SENCR |  |
| -0.219 | 0.0284 | 0.999 | ENSG00000263072 |  | ZNF213-AS1 |  |
| -0.291 | 0.0292 | 0.999 | ENSG00000281162 |  | LINC01127 |  |
| -0.585 | 0.0301 | 0.999 | ENSG00000287431 |  | RENO1 | regulator of early neurogenesis 1 |
| 0.692 | 0.0302 | 0.999 | ENSG00000290034 |  | ENSG00000290034 |  |
| 0.282 | 0.0304 | 0.999 | ENSG00000288827 |  | ENSG00000288827 |  |
| -0.224 | 0.0305 | 0.999 | ENSG00000228302 |  | RP11_186N153 |  |
| 0.296 | 0.0308 | 0.999 | ENSG00000270640 |  | RP11_373D232 |  |
| -0.297 | 0.031 | 0.999 | ENSG00000250397 |  | RP11_1391J71 |  |
| 1.887 | 0.0319 | 0.999 | ENSG00000246228 |  | CASC8 | cancer susceptibility 8 |
| 0.256 | 0.0327 | 0.999 | ENSG00000186594 |  | MIR22HG | MIR22 host gene |
| -0.349 | 0.0336 | 0.999 | ENSG00000277130 |  | RP11_530C54 |  |
| 0.257 | 0.034 | 0.999 | ENSG00000261526 |  | CTB_31O202 |  |
| 0.272 | 0.0352 | 0.999 | ENSG00000264577 |  | AC010761.8 |  |
| 0.238 | 0.0371 | 0.999 | ENSG00000273253 |  | TRABD-AS1 | TRABD antisense RNA 1 |
| 0.371 | 0.0374 | 0.999 | ENSG00000267476 |  | RP11_126O14 |  |
| -0.387 | 0.0374 | 0.999 | ENSG00000231104 |  | RP11_354M203 |  |
| 0.267 | 0.0377 | 0.999 | ENSG00000236008 |  | LINC01814 | long intergenic non-protein coding RNA 1814 |
| -0.667 | 0.0378 | 0.999 | ENSG00000245164 |  | LINC00861 |  |
| 0.264 | 0.0388 | 0.999 | ENSG00000290124 |  | ENSG00000290124 |  |
| -0.327 | 0.0395 | 0.999 | ENSG00000257497 |  | GLIPR1-AS1 | GLIPR1 antisense RNA 1 |
| -0.314 | 0.0402 | 0.999 | ENSG00000232098 |  | ZNF584-DT | ZNF584 divergent transcript |
| -0.397 | 0.0405 | 0.999 | ENSG00000286035 |  | AC073840.1 |  |
| -0.375 | 0.041 | 0.999 | ENSG00000275278 |  | RP11_946L162 |  |
| 0.331 | 0.0415 | 0.999 | ENSG00000244879 |  | GABPB1-AS1 | GABPB1 antisense RNA 1 |
| -0.281 | 0.0416 | 0.999 | ENSG00000290111 |  | ENSG00000290111 |  |
| 0.364 | 0.0417 | 0.999 | ENSG00000289015 |  | ENSG00000289015 |  |
| 0.374 | 0.0433 | 0.999 | ENSG00000286223 |  | AC064836.4 |  |
| -0.462 | 0.0436 | 0.999 | ENSG00000286786 |  | AC116158.3 |  |
| 0.287 | 0.0446 | 0.999 | ENSG00000251323 |  | LINC02728 | long intergenic non-protein coding RNA 2728 |
| 0.91 | 0.0457 | 0.999 | ENSG00000273018 |  | FAM106A | family with sequence similarity 106 member A |
| -0.479 | 0.0471 | 0.999 | ENSG00000235770 |  | LINC00607 | long intergenic non-protein coding RNA 607 |
| -0.31 | 0.0475 | 0.999 | ENSG00000276718 |  | VAMP1-AS1 | VAMP1 antisense RNA 1 |

**Table S10:** All significant networks identified by IPA for the diabetes duration comparison.

**
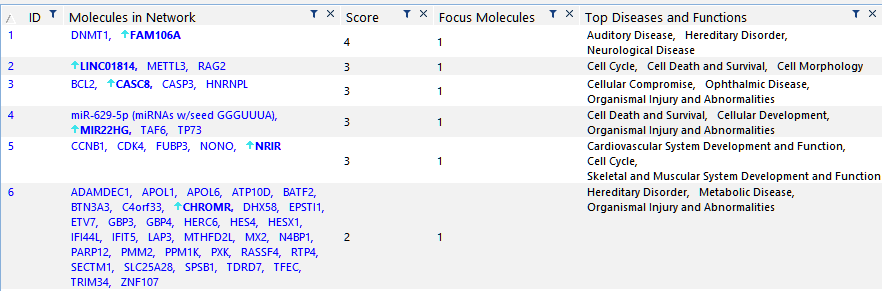
**

**Table S11:** All significant genes (p-value < 0.05) for “Good vs Poor” diabetes control comparison.

| Expr Log Ratio | Expr p-value | Expr False Discovery Rate (q-value) | ID | Flags | Symbol | Entrez Gene Name |
| --- | --- | --- | --- | --- | --- | --- |
| -0.581 | 0.000143 | 0.159 | ENSG00000290074 |  | ENSG00000290074 |  |
| -0.85 | 0.000154 | 0.159 | ENSG00000231881 |  | RP5_1120P113 |  |
| 0.609 | 0.000789 | 0.332 | ENSG00000287822 |  | LOC101928007 |  |
| -0.649 | 0.00133 | 0.332 | ENSG00000248323 |  | LUCAT1 | lung cancer associated transcript 1 |
| 0.673 | 0.00139 | 0.332 | ENSG00000213373 |  | LINC00671 | long intergenic non-protein coding RNA 671 |
| -0.466 | 0.00146 | 0.332 | ENSG00000289235 |  | ENSG00000289235 |  |
| 0.487 | 0.00189 | 0.332 | ENSG00000287095 |  | AF228727.1 |  |
| -0.958 | 0.00226 | 0.332 | ENSG00000289888 |  | ENSG00000289888 |  |
| 1.482 | 0.0023 | 0.332 | ENSG00000225231 |  | LINC02470 | long intergenic non-protein coding RNA 2470 |
| -0.884 | 0.00234 | 0.332 | ENSG00000236700 |  | LINC01010 | long intergenic non-protein coding RNA 1010 |
| -0.42 | 0.00238 | 0.332 | ENSG00000258738 |  | BAZ1A-AS1 | BAZ1A antisense RNA 1 |
| 0.688 | 0.00257 | 0.332 | ENSG00000249476 |  | LOC285638 | uncharacterized LOC285638 |
| -0.388 | 0.00291 | 0.332 | ENSG00000290062 |  | ENSG00000290062 |  |
| -0.813 | 0.00304 | 0.332 | ENSG00000289970 |  | ENSG00000289970 |  |
| 0.378 | 0.00305 | 0.332 | ENSG00000286599 |  | AC110611.1 |  |
| 0.35 | 0.00307 | 0.332 | ENSG00000278206 |  | RP1_20N28 |  |
| -0.316 | 0.00317 | 0.332 | ENSG00000230555 |  | LINC02916 | long intergenic non-protein coding RNA 2916 |
| -0.429 | 0.00331 | 0.332 | ENSG00000261888 |  | AC1448311 |  |
| -0.749 | 0.00334 | 0.332 | ENSG00000250274 |  | LOC100128059 | uncharacterized LOC100128059 |
| 0.386 | 0.00341 | 0.332 | ENSG00000223745 |  | CCDC18-AS1 | CCDC18 antisense RNA 1 |
| -0.569 | 0.00349 | 0.332 | ENSG00000288737 |  | ENSG00000288737 |  |
| 0.298 | 0.00353 | 0.332 | ENSG00000237883 |  | DGUOK-AS1 |  |
| 0.362 | 0.00398 | 0.348 | ENSG00000281026 |  | N4BP2L2-IT2 | N4BPL2 intronic transcript 2 |
| -0.45 | 0.00404 | 0.348 | ENSG00000267519 |  | MIR23AHG | miR-23a/27a/24-2 cluster host gene |
| -0.449 | 0.00438 | 0.362 | ENSG00000266970 |  | SOCS3-DT |  |
| 0.303 | 0.00476 | 0.362 | ENSG00000259291 |  | ZNF710-AS1 |  |
| -0.313 | 0.00488 | 0.362 | ENSG00000229124 |  | VIM-AS1 | VIM antisense RNA 1 |
| -0.355 | 0.0049 | 0.362 | ENSG00000253736 |  | LOC105377730 |  |
| -0.524 | 0.00544 | 0.362 | ENSG00000251393 |  | RP1_240K63 |  |
| 0.32 | 0.00559 | 0.362 | ENSG00000230084 |  | ZBTB47-AS1 | ZBTB47 and NKTR antisense RNA 1 |
| -0.375 | 0.00592 | 0.362 | ENSG00000230074 |  | RP11_195F199 |  |
| -0.39 | 0.00608 | 0.362 | ENSG00000228382 |  | ITPKB-IT1 | ITPKB intronic transcript 1 |
| -0.642 | 0.00625 | 0.362 | ENSG00000272506 |  | RP4_535B204 |  |
| 0.337 | 0.00629 | 0.362 | ENSG00000224616 |  | RTCA-AS1 | RTCA antisense RNA 1 |
| -0.43 | 0.00641 | 0.362 | ENSG00000290043 |  | ENSG00000290043 |  |
| -0.413 | 0.00667 | 0.362 | ENSG00000286186 |  | LOC101927897 |  |
| 0.503 | 0.00681 | 0.362 | ENSG00000256712 |  | RP11_785H52 |  |
| 0.402 | 0.00691 | 0.362 | ENSG00000286018 |  | AF129075.3 |  |
| 0.318 | 0.00697 | 0.362 | ENSG00000247903 |  | RP11_421F163 |  |
| 0.276 | 0.007 | 0.362 | ENSG00000261596 |  | CTB_31N193 |  |
| -0.461 | 0.00747 | 0.369 | ENSG00000275210 |  | AC0089842 |  |
| -0.409 | 0.00748 | 0.369 | ENSG00000232043 |  | RP4_530I159 |  |
| 0.291 | 0.00831 | 0.384 | ENSG00000232940 |  | HCG25 | HLA complex group 25 |
| 0.302 | 0.0085 | 0.384 | ENSG00000289306 |  | ENSG00000289306 |  |
| -0.321 | 0.00883 | 0.384 | ENSG00000258082 |  | RP11_443B73 |  |
| 0.274 | 0.00903 | 0.384 | ENSG00000285632 |  | AC084024.4 |  |
| -0.542 | 0.00903 | 0.384 | ENSG00000249173 |  | LINC01093 | long intergenic non-protein coding RNA 1093 |
| 0.259 | 0.0092 | 0.384 | ENSG00000264112 |  | RP11_159D122 |  |
| -0.386 | 0.00926 | 0.384 | ENSG00000273320 |  | RP11_22N192 |  |
| -0.489 | 0.00927 | 0.384 | ENSG00000289259 |  | ENSG00000289259 |  |
| -0.313 | 0.01 | 0.384 | ENSG00000290032 |  | ENSG00000290032 |  |
| 0.21 | 0.0101 | 0.384 | ENSG00000269044 |  | CTC_429P93 |  |
| 0.379 | 0.0101 | 0.384 | ENSG00000180539 |  | LINC02908 | long intergenic non-protein coding RNA 2908 |
| -0.4 | 0.0103 | 0.384 | ENSG00000253214 |  | LOC105375924 |  |
| 0.299 | 0.0111 | 0.384 | ENSG00000248429 |  | GASK1B-AS1 | GASK1B antisense RNA 1 |
| 0.482 | 0.0113 | 0.384 | ENSG00000270055 |  | CTD_3092A112 |  |
| -1.069 | 0.0115 | 0.384 | ENSG00000259354 |  | SLC30A4-AS1 |  |
| -0.366 | 0.0121 | 0.384 | ENSG00000261512 |  | RP11_46D61 |  |
| -0.218 | 0.0122 | 0.384 | ENSG00000203497 |  | PDCD4-AS1 | PDCD4 antisense RNA 1 |
| -0.398 | 0.0127 | 0.384 | ENSG00000273117 |  | INSIG1-DT |  |
| 0.257 | 0.0127 | 0.384 | ENSG00000257354 |  | MIRLET7IHG | MIRLET7I host gene |
| 0.396 | 0.0127 | 0.384 | ENSG00000262370 |  | RP11_473M209 |  |
| 0.358 | 0.0132 | 0.384 | ENSG00000281404 |  | LINC01176 | long intergenic non-protein coding RNA 1176 |
| -0.437 | 0.0132 | 0.384 | ENSG00000250548 |  | LINC01303 | long intergenic non-protein coding RNA 1303 |
| -0.445 | 0.0133 | 0.384 | ENSG00000273129 |  | PACERR |  |
| -0.338 | 0.0139 | 0.384 | ENSG00000221949 |  | LINC01465 | long intergenic non-protein coding RNA 1465 |
| 0.368 | 0.0139 | 0.384 | ENSG00000229619 |  | MBNL1-AS1 | MBNL1 antisense RNA 1 |
| 0.303 | 0.014 | 0.384 | ENSG00000286796 |  | LOC105374570 |  |
| -0.433 | 0.0141 | 0.384 | ENSG00000237892 |  | KLF7-IT1 | KLF7 intronic transcript 1 |
| -1.087 | 0.0141 | 0.384 | ENSG00000251127 |  | RP11_280G91 |  |
| 0.352 | 0.0144 | 0.384 | ENSG00000270175 |  | RP11_793H1311 |  |
| 0.256 | 0.0146 | 0.384 | ENSG00000242588 |  | ENSG00000242588 |  |
| -0.481 | 0.0146 | 0.384 | ENSG00000276809 |  | RP11_10E187 |  |
| -0.34 | 0.0148 | 0.384 | ENSG00000236423 |  | LINC01134 | long intergenic non-protein coding RNA 1134 |
| -0.399 | 0.0148 | 0.384 | ENSG00000274677 |  | RP11_505K95 |  |
| 0.298 | 0.015 | 0.384 | ENSG00000225963 |  | AC0099502 |  |
| 0.48 | 0.0151 | 0.384 | ENSG00000273032 |  | DGCR5 | DiGeorge syndrome critical region gene 5 |
| -0.248 | 0.0156 | 0.384 | ENSG00000285796 |  | AL162458.1 |  |
| -0.245 | 0.0159 | 0.384 | ENSG00000271122 |  | HERPUD2-AS1 | HERPUD2 antisense RNA 1 |
| 0.533 | 0.016 | 0.384 | ENSG00000235192 |  | AC0094952 |  |
| 0.24 | 0.0161 | 0.384 | ENSG00000239653 |  | PSMD6-AS2 | PSMD6 antisense RNA 2 |
| -0.325 | 0.0166 | 0.384 | ENSG00000289028 |  | ENSG00000289028 |  |
| 0.309 | 0.0169 | 0.384 | ENSG00000228107 |  | AP0006929 |  |
| -0.606 | 0.017 | 0.384 | ENSG00000283633 |  | AC005301.9 |  |
| -0.334 | 0.017 | 0.384 | ENSG00000229951 |  | FOSL2-AS1 | FOSL2 antisense RNA 1 |
| 0.337 | 0.017 | 0.384 | ENSG00000237877 |  | LINC01473 |  |
| -0.388 | 0.0173 | 0.384 | ENSG00000273341 |  | RP5_899E91 |  |
| -0.252 | 0.0174 | 0.384 | ENSG00000259642 |  | ST20-AS1 | ST20 antisense RNA 1 |
| 0.512 | 0.0175 | 0.384 | ENSG00000281344 |  | HELLPAR | HELLP associated long non-coding RNA |
| -0.323 | 0.0178 | 0.384 | ENSG00000214900 |  | LINC01588 | long intergenic non-protein coding RNA 1588 |
| 0.451 | 0.0179 | 0.384 | ENSG00000264659 |  | LOC105371894 |  |
| 0.334 | 0.018 | 0.384 | ENSG00000264304 |  | RP11_20B247 |  |
| -0.245 | 0.0181 | 0.384 | ENSG00000265975 |  | CTB_41I62 |  |
| -0.352 | 0.0181 | 0.384 | ENSG00000205181 |  | LINC00654 | long intergenic non-protein coding RNA 654 |
| -0.414 | 0.0182 | 0.384 | ENSG00000234261 |  | RP11_146I21 |  |
| -0.285 | 0.0184 | 0.384 | ENSG00000271614 |  | ATP2B1-AS1 | ATP2B1 antisense RNA 1 |
| 0.307 | 0.0185 | 0.384 | ENSG00000289396 |  | ENSG00000289396 |  |
| 0.293 | 0.0187 | 0.384 | ENSG00000258424 |  | RP11_471B222 |  |
| 0.327 | 0.0189 | 0.384 | ENSG00000228280 |  | RP11_367B62 |  |
| 0.126 | 0.0191 | 0.384 | ENSG00000257621 |  | PSMA3-AS1 | PSMA3 antisense RNA 1 |
| 0.475 | 0.0192 | 0.384 | ENSG00000269514 |  | RP11_370I1012 |  |
| -0.352 | 0.0194 | 0.384 | ENSG00000267480 |  | LINC02979 | long intergenic non-protein coding RNA 2979 |
| 0.415 | 0.0195 | 0.384 | ENSG00000270605 |  | RP5_1092A34 |  |
| 0.283 | 0.0197 | 0.384 | ENSG00000268471 |  | MIR4453HG | MIR4453 host gene |
| 0.266 | 0.0199 | 0.384 | ENSG00000245498 |  | MSANTD2-AS1 | MSANTD2 antisense RNA 1 |
| -0.865 | 0.0199 | 0.384 | ENSG00000272512 |  | RP11_54O717 |  |
| -0.328 | 0.0203 | 0.384 | ENSG00000247400 |  | DNAJC3-DT |  |
| -1.414 | 0.0204 | 0.384 | ENSG00000250334 |  | LINC00989 |  |
| -0.626 | 0.0205 | 0.384 | ENSG00000266709 |  | MGC12916 | uncharacterized protein MGC12916 |
| -0.175 | 0.0206 | 0.384 | ENSG00000177410 |  | ZFAS1 | ZNFX1 antisense RNA 1 |
| 0.205 | 0.0209 | 0.384 | ENSG00000278784 |  | RP11_468E211 |  |
| -0.328 | 0.0211 | 0.384 | ENSG00000258101 |  | RP11_977B102 |  |
| -0.287 | 0.0212 | 0.384 | ENSG00000286153 |  | AP000331.1 |  |
| -0.242 | 0.0212 | 0.384 | ENSG00000289138 |  | ENSG00000289138 |  |
| -0.275 | 0.0214 | 0.384 | ENSG00000273174 |  | RP11_434H66 |  |
| 0.251 | 0.0216 | 0.384 | ENSG00000231890 |  | DARS1-AS1 | DARS1 antisense RNA 1 |
| 0.239 | 0.0226 | 0.392 | ENSG00000260565 |  | ERVK13-1 | endogenous retrovirus group K13 member 1 |
| 0.327 | 0.0227 | 0.392 | ENSG00000232912 |  | RERE-AS1 | RERE antisense RNA 1 |
| -0.159 | 0.0229 | 0.392 | ENSG00000260032 |  | NORAD | non-coding RNA activated by DNA damage |
| 0.343 | 0.023 | 0.392 | ENSG00000284719 |  | AL033527.5 |  |
| -0.464 | 0.023 | 0.392 | ENSG00000289080 |  | ENSG00000289080 |  |
| 0.32 | 0.0231 | 0.392 | ENSG00000285999 |  | AC025442.2 |  |
| 0.283 | 0.0243 | 0.407 | ENSG00000259881 |  | LOC101927793 | uncharacterized LOC101927793 |
| 0.321 | 0.0244 | 0.407 | ENSG00000197989 |  | SNHG12 | small nucleolar RNA host gene 12 |
| -0.223 | 0.0251 | 0.411 | ENSG00000224307 |  | LINC02975 |  |
| -0.424 | 0.0252 | 0.411 | ENSG00000286887 |  | AL355881.1 |  |
| -0.32 | 0.0253 | 0.411 | ENSG00000234807 |  | JUN-DT |  |
| 0.292 | 0.0254 | 0.411 | ENSG00000273507 |  | RP11_50D164 |  |
| 0.29 | 0.0259 | 0.416 | ENSG00000288738 |  | ENSG00000288738 |  |
| 0.209 | 0.0262 | 0.416 | ENSG00000255717 |  | SNHG1 | small nucleolar RNA host gene 1 |
| -0.253 | 0.0268 | 0.42 | ENSG00000231721 |  | LINC-PINT | long intergenic non-protein coding RNA, p53 induced transcript |
| 0.269 | 0.0268 | 0.42 | ENSG00000289156 |  | LOC107985892 |  |
| -0.184 | 0.0272 | 0.42 | ENSG00000265666 |  | RARA-AS1 | RARA antisense RNA 1 |
| 0.243 | 0.0276 | 0.42 | ENSG00000236144 |  | TMEM147-AS1 | TMEM147 antisense RNA 1 |
| -0.266 | 0.028 | 0.42 | ENSG00000260805 |  | RP11_61J195 |  |
| 0.162 | 0.0282 | 0.42 | ENSG00000259366 |  | CTD_2647L44 |  |
| 0.236 | 0.0283 | 0.42 | ENSG00000226419 |  | SLC16A1-AS1 | SLC16A1 antisense RNA 1 |
| 0.234 | 0.0286 | 0.42 | ENSG00000196295 |  | GARS1-DT |  |
| 0.278 | 0.0288 | 0.42 | ENSG00000261366 |  | MANEA-DT | MANEA divergent transcript |
| -0.672 | 0.0289 | 0.42 | ENSG00000289150 |  | ENSG00000289150 |  |
| -0.432 | 0.0289 | 0.42 | ENSG00000228013 |  | IL6R-AS1 | IL6R antisense RNA 1 |
| -0.263 | 0.0292 | 0.42 | ENSG00000228686 |  | RP11-492I21.1 |  |
| -0.459 | 0.0293 | 0.42 | ENSG00000289573 |  | ENSG00000289573 |  |
| -0.349 | 0.0297 | 0.42 | ENSG00000277855 |  | LOC105377155 |  |
| -0.289 | 0.0297 | 0.42 | ENSG00000214783 |  | POLR2J4 | RNA polymerase II subunit J4, pseudogene |
| -0.29 | 0.0299 | 0.42 | ENSG00000288973 |  | ENSG00000288973 |  |
| 0.42 | 0.0301 | 0.42 | ENSG00000260378 |  | LOC105371090 |  |
| 0.371 | 0.0302 | 0.42 | ENSG00000287875 |  | LOC107984043 |  |
| -0.294 | 0.0303 | 0.42 | ENSG00000289340 |  | ENSG00000289340 |  |
| 0.234 | 0.0304 | 0.42 | ENSG00000225855 |  | RUSC1-AS1 | RUSC1 antisense RNA 1 |
| -0.273 | 0.0309 | 0.421 | ENSG00000228261 |  | ITPRIP-AS1 | ITPRIP antisense RNA 1 |
| -0.423 | 0.031 | 0.421 | ENSG00000289123 |  | ENSG00000289123 |  |
| -0.249 | 0.0316 | 0.423 | ENSG00000288999 |  | ENSG00000288999 |  |
| -0.65 | 0.0317 | 0.423 | ENSG00000178803 |  | ADORA2A-AS1 | ADORA2A antisense RNA 1 |
| -0.302 | 0.032 | 0.423 | ENSG00000267317 |  | CTB_25B1312 |  |
| -0.429 | 0.0321 | 0.423 | ENSG00000289377 |  | ENSG00000289377 |  |
| 0.574 | 0.0321 | 0.423 | ENSG00000275481 |  | RP11_474P26 |  |
| 0.381 | 0.0324 | 0.424 | ENSG00000260563 |  | RP13_516M141 |  |
| 0.546 | 0.0329 | 0.428 | ENSG00000289788 |  | ENSG00000289788 |  |
| -0.35 | 0.0333 | 0.431 | ENSG00000231233 |  | CFAP58-DT | CFAP58 divergent transcript |
| -0.186 | 0.034 | 0.433 | ENSG00000286532 |  | PARTICL | promoter of MAT2A antisense radiation-induced circulating long non-coding RNA |
| 0.255 | 0.0341 | 0.433 | ENSG00000289698 |  | ENSG00000289698 |  |
| -0.27 | 0.0349 | 0.433 | ENSG00000286076 |  | AC005050.3 |  |
| 0.284 | 0.0355 | 0.433 | ENSG00000258317 |  | RP11_603J245 |  |
| 0.786 | 0.0361 | 0.433 | ENSG00000288794 |  | ENSG00000288794 |  |
| 0.165 | 0.0361 | 0.433 | ENSG00000250903 |  | GMDS-DT |  |
| -0.311 | 0.0362 | 0.433 | ENSG00000290019 |  | ENSG00000290019 |  |
| 0.406 | 0.0364 | 0.433 | ENSG00000278002 |  | RP11_596C232 |  |
| 0.231 | 0.0365 | 0.433 | ENSG00000267474 |  | CTC_548K166 |  |
| -0.395 | 0.0365 | 0.433 | ENSG00000233559 |  | LINC00513 | long intergenic non-protein coding RNA 513 |
| -0.425 | 0.0366 | 0.433 | ENSG00000232618 |  | HIVEP2-DT |  |
| -0.284 | 0.0367 | 0.433 | ENSG00000261338 |  | RP11_378A131 |  |
| -0.465 | 0.037 | 0.433 | ENSG00000278493 |  | CTD_2382E56 |  |
| -0.342 | 0.037 | 0.433 | ENSG00000251230 |  | MIR3945HG | MIR3945 host gene |
| 0.206 | 0.0375 | 0.433 | ENSG00000244055 |  | AC00756610 |  |
| -0.38 | 0.0375 | 0.433 | ENSG00000250159 |  | PKD2L2-DT |  |
| -0.298 | 0.0375 | 0.433 | ENSG00000275202 |  | RP11_156K233 |  |
| 0.276 | 0.0377 | 0.433 | ENSG00000222020 |  | HDAC4-AS1 | HDAC4 antisense RNA 1 |
| -0.291 | 0.0378 | 0.433 | ENSG00000284930 |  | LOC101928143 | uncharacterized LOC101928143 |
| -0.229 | 0.0379 | 0.433 | ENSG00000261455 |  | LINC01003 | long intergenic non-protein coding RNA 1003 |
| -0.312 | 0.0379 | 0.433 | ENSG00000258820 |  | RP11_293M102 |  |
| 0.151 | 0.0385 | 0.438 | ENSG00000244625 |  | MIATNB | MIAT neighbor |
| -0.301 | 0.0391 | 0.442 | ENSG00000223478 |  | ZDHHC12-DT | ZDHHC12 divergent transcript |
| -0.894 | 0.0395 | 0.444 | ENSG00000253320 |  | MAILR | macrophage interferon regulatory lncRNA |
| -0.218 | 0.0408 | 0.444 | ENSG00000233461 |  | LOC122526782 | uncharacterized LOC122526782 |
| -0.386 | 0.0409 | 0.444 | ENSG00000235066 |  | AC0093031 |  |
| 0.231 | 0.0415 | 0.444 | ENSG00000263272 |  | CTC_524C52 |  |
| -0.311 | 0.0415 | 0.444 | ENSG00000226445 |  | THBS2-AS1 | THBS2 antisense RNA 1 |
| 0.241 | 0.0416 | 0.444 | ENSG00000267121 |  | FMNL1-DT | FMNL1 divergent transcript |
| 0.464 | 0.0417 | 0.444 | ENSG00000180953 |  | ST20 | suppressor of tumorigenicity 20 |
| -0.295 | 0.0419 | 0.444 | ENSG00000289883 |  | ENSG00000289883 |  |
| 0.273 | 0.042 | 0.444 | ENSG00000229127 |  | KANSL1L-AS1 | KANSL1L antisense RNA 1 |
| -0.451 | 0.0421 | 0.444 | ENSG00000235831 |  | BHLHE40-AS1 |  |
| 0.21 | 0.0422 | 0.444 | ENSG00000259826 |  | CDK13-DT | CDK13 divergent transcript |
| 0.315 | 0.0422 | 0.444 | ENSG00000273243 |  | CTA_217C22 |  |
| 0.127 | 0.0422 | 0.444 | ENSG00000238197 |  | PAXBP1-AS1 |  |
| -0.279 | 0.0424 | 0.444 | ENSG00000289018 |  | ENSG00000289018 |  |
| -0.223 | 0.0428 | 0.444 | ENSG00000269220 |  | LINC00528 | long intergenic non-protein coding RNA 528 |
| -0.265 | 0.043 | 0.444 | ENSG00000288996 |  | ENSG00000288996 |  |
| 0.238 | 0.0431 | 0.444 | ENSG00000286067 |  | AC004263.2 |  |
| 0.196 | 0.0434 | 0.444 | ENSG00000232442 |  | MHENCR |  |
| 0.263 | 0.0434 | 0.444 | ENSG00000270091 |  | RP11_78O72 |  |
| -0.207 | 0.0435 | 0.444 | ENSG00000284669 |  | AC092053.3 |  |
| -0.426 | 0.044 | 0.446 | ENSG00000289139 |  | ENSG00000289139 |  |
| -0.269 | 0.0443 | 0.446 | ENSG00000288852 |  | ENSG00000288852 |  |
| 0.36 | 0.0448 | 0.446 | ENSG00000228061 |  | Z830011 |  |
| 0.303 | 0.0451 | 0.446 | ENSG00000257176 |  | LOC100506606 | uncharacterized LOC100506606 |
| 0.349 | 0.0455 | 0.446 | ENSG00000269984 |  | RP11_362K145 |  |
| 0.336 | 0.0463 | 0.446 | ENSG00000229739 |  | PDC-AS1 | PDC antisense RNA 1 |
| 0.865 | 0.0466 | 0.446 | ENSG00000288910 |  | ENSG00000288910 |  |
| -0.26 | 0.047 | 0.446 | ENSG00000289126 |  | ENSG00000289126 |  |
| 0.622 | 0.0471 | 0.446 | ENSG00000177335 |  | LY6S-AS1 | LY6S antisense RNA 1 |
| -0.296 | 0.0473 | 0.446 | ENSG00000233058 |  | ATP13A3-DT |  |
| 0.29 | 0.0473 | 0.446 | ENSG00000250303 |  | LINC02762 |  |
| -0.265 | 0.0474 | 0.446 | ENSG00000235609 |  | AF1279367 |  |
| -0.339 | 0.0474 | 0.446 | ENSG00000289298 |  | ENSG00000289298 |  |
| -0.199 | 0.0474 | 0.446 | ENSG00000233901 |  | LINC01503 | long intergenic non-protein coding RNA 1503 |
| 0.373 | 0.0474 | 0.446 | ENSG00000262580 |  | RP11_334C175 |  |
| -0.276 | 0.0474 | 0.446 | ENSG00000276471 |  | RP11_398A85 |  |
| -0.298 | 0.0476 | 0.446 | ENSG00000267737 |  | AC0619922 |  |
| 0.274 | 0.0476 | 0.446 | ENSG00000226091 |  | LINC00937 | long intergenic non-protein coding RNA 937 |
| -0.482 | 0.0486 | 0.451 | ENSG00000288807 |  | ENSG00000288807 |  |
| -0.354 | 0.0486 | 0.451 | ENSG00000284948 |  | LOC254896 | uncharacterized LOC254896 |
| -0.282 | 0.0491 | 0.452 | ENSG00000274270 |  | RP11_34F207 |  |
| 0.27 | 0.0493 | 0.452 | ENSG00000230454 |  | U731662 |  |
| 0.609 | 0.0498 | 0.452 | ENSG00000248571 |  | RP11_768B222 |  |

**Table S12:** All significant networks as identified by IPA for HbA1c control (Good vs Poor)


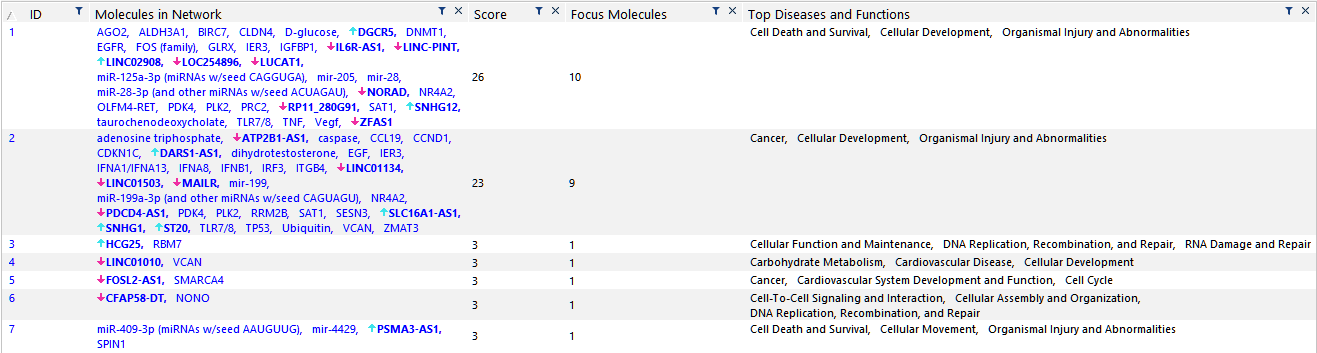


**
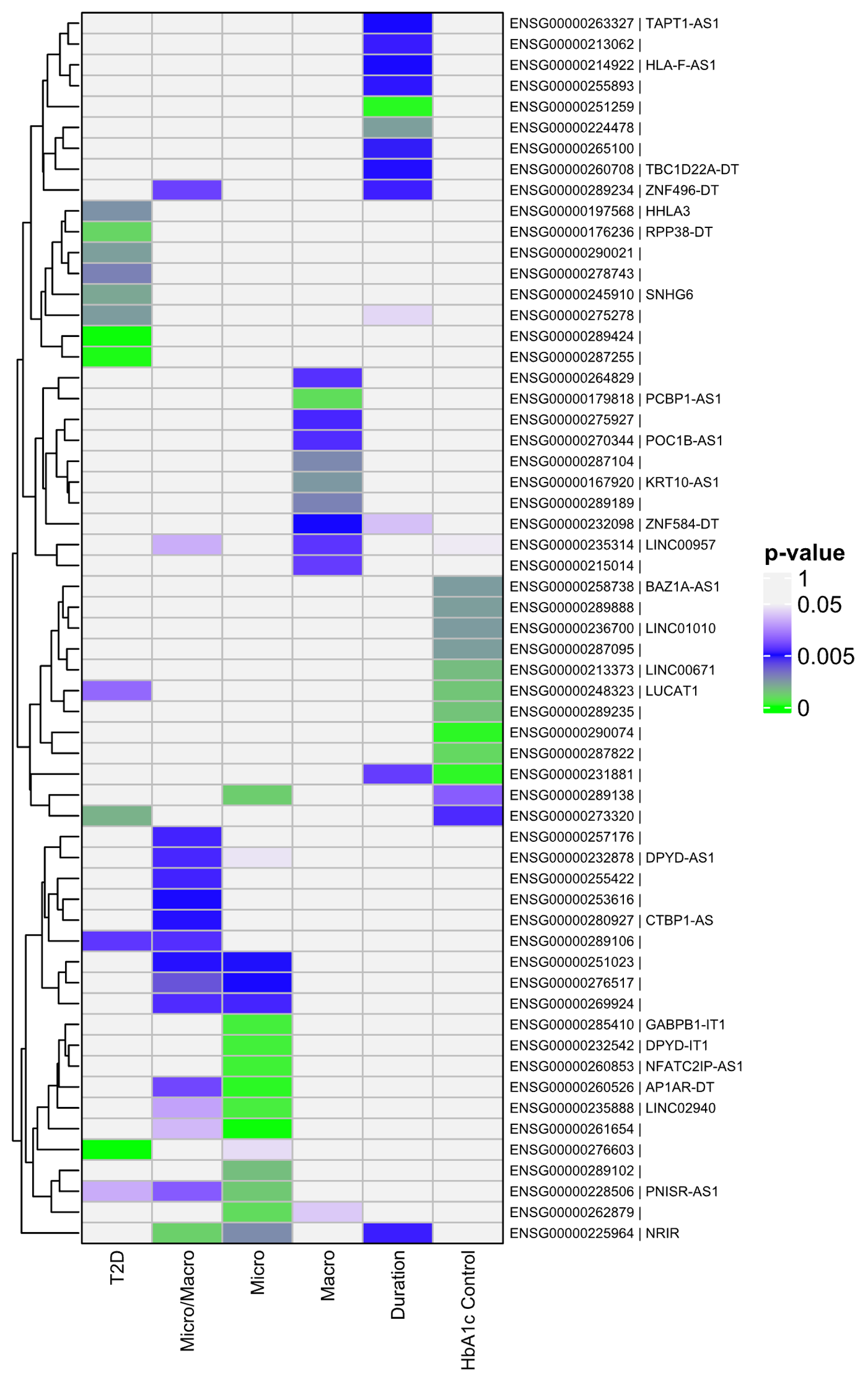
**

**Figure S2**. Clustergram of significant p-values for all differential expression comparisons. The column labels correspond to the following comparisons T2D: T2D vs. non-T2D, Micro: Microvascular only vs. no vascular disease, Macro: Macrovascular disease vs. no vascular disease, Micro/Macro: Micro and Macro vascular disease vs no vascular disease, Duration: ≥ 10 years vs < 10 years, HbA1c Control: Good vs Poor control. The row labels are the Ensembl Gene Name and common gene name separated by “|”.


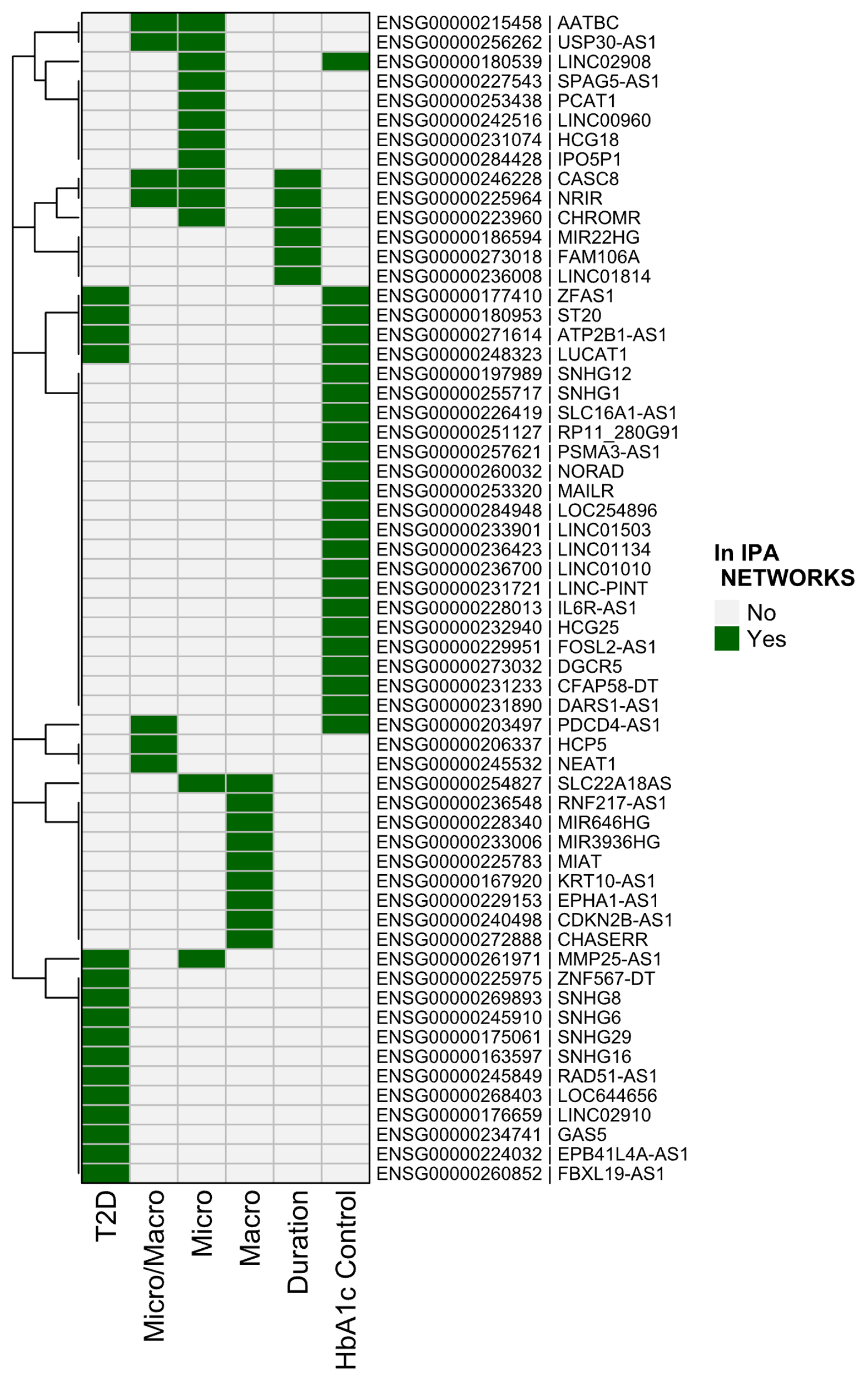


**Figure S3**. Clustergram of genes in IPA networks for all comparisons. The column labels correspond to the following comparisons T2D: T2D vs. non-T2D, Micro: Microvascular only vs. no vascular disease, Macro: Macrovascular disease vs. no vascular disease, Micro/Macro: Micro and Macrovascular disease vs. no vascular disease, Duration: ≥ 10 years vs. < 10 years. HbA1c Control: Good vs Poor control. The row labels are the Ensembl Gene Name and common gene name separated by “|”.


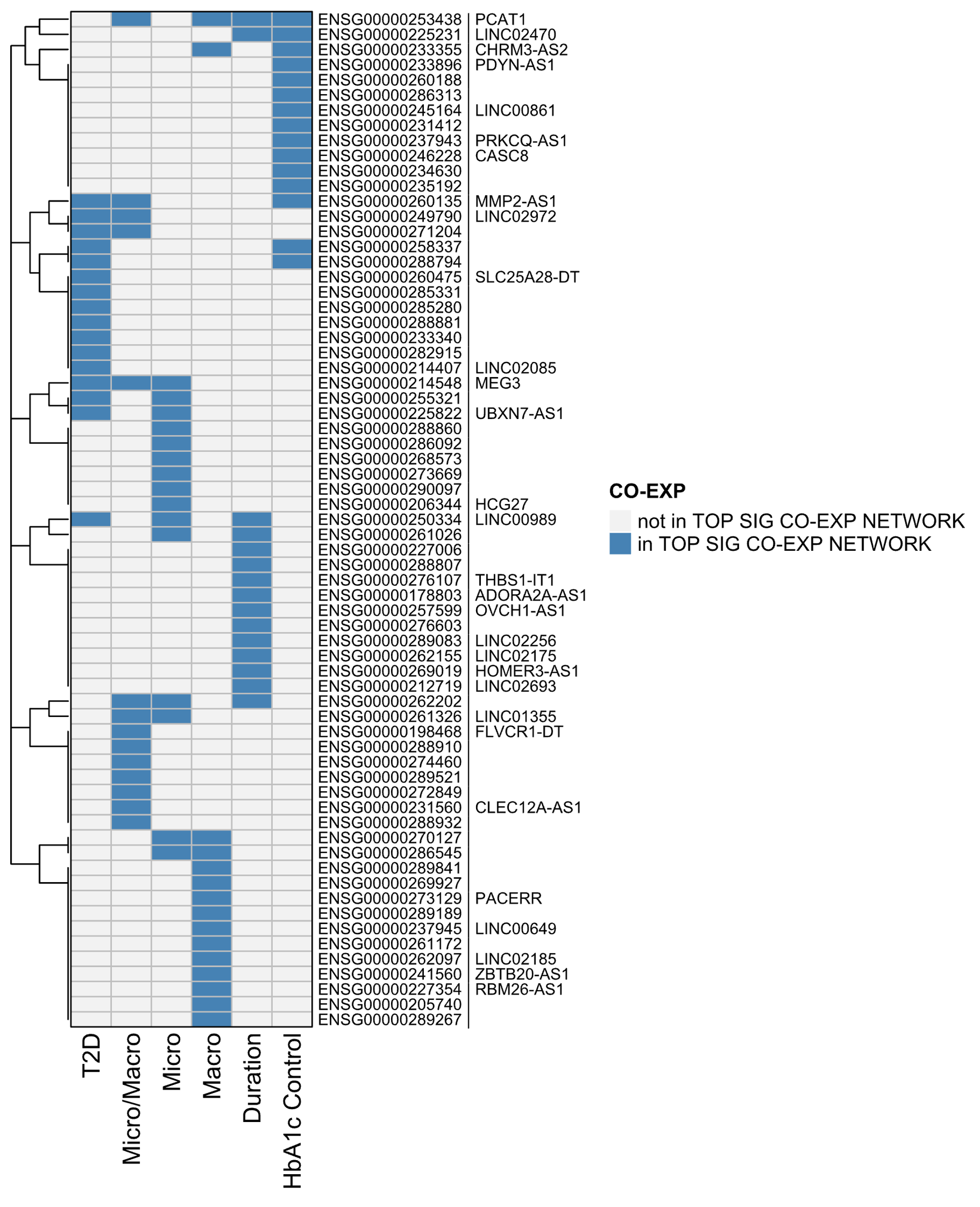


**Figure S4**. Clustergram of genes in the top significant differential co-expression comparisons. The column labels correspond to the following comparisons T2D: T2D vs. non-T2D, Micro: Microvascular only vs. no vascular disease, Macro: Macrovascular disease vs. no vascular disease, Micro/Macro: Micro and Macro vascular disease vs no vascular disease, Duration: ≥ 10 years vs < 10 years, HbA1c Control: Good vs Poor control. The row labels are the Ensembl Gene Name and common gene name separated by “|”.

References

1. Ramachandran S, Venugopal A, Sathisha K, Reshmi G, Charles S, Divya G, Chandran NS, Mullassari A, Pillai MR, Kartha CC: **Proteomic profiling of high glucose primed monocytes identifies cyclophilin A as a potential secretory marker of inflammation in type 2 diabetes**. *Proteomics* 2012, **12**(18):2808-2821.

2. Miao F, Chen Z, Zhang L, Wang J, Gao H, Wu X, Natarajan R: **RNA-sequencing analysis of high glucose-treated monocytes reveals novel transcriptome signatures and associated epigenetic profiles**. *Physiol Genomics* 2013, **45**(7):287-299.

3. Yamanaka N, Zitnan D, Kim Y-J, Adams ME, Hua Y-J, Suzuki Y, Suzuki M, Suzuki A, Satake H, Mizoguchi A *et al*: **Regulation of insect steroid hormone biosynthesis by innervating peptidergic neurons**. *Proceedings of the National Academy of Sciences of the United States of America* 2006, **103**(23):8622-8627.

4. Wang X, Chang X, Zhang P, Fan L, Zhou T, Sun K: **Aberrant Expression of Long Non-Coding RNAs in Newly Diagnosed Type 2 Diabetes Indicates Potential Roles in Chronic Inflammation and Insulin Resistance**. *Cell Physiol Biochem* 2017, **43**(6):2367-2378.

5. Zhang H, Xue C, Wang Y, Shi J, Zhang X, Li W, Nunez S, Foulkes AS, Lin J, Hinkle CC *et al*: **Deep RNA Sequencing Uncovers a Repertoire of Human Macrophage Long Intergenic Noncoding RNAs Modulated by Macrophage Activation and Associated With Cardiometabolic Diseases**. *J Am Heart Assoc* 2017, **6**(11).

6. Hu R, Xia CQ, Butfiloski E, Clare-Salzler M: **Effect of high glucose on cytokine production by human peripheral blood immune cells and type I interferon signaling in monocytes: Implications for the role of hyperglycemia in the diabetes inflammatory process and host defense against infection**. *Clin Immunol* 2018, **195**:139-148.

7. Cremer S, Michalik KM, Fischer A, Pfisterer L, Jae N, Winter C, Boon RA, Muhly-Reinholz M, John D, Uchida S *et al*: **Hematopoietic Deficiency of the Long Noncoding RNA MALAT1 Promotes Atherosclerosis and Plaque Inflammation**. *Circulation* 2019, **139**(10):1320-1334.

8. Wu H, Wen F, Jiang M, Liu Q, Nie Y: **LncRNA uc.48+ is involved in the diabetic immune and inflammatory responses mediated by P2X7 receptor in RAW264.7 macrophages**. *Int J Mol Med* 2018, **42**(2):1152-1160.

9. Leung A, Natarajan R: **Long Noncoding RNAs in Diabetes and Diabetic Complications**. *Antioxidants & Redox Signaling* 2018, **29**(11):1064-1073.

10. Sallam T, Jones M, Thomas BJ, Wu X, Gilliland T, Qian K, Eskin A, Casero D, Zhang Z, Sandhu J *et al*: **Transcriptional regulation of macrophage cholesterol efflux and atherogenesis by a long noncoding RNA**. *Nat Med* 2018, **24**(3):304-312.

11. Leung A, Amaram V, Natarajan R: **Linking diabetic vascular complications with LncRNAs**. *Vascul Pharmacol* 2019, **114**:139-144.

12. Bansal A, Balasubramanian S, Dhawan S, Leung A, Chen Z, Natarajan R: **Integrative Omics Analyses Reveal Epigenetic Memory in Diabetic Renal Cells Regulating Genes Associated With Kidney Dysfunction**. *Diabetes* 2020, **69**(11):2490-2502.

13. Das S, Reddy MA, Natarajan R: **Role of epigenetic mechanisms regulated by enhancers and long noncoding RNAs in cardiovascular disease**. *Curr Opin Cardiol* 2020, **35**(3):234-241.

14. Tanwar VS, Reddy MA, Natarajan R: **Emerging Role of Long Non-Coding RNAs in Diabetic Vascular Complications**. *Frontiers in Endocrinology* 2021, **12**:665811.

15. Natarajan R: **Epigenetic Mechanisms in Diabetic Vascular Complications and Metabolic Memory: The 2020 Edwin Bierman Award Lecture**. *Diabetes* 2021, **70**(2):328-337.

16. Reddy MA, Amaram V, Das S, Tanwar VS, Ganguly R, Wang M, Lanting L, Zhang L, Abdollahi M, Chen Z *et al*: **lncRNA DRAIR is downregulated in diabetic monocytes and modulates the inflammatory phenotype via epigenetic mechanisms**. *JCI Insight* 2021, **6**(11).

17. Ren H, Wang Q: **Non-Coding RNA and Diabetic Kidney Disease**. *DNA Cell Biol* 2021, **40**(4):553-567.

18. Dieter C, Lemos NE, Correa NRF, Assmann TS, Crispim D: **The Impact of lncRNAs in Diabetes Mellitus: A Systematic Review and In Silico Analyses**. *Front Endocrinol (Lausanne)* 2021, **12**:602597.

19. Chen Z, Natarajan R: **Epigenetic modifications in metabolic memory: What are the memories, and can we erase them?** *Am J Physiol Cell Physiol* 2022, **323**(2):C570-C582.

20. Parker DC, Wan M, Lohman K, Hou L, Nguyen AT, Ding J, Bertoni A, Shea S, Burke GL, Jacobs DR *et al*: **Monocyte miRNAs Are Associated With Type 2 Diabetes**. *Diabetes* 2022, **71**(4):853-861.

21. Liu B, Cong C, Ma Y, Ma X, Zhang H, Wang J: **Potential value of lncRNAs as a biomarker for proliferative diabetic retinopathy**. *Eye (Lond)* 2022, **36**(3):575-584.
